# Diversity of *Salmonella enterica* phages isolated from chicken farms in Kenya

**DOI:** 10.1128/spectrum.02729-23

**Published:** 2023-12-11

**Authors:** K. M. Damitha Gunathilake, Angela Makumi, Stéphanie Loignon, Denise Tremblay, Simon Labrie, Nicholas Svitek, Sylvain Moineau

**Affiliations:** 1 Département de biochimie, de microbiologie, et de bio-informatique, Faculté des sciences et de génie, Université Laval, Québec city, Quebec, Canada; 2 International Livestock Research Institute (ILRI), Nairobi, Kenya; 3 Félix d'Hérelle Reference Center for Bacterial Viruses, Université Laval, Québec city, Quebec, Canada; 4 SyntBioLab Inc., Lévis, Canada; Institut National de Santé Publique du Québec, Sainte-Anne-de-Bellevue, Québec, Canada

**Keywords:** bacteriophages, Kenya, *Salmonella*, genome analysis, poultry, CRISPR

## Abstract

In this study, we describe the characterization of 67 *Salmonella enterica* phages isolated from Kenya. Specifically, the phages were isolated from chicken feces as well as from swabs and water samples obtained from chicken farms and slaughterhouses in Nairobi and Kiambu counties. Genome sequencing and analysis revealed that 59 phage isolates were distinct and could be grouped into 17 clusters belonging to four major types of phages (MT1–4). Further taxonomic analysis showed that each MT represents one phage genus. Of the 17 selected representative genomes (one per cluster), seven phages belong to the MT1 group and to the genus *Jerseyvirus*. The MT2 cluster contained eight representative phages that were similar to Escherichia phage T5 and belong to the *Tequintavirus* genus. The unique MT3 cluster with one phage representative belongs to the *Zindervirus* genus, while the sole MT4 phage isolate is a member of the *Dhillonvirus* genus. All representative phages are siphophages, except MT3 phage which is a podophage. All genomes do not contain genes associated with lysogeny and therefore, they are considered exclusively lytic. No known virulence genes or antibiotic resistance genes were detected in any of the genomes. Phage DNA termini analyses showed that MT2 phages have a direct terminal repeat-based packaging mechanism while MT1 has a headful packaging mechanism. Host range studies showed that phages belonging to MT1 and MT3 appear to only infect *Salmonella* strains, while MT2 and MT4 phages were also capable of infecting *Escherichia* and *Shigella* strains.

**IMPORTANCE:**

Non-typhoidal *Salmonella enterica* infections are one of the leading causes of diarrhoeal diseases that spread to humans from animal sources such as poultry. Hence, keeping poultry farms free of *Salmonella* is essential for consumer safety and for a better yield of animal products. However, the emergence of antibiotic resistance due to over usage has sped up the search for alternative biocontrol methods such as the use of bacteriophages. Isolation and characterization of novel bacteriophages are key to adapt phage-based biocontrol applications. Here, we isolated and characterized *Salmonella* phages from samples collected at chicken farms and slaughterhouses in Kenya. The genomic characterization of these phage isolates revealed that they belong to four ICTV (International Committee on Taxonomy of Viruses) phage genera. All these phages are lytic and possibly suitable for biocontrol applications because no lysogenic genes or virulence factors were found in their genomes. Hence, we recommend further studies on these phages for their applications in *Salmonella* biocontrol.

## INTRODUCTION

*Salmonella enterica* is one of the major causes of invasive infections that are responsible for diarrhoeal diseases worldwide (1). There is also a wide range of *S. enterica* serotypes that cause human salmonellosis which are generally categorized into two groups: typhoidal and non-typhoidal. The typhoidal group includes the Typhi and Paratyphi serotypes. The non-typhoidal group includes several serotypes such as Enteritidis, Kentucky, and Heidelberg to name a few and they primarily spread to humans from animal sources (2). A recognized source of non-typhoidal salmonellosis is contaminated poultry and eggs (3, 4). Due to the current interest in reducing the use of antibiotics in animal farming (5 – 7) while still maintaining high safety standards and increasing yields of poultry production (8), the possibility of using specific bacterial viruses known as bacteriophages or phages as part of a biocontrol strategy (6, 9) is gaining popularity.

Biocontrol applications of phages almost dates back to their co-discovery by Felix d'Herelle, who isolated a phage and showed its antagonistic effect against another member of the *Enterobacteriaceae* family, namely *Shigella* (10). Phages are obligate parasites with high specificity to their host bacterial strains and with usually no pathogenic effects on humans and animals (10). The host-specificity of phages is a critical attribute as demonstrated by the ability to exploit them for bacterial identification purposes and for the development of rapid bacterial sensing methods (11). Furthermore, a century of phage research have paved the way to our understanding of their biology (12). The abundance and diversity of phages are also contributing to the interest in exploration of their potential as biocontrol agents (13). Phage genome characterization in the past two decades, especially after the emergence of next-generation DNA sequencing technologies, revealed t impressive genetic diversity among phage populations and confirmed the mosaicism in their genome architecture. Such genomic diversity and mosaicism are real-time evidences of an active evolution of the phage population and adaptations to different hosts and ecosystems (14).

Several *Salmonella* phages have been isolated worldwide over the last decades and the International Committee on Taxonomy of Viruses (ICTV) already recognizes more than 50 genera of *Salmonella* phages. These *Salmonella* phages were isolated from a variety of niches, including agricultural settings such as dairy (15), swine (16), and poultry farms (16, 17), to name a few. The diversity of these *Salmonella* phages was also studied using many phenotypic and genotypic features such as morphology (17), host range (15), receptor diversity (18), restriction profiling or random amplification of polymorphic DNA (16), genome sizes (15), and whole-genome sequencing (19).

Once efficient phage isolation and characterization protocols are in place, several phages infecting strains of the same bacterial species can be readily isolated. For example, 55 distinct *Salmonella* phages were isolated from swine (33 phage isolates) and chicken farms (22 isolates) located in diverse regions of Spain (16). These phages were isolated using 67 *Salmonella* strains of the Enteritidis and Typhimurium serovars (16). On the other hand, 25 phages were isolated from Korean chicken farms using the same *S. enterica* Typhimurium host (18). In another study, 108 *Salmonella* phages infecting seven non-typhoidal *Salmonella* serotypes were isolated from dairy farms in NY, USA (15). These phages were clustered into 11 groups, with considerable variations within each group (15). Genome sequencing of a subset of these phages revealed two novel *Salmonella* phages, while the rest resembled phage genera that have been already reported (19).

Strains of *Salmonella* can also carry diverse prophages, which may lead to cell lysis if functional and induced. A study reported 130 unique prophage sequences identified in 118 strains of *Salmonella* and 117 strains of *Escherichia coli*. The diversity was considerable as the average nucleotide identity (ANI) was less than 50% between the 130 prophages. The existence of prophages may also alter the host range of some phages due to superinfection immunity phenomenon (19). Several other phage defense mechanisms have been identified in *Salmonella* using bioinformatic tools (19). Although temperate phages are part of the viral diversity infecting *Salmonella*, they are usually excluded from therapeutic applications. Indeed, temperate phages can integrate their genomes into the host genome (prophage) or be independently replicating as a plasmid-like structure. Because of their tendency to stay dormant in the host and to cause lysogenic conversion by introducing genes coding for virulence factors or phage resistance to the host, these phages are not preferred for phage therapy (20, 21).

Some of the characterized lytic *Salmonella* phages have been already exploited in commercial products for the biocontrol of *Salmonella* (22, 23) and even tested on chicken farms (22). However, host range studies have shown that the susceptibility of *Salmonella* strains to commercial phage cocktails is highly variable depending on the phages used and the targeted bacterial strains (24). Also, single phages may have narrow host ranges. Hence, it is often essential to have a repository of lytic phages that can infect the *Salmonella* strains found in the targeted niche and geographic location. Combining several carefully selected phages into a cocktail would increase the overall host range and could also reduce the emergence of phage-resistant mutants (25, 26). The latter is one of the causes of phage therapy or biocontrol failures. To overcome this issue, combining a group of genetically diverse phages that utilize different host receptors for adsorption has become a common strategy (27). Hence, this study aimed to characterize non-typhoidal *Salmonella* phages isolated from chicken farms in Kenya to understand their overall diversity and identify distinct phages for future biocontrol or therapeutic applications.

## RESULTS AND DISCUSSION

### *Salmonella* phage isolates

To isolate *Salmonella*-infecting phages, we used a set of 16 *Salmonella* strains that were previously isolated from the same environments in Kenya (see Materials and Methods and Tables S1 and S2 for details). The serological testing and CRISPR array sequencing showed that these 16 *Salmonella* strains belong to three serotypes: Enteritidis (seven isolates), Kentucky (four isolates), and Heidelberg (five isolates). Using 631 samples (feces, water, and swabs) collected in chicken farms and slaughterhouses in Kenya, 67 phage isolates capable of infecting the aforementioned *S. enterica* hosts were isolated (Table S1).

### Phage genome sequencing

To determine if these 67 isolates were distinct, their genomes were sequenced and annotated (GenBank accession numbers are provided in Table S1). Then, we performed comparative genome analysis, using ANI and ANI based distance matrix (28, 29). The ANI analysis revealed that 8 of the 67 phages (Table 1) were identical leaving 59 distinct phages. Genome pairwise analyses indicated that these phages could be clustered into four major types (MTs): MT1 to MT4, with either 0 or a limited nucleotide identity between them (Table 1; Fig. S1). However, the genomes within each MT were highly similar, with 93–98% ANI. This high level of ANI indicated that each MT likely consists of phage isolates that belong to the same genus. Still using ANI, these phages were further categorized into 17 subtypes (STs) of highly similar phages with >99% ANI to each other. There were seven STs in MT1, eight STs in MT2, one ST in MT3, and one in MT4 (Table 1). The majority of isolates (26/67, 39%) belong to MT1, specifically to ST1-1. MT3 included one ST (with four isolates) and MT4 had only one isolate.

**TABLE 1:** *Salmonella* phage types based on average nucleotide identity

Major type (MT)	Subtype (ST)	Phage isolates (coded K1–K69)^*a*^	ANI% between genomes within ST	100% identical genomes in the subtype^*a*^	ANI% between genomes within MT
1	1-1	**K2**, K3, K4, K5, K13, K23, K24, K25, K27, K28, K48, K49, K50, K51, K52, K55, K57, K59, K60, K62, K63, K64, K66, K67, K68, K69	99.3**–**99.7	-	95–98%
	1-2	K14, K29, **K53**, K65	99.9**–**99.98	-	
	1-3	K7, K54, **K6**/K8/K61	99.99**–**100	K6 = K8 = K61	
	1-4	K1, K10, K46, **K22**/K58	99.99**–**100	K10 = K22 = K46 = K58	
	1-5	**K9**, K26	99.99	-	
	1-6	**K56**	-	-	
	1-7	**K47**	-	-	
2	2-1	**K16**, K17, K34, K41, K33/K35/K39/K40	99.91**–**99.99	K33 = K35 = K39 = K40	93–99%
	2-2	**K36**, K42	99.99	-	
	2-3	**K15**	-	-	
	2-4	**K18**	-	-	
	2-5	**K20**	-	-	
	2-6	**K37**, K43	99.99	-	
	2-7	**K44**	-	-	
	2-8	**K38**, K45	-	-	
3	-	K11, **K30**, K31, K32	99.95**–**99.98	-	
4	-	**K19**	-	-	

^*a*^ Reference genomes from each subtype are shown in bold and underlined. K12 and K21 were no longer infectious.

**Fig 1:**
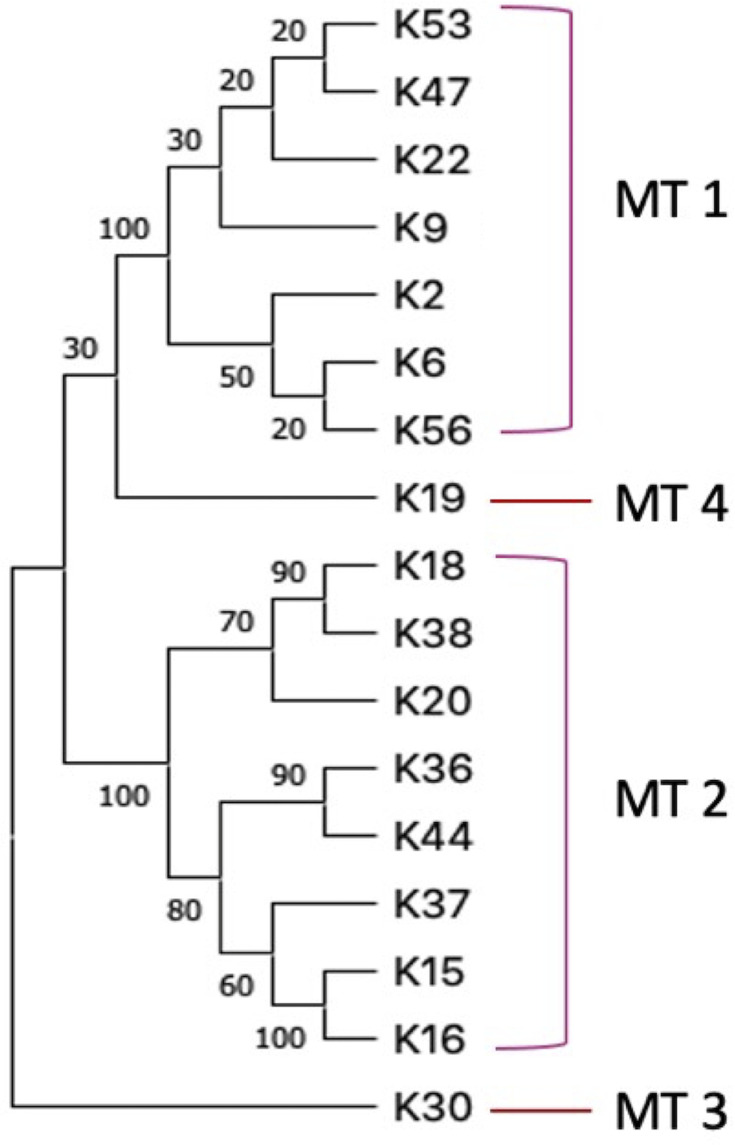
Bootstrapped maximum likelihood phylogenetic tree constructed with major capsid protein sequences of the reference genomes for phages isolated in Kenya.

One phage per ST was randomly selected for further analysis. To assist with referencing, the MTs and STs were numbered as indicated in Table 1. The general genomic features such as length (41–44 kb), GC content (47–54%), number of predicted protein-coding genes (CDSs, 57–70), and the absence of tRNA genes, did not show a noteworthy difference among phage members of the MTs 1, 3, and 4 (Table 2). However, the genomes of MT2 phages were markedly different, with a low CG content (39%), a larger size (106–109 kb) and 9–17 tRNAs (Table 2). MT2 genomes also had long direct terminal repeats (DTRs) ranging from 8,451 to 10,020 bp (Table 2). The maximum likelihood phylogenetic analysis with the major capsid protein sequences of the 17 reference genomes confirmed the presence of four distinct groups among this set of *Salmonella* phages (Fig. 1).

**TABLE 2:** Genomic features of the 17 reference phage genomes

Major type (MT)	Subtype (ST)	Reference genome^*a*^	Sequence length (bp) after the assembly	DTR length (bp)	Estimated genome length (bp)	GC %	Number of predicted CDSs (RAST)	No. of tRNA (RAST)	GenBank accession number
1	1-1	K2	43,066	-	43,066	49.9	66	0	OQ291020
	1-2	K53	41,541	-	41,541	50.1	64	0	OQ291027
	1-3	K6	43,039	-	43,039	49.8	67	0	OQ291021
	1-4	K22	42,734	-	42,734	49.9	67	0	OQ291024
	1-5	K9	43,087	-	43,087	49.9	66	0	OQ291022
	1-6	K56	42,795	-	42,795	49.9	70	0	OQ291028
	1-7	K47	42,735	-	42,735	50.0	68	0	OQ291027
2	2-1	K16	106,672	9,371	116,043	39.1	151	17	OQ291030
	2-2	K36	106,205	8,641	114,846	39.2	140	12	OQ291033
	2-3	K15	108,076	9,488	117,564	39.1	153	17	OQ291029
	2-4	K18	109,965	10,020	119,985	39.1	155	15	OQ291031
	2-5	K20	106,925	9,232	116,157	39.1	147	09	OQ291032
	2-6	K37	109,165	9,846	119,011	39.3	153	16	OQ291034
	2-7	K44	106,562	8,451	115,013	39.0	144	14	OQ291036
	2-8	K38	108,178	9,560	117,738	39.4	153	10	OQ291035
3		K30	44,304	-	44,304	47.8	57	0	OQ291025
4		K19	44,834	-	44,834	54.8	64	0	OQ291023

^*a*^ GenBank records show the organism names as *Salmonella* phage Kenya-K2 and likewise.

### Genome structure, organization, and subtype comparison

**Fig 2:**
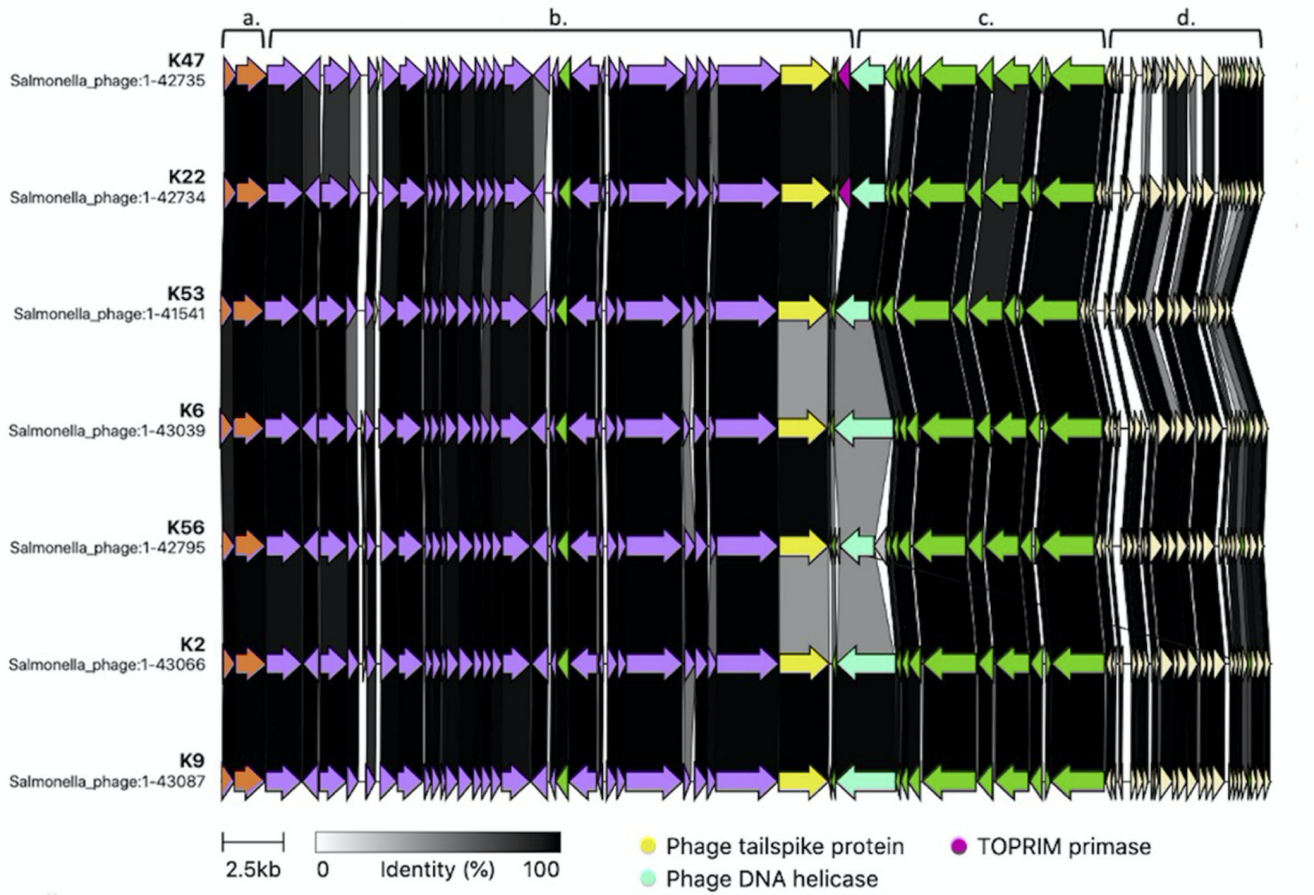
Protein alignment of MT1 genomes. (**a**) Phage proteins involved in packaging (orange), (**b**) Phage structural proteins (purple), (**c**) Phage proteins involved in replication (green), and (**d**) Other proteins (off-white).

**Fig 3:**
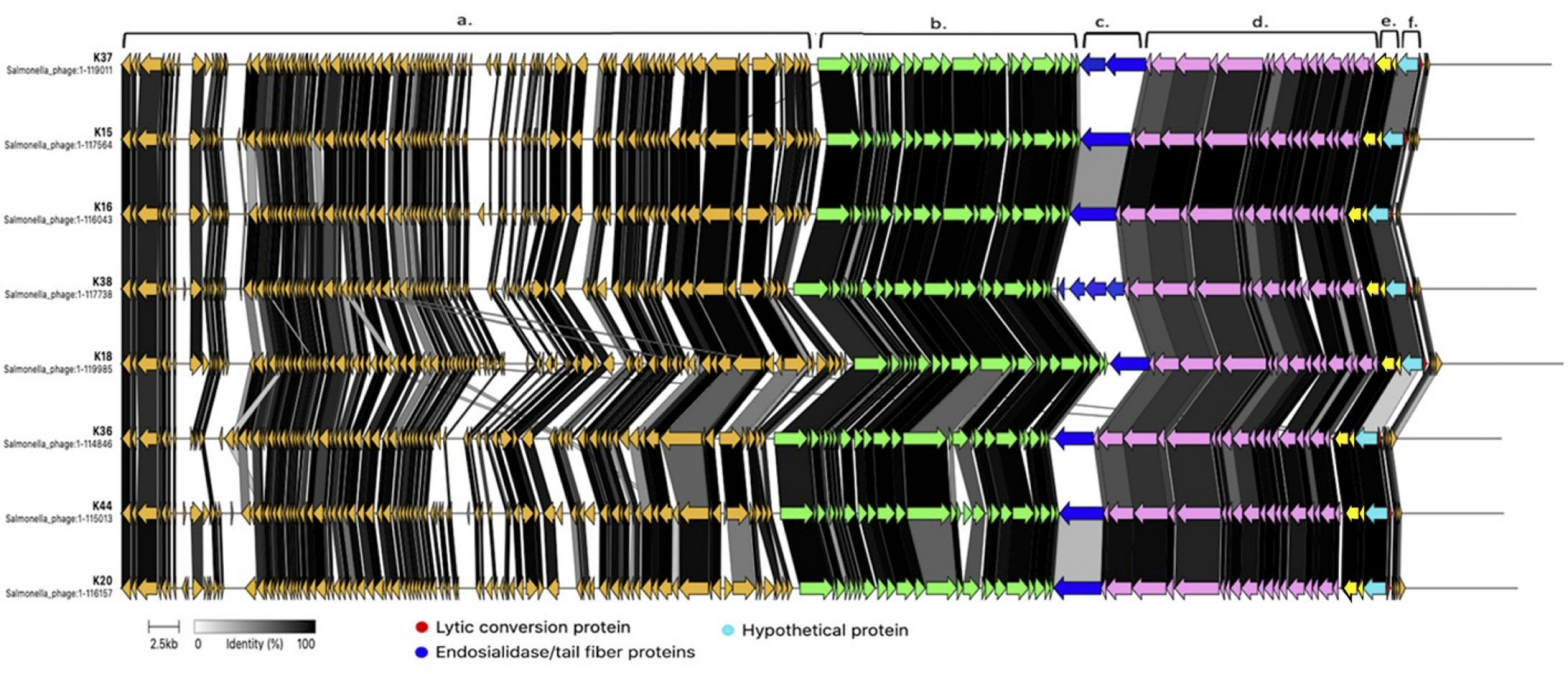
Protein alignment of MT2 genomes. (**a**) Genomic region with phage proteins of unknown function (orange), (**b**) Region with phage proteins involved in replication (green), (**c**) Single protein or multiple proteins annotated as tail fibers or endosialidase, which is found in the tail fibers of some phages, (**d**) Phage structural proteins (pink), (**e**) Packaging proteins (yellow), (**f**) Lytic conversion protein, and a hypothetical protein.

**Fig 4:**
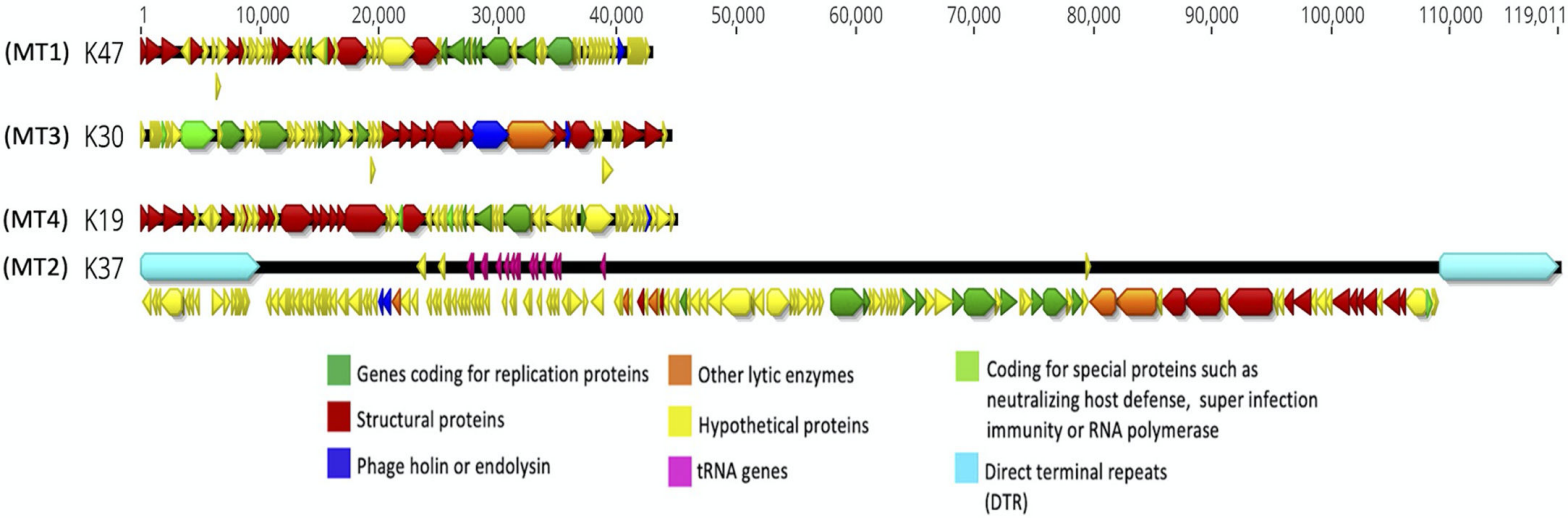
Genome arrangement of one selected reference phage per each of the four MT groups. Phage K47 from MT1, K37 from MT2, K30 from MT3, and K19 from MT4.

**Fig 5:**
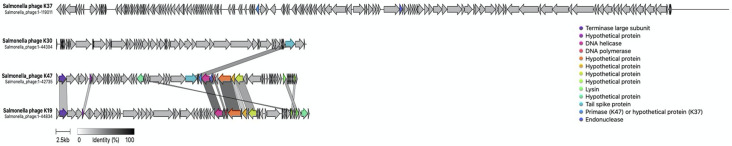
Protein comparison among four selected reference phage genomes to represent each of the four MT groups, namely phage K47 (MT1), K37 (MT2), K30 (MT3), and K19 (MT4).

The synteny and the structure of these genomes were similar among the STs within each MT (Fig. 2 and 3; Fig. S2A and B) but different between MTs (Fig. 4 and 5; Fig. S2C). For example, all the phage genomes belonging to MT1 showed the same synteny and similar proteomes (Fig. 2). The deduced proteins from the MT1 genomes share more than 90% identity to homologs except for the tail spike protein (homologs of K47_032), the TOPRIM primase (homologs of K47_035), and the DNA helicase (homologs of K47_036). TOPRIM primases were found only in phages K47 (ST 1-7) and K22 (ST 1-4). The tail spike proteins in certain MT1 genomes (K2, K6, K53, and K56) had only 48**–**50% amino acid identity (Fig. 2, region b) to other homologs. Similarly, the DNA helicase in some MT1 genomes (K2, K6, K53, and K56) had only 50**–**52% amino acid identity (Fig. 2, region c) to homologs in other MT1 genomes.

Genomes from phages within MT2 also had similar synteny and proteomes (Fig. 3). The most notable differences were observed in the genomic region “c” as shown in Fig. 3. The lytic conversion protein (K37_150 homologs) and the adjacent hypothetical protein in genomic region “f” (K37_149 homologs) also showed notable differences. In those regions, the amino acid identity among homologs was below 50% or even below 30% in some cases. Region “c” contained genes coding for either one or multiple endosialidase homologs (e.g., K37) or multiple tail fibers with an endosialidase (e.g., K38). Endosialidase is a trimeric, polysialic acid-specific tail spike protein (30, 31). Polysialic acid capsules (32 – 34) are found in pathogenic *E. coli* strains, including the ones that are the leading causes of neonatal meningitis in humans (35). The polysialic acid capsule (32) protects the bacterium from immune responses but also provides an attachment site for phages that are equipped with endosialidase in their tail spikes (30). Although we did not include strains with confirmed polysialic acid-containing capsules in the host range experiment, MT2 phages were capable of infecting certain strains of *E. coli* (Table 3). Yet, the exact role that the viral endosialidase plays during *Salmonella* infection is unknown. It is likely that the differences in host ranges between some MT2 phages are due to variations in the endosialidase tail fiber proteins.

**TABLE 3:** Host range and titers of *Salmonella* phages on various bacteria

Bacterial strain	Phage isolate^*a*^																
	MT1							MT2								MT3	MT4
	K2 ST1-1	K53 ST1-2	K6 ST1-3	K22 ST1-4	K9 ST1-5	K56 ST1-6	K47 ST1-7	K16 ST2-1	K36 ST2-2	K15 ST2-3	K18 ST2-4	K20 ST2-5	K37 ST2-6	K44 ST2-7	K38 ST2-8	K30	K19
*Salmonella* enterica Typhi HER 1038	*1.0E04*	*1.0E03*	*1.0E03*	*1.0E04*	*1.0E03*	*1.0E03*	*1.0E05*	*5.0E02*	-	-	*1.0E03*	-	*5.5E05*	-	*5.5E03*	*1.0E04*	-
*S. enterica* Paratyphi B HER 1045	*5.5E03*	*1.0E03*	*1.0E04*	*1.0E04*	*1.0E03*	*1.0E04*	*1.0E04*	*1.0E05*	*5.5E04*	*1.0E05*	*1.0E05*	*1.0E04*	**1.0E08**	*1.0E05*	**1.0E08**	**1.0E09**	-
*S. enterica* Paratyphi B HER 1220	*5.5E03*	*1.0E03*	*1.0E04*	*1.0E04*	*1.0E03*	*1.0E04*	*1.0E04*	-	*5.5E03*	*5.0E02*	*5.0E02*	*1.0E03*	*5.1E06*	*5.5E03*	*5.1E04*	**1.0E09**	-
*S. enterica* Enteritidis S3	**1.0E10**	**1.0E09**	**5.5E09**	**5.5E09**	**1.0E09**	**5.5E09**	**1.0E10**	-	-	-	-	-	*5.5E03*	-	-	**1.0E05**	-
*S. enterica* Enteritidis S187	**5.5E09**	**1.0E09**	**5.5E09**	**1.0E10**	**1.0E09**	**5.5E09**	**1.0E10**	-	*1.0E03*	-	-	-	5.0E07	-	-	**1.0E09**	-
*S. enterica* Heidelberg S191	*1.0E04*	*5.0E02*	*5.5E03*	*1.0E05*	*5.0E02*	*1.0E04*	*1.0E05*	-	*1.0E03*	-	-	-	*5.0E02*	*1.0E03*	*5.0E02*	**1.0E09**	-
*S. enterica* Typhimurium S189	*5.5E05*	*5.5E05*	*5.5E05*	*5.5E05*	*1.0E05*	*5.5E05*	*1.0E06*	-	-	-	-	-	-	-	-	**1.0E09**	-
*S. enterica* Typhimurium S441	*1.0E06*	*1.0E05*	*1.0E06*	*5.5E06*	*5.5E04*	*1.0E06*	*5.5E06*	-	-	-	-	-	-	-	-	**1.0E09**	-
*S. enterica* Infantis S198	-	-	-	-	-	-	-	*1.0E06*	*-*	*5.5E05*	*-*	*-*	*5.5E04*	-	-	-	-
*S. enterica* Newport S2	-	-	-	-	-	-	-	**1.0E10**	-	**1.0E10**	-	-	**5.1E07**	-	-	-	-
*S. enterica* Newport S195	-	-	-	-	-	-	-	-	-	-	-	-	-	-	-	**1.0E09**	-
*Escherichia coli* B HER 1024	-	-	-	-	-	-	-	**1.0E10**	**1.0E09**	**1.0E10**	**5.5E09**	**1.0E09**	**1.0E10**	**1.0E10**	**1.0E10**	-	**1.0E09**
*E. coli* C, HER 1036	-	-	-	-	-	-	-	**1.0E10**	**5.5E09**	**1.0E10**	**5.5E09**	**1.0E09**	**1.0E10**	**1.0E10**	**1.0E10**	-	**1.0E09**
*E. coli* W3350, HER 1077	-	-	-	-	-	-	-	**1.0E10**	**5.5E09**	**1.0E10**	**5.5E09**	**1.0E09**	**1.0E10**	**1.0E10**	**1.0E10**	-	**1.0E09**
*E. coli* K12 MC4100, HER 1366	-	-	-	-	-	-	-	**1.0E10**	**1.0E10**	**1.0E10**	**5.5E09**	**1.0E09**	**5.5E09**	**5.1E09**	**5.1E09**	-	**1.0E09**
*E. coli* CSH39, HER 1290	-	-	-	-	-	-	-	**5.5E09**	**1.0E09**	**5.5E09**	**1.0E09**	**1.0E09**	**5.5E09**	**1.0E10**	**5.5E09**	-	**1.0E09**
*E. coli* K12 C600, HER 1275	-	-	-	-	-	-	-	**1.0E10**	-	**1.0E10**	**1.0E10**	-	**1.0E10**	-	**1.0E10**	-	-
*E. coli* O157:H7, HER 1256	-	-	-	-	-	-	-	-	-	-	-	-	*5.1E04*	*1.0E04*	-	-	-
*E. coli* O157:H7, HER 1268	-	-	-	-	-	-	-	-	-	-	-	-	*5.5E03*	*5.5E03*	-	-	-
*Shigella dysenteriae* HER 1020	-	-	-	-	-	-	-	**5.5E07**	*1.0E06*	*1.0E06*	*1.0E06*	*5.5E05*	**5.5E09**	*1.0E06*	*1.0E06*	-	**1.0E09**
*S. dysenteriae* HER 1031	-	-	-	-	-	-	-	**1.0E10**	**1.0E10**	**1.0E10**	**1.0E10**	**1.0E09**	**1.0E10**	**1.0E10**	**1.0E10**	-	**1.0E09**
*Shigella sonnei* HER 1043	-	-	-	-	-	-	-	**1.0E10**	-	**1.0E10**	**1.0E10**	-	**1.0E10**	-	**1.0E10**	-	-
*Shigella flexneri* HER 1521	-	-	-	-	-	-	-	-	-	-	-	-	-	-	-	-	-
*Citrobacter freundii* HER 1516	-	-	-	-	-	-	-	-	-	-	-	-	-	-	-	-	-
*C. freundii* HER 1518	-	-	-	-	-	-	-	-	-	-	-	-	-	-	-	-	-
*Erwinia amylovora* HER 1448	-	-	-	-	-	-	-	-	-	-	-	-	-	-	-	-	-
*Erwinia herbicola* HER 1172	-	-	-	-	-	-	-	-	-	-	-	-	-	-	-	-	-
*Enterobacter cloacae* HER 1067	-	-	-	-	-	-	-	-	-	-	-	-	-	-	-	-	-
*Hafnia alvei* HER 1272	-	-	-	-	-	-	-	-	-	-	-	-	-	-	-	-	-
*Kluyvera cryocrescens* HER 1400	-	-	-	-	-	-	-	-	-	-	-	-	-	-	-	-	-

^*a*^ Average phage titers ranging from 1 × 10^7–10^ were considered strong infections (bold and underlined), while 1 × 10^3–6^ were considered mild infections (italicized). The absence of plaques or a clearing of the bacterial lawn with a phage dilution higher than 10^−1^ indicated no infection (-).

The genome arrangement of one representative from each MT is shown in Fig. 4. All these genomes show a modular organization with at least two clearly visible modules. One module contains the genes encoding structural proteins (including packaging) and the other module contains the CDSs for DNA replication. Genes required for host cell lysis, such as holin, endolysin, and spanin, are usually located in close proximity to each other (36). Bioinformatic analyses were unable to locate all three genes within each genome but did reveal the presence of a gene coding for an endolysin in every genome (Fig. 4, Locus tag K47_059, K37_041, K30_042, and K19_061). Holins were readily identified in phages belonging to MT2 and MT3 (e.g., Locus tag K37_042 and K30_045). In contrast to the other MTs, phage K30 of the MT3 (Fig. 4) had its lysis genes located within the structural module. MT2 genomes had tRNA genes and long direct terminal repeats, which made them distinct from other major types (Table 2; Fig. 4).

None of the genomes in this study contained genes for lysogeny, such as integrase or plasmid-replication homologs of parABS proteins (37). Also, no known virulence factor or antibiotic resistance genes were observed in the annotated phage genomes suggesting that they may be used in biocontrol applications. However, some noteworthy genes were observed in certain MTs. Both MT1 and MT4 (K19) genomes contained superinfection immunity (Sie) proteins (locus K47_024 and other homologs of MT1 as well as K19_025 in MT4). Sie proteins prevent the same or similar phages from infecting the same host (38, 39). Similarly, the MT2 genomes contain proteins annotated as membrane lipoprotein attachment sites or lytic conversion lipoprotein precursors (K37_150 and homologs) which are also Sie proteins. For example, K37_150 had 29.9% amino acid identity (75% of query cover) with the lytic conversion lipoprotein of *E. coli* phage T5 (gene name: llp, locus_tag: T5.158), which is a Sie that inactivates host receptor proteins (39, 40). Therefore, it is possible that phages belonging to MT1, MT2, and MT4 can block a secondary infection by the same phage or other similar phage types.

Restriction-modification (R-M) systems are common bacterial defense mechanisms that limit invasion by exogenous DNA, such as phage genomes (41). Some phages have developed a strategy for avoiding recognition of their restriction sites through methylation (42). The genome of phage K19 (MT4) was found to contain a gene (K19_031) coding for an adenine methylase (Dam), which may protect from bacterial type II restriction enzymes that recognize adenine-containing sites. Similarly, phage K30 (MT3) possessed a gene (locus K30_003) coding for an Ocr-like protein. Ocr mimics the B-form of DNA to compete with target DNA to bind and trap restriction enzymes (43), leaving most of the invading phage DNA targets unaffected by R-M. Of note, phage K30 also had the broadest host range among the tested phages (Table 4).

**TABLE 4:** Host range and titer of *Salmonella* phages in hosts isolated from Kenyan samples

				Phage isolates																
				Major type: MT1							MT2								MT3	MT4
				ST1-1	ST1-2	ST1-3	ST1-4	ST1-5	ST1-6	ST1-7	ST2-1	ST2-2	ST2-3	ST2-4	ST2-5	ST2-6	ST2-7	ST2-8		
				K2 (host: Sal16)	K53 (Sal 569)	K6 (Sal 16)	K22 (Sal 177)	K9 (Sal 177)	K56 (Sal 572)	K47 (Sal 312)	K16 (Sal 157)	K36 (Sal 192)	K15 (Sal 157)	K18 (Sal 157)	K20 (Sal 157)	K37 (Sal 192)	K44 (Sal 194)	K38 (Sal 192)	K30 (Sal 187)	K19 (Sal 157)
**Kenyan *Salmonella* isolates**	**Serotype: Enteritidis**	**HG1^*a*^**	**Sal 16**	**1E10**	**1E10**	**1E10**	**1E10**	**1E10**	**1E10**	**1E10**	-	-	-	-	-	-	-	-	**1E09**	-
			**Sal 177**	**1E10**	**1E10**	**1E10**	**1E10**	**1E10**	**1E10**	**1E10**	-	-	-	-	-	-	-	-	**1E09**	-
			**Sal 568**	**1.E10**	**1E10**	**1E10**	**1E10**	**1E10**	**1E10**	**1E10**	-	-	-	-	-	**1E08**	-	-	-	-
			**Sal 569**	**1E10**	**1E10**	**1E10**	**1E10**	**1E10**	**1E10**	**1E10**	-	-	-	-	-	**1E08**	-	-	-	-
			**Sal 572**	**1E10**	**1E10**	**1E10**	**1E10**	**1E10**	**1E10**	**1E10**	-	-	-	-	-	**1E09**	-	-	-	-
			**Sal 73**	**1E10**	**1E10**	**1E10**	**1E10**	**1E10**	**1E10**	**1E10**	-	-	-	-	-	-	-	-	**1E09**	-
			**Sal 312**	**1E07**	**1E07**	**1E07**	**1E10**	**1E07**	**1E07**	**1E10**	-	-	-	-	-	-	-	-	**1E09**	-
	**Heidelberg**	**^*a*^HG5**	**Sal 157**	-	-	-	-	-	-	-	**1E10**	**1E10**	**1E10**	**1E10**	**1E10**	**1E10**	**1E09**	**1E10**	-	**1E09**
			**Sal 192**	-	-	-	-	-	-	-	**1E10**	**1E10**	**1E09**	**1E10**	**1E10**	**1E10**	**1E09**	**1E10**	-	**1E09**
			**Sal 194**	-	-	-	-	-	-	-	**1E10**	**1E10**	**1E09**	**1E10**	**1E10**	**1E10**	**1E09**	**1E10**	-	**1E09**
		**^*a*^HG4**	**Sal 187**	*1E05*	*1E05*	*1E05*	*1E04*	*1E05*	*1E05*	*1E05*	-	-	-	-	-	-	-	-	**1E10**	-
			**Sal 188**	*1E05*	*1E05*	*1E05*	*1E05*	*1E05*	*1E05*	*1E05*	-	-	-	-	-	-	-	-	**1E10**	-
	**Kentucky**	**^*a*^HG3**	**Sal 172**	-	-	-	-	-	-	-	-	-	-	-	-	-	-	-	**1E08**	-
			**Sal 181**	-	-	-	-	-	-	-	-	-	-	-	-	-	-	-	**1E08**	-
			**Sal 182**	-	-	-	-	-	-	-	-	-	-	-	-	-	-	-	**1E08**	-
		**^*a*^HG2**	**Sal 571**	-	-	-	-	-	-	-	-	-	-	-	-	-	-	-	*1E06*	-

^*a*^ Host group (HG) according to serotype and phage infection pattern. Average phage titers ranging from 1 × 10^7–10^ were considered strong infections (bold and underlined numbers), while titers ranging from 1 × 10^3–6^ were considered mild infections (italicized). The absence of plaques or a clearing of the bacterial lawn with a phage dilution higher than 10^−1^ indicated no infection (-).

### Comparative genomics and taxonomy

Although, in general, no ANI was found between MTs with local alignment tools, pairwise identity was found in specific genomic regions using the global alignment tool Mauve. For example, a global pairwise nucleotide identity of 6.2% was observed between MT1 and MT4 genomes (Fig. S2C). Also, a few proteins encoded in the genomes of MT1 and MT4 clusters shared between 48% and 66% amino acid identity (Fig. 5). Overall, these proteins included the terminase large subunit, DNA polymerase, helicase, endolysin, and some hypothetical proteins. Interestingly, MT1 (K47) is the only MT to share at least one similar protein with other MTs. The tail spike protein is 49% similar between K47_032 (MT1) and K30_55 (MT3). Phages belonging to MT1 and MT3 had overlapped host ranges (Table 4), which may be due to the similarity of tail spike proteins. Furthermore, one of the MT1 endonucleases (K47_037) and the TOPRIM primase (K47_035) showed 30% and 46% identity to an MT2 endonuclease (K37_104) and a hypothetical protein (K37_072), respectively. MT1 phages and K37 (MT2) also coinfect certain *Salmonella* strains from Kenya. It is worth noting that the amino acid sequences of K47_032 and K47_035 homologs showed subtype differences, as mentioned earlier. The amino acid sequences of these proteins were over 90% similar among MT1 subtypes, except for ST1-1 (K2), ST1-2 (K53), ST1-3 (K6), and ST1-6 (K56), which showed 48–50% amino acid identity to homologs (Fig. 2). The coinfection of the same hosts by MT1 and either MT3 or MT2 phages likely led to genetic exchange among these phages (44) causing these differences.

**Fig 6:**
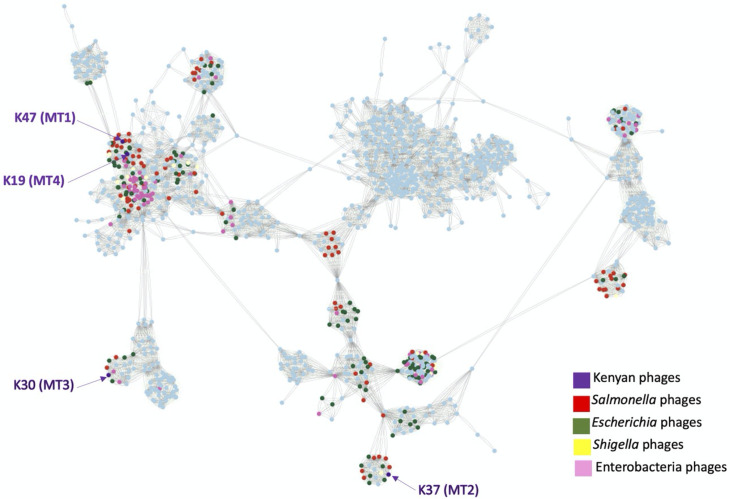
Phage genome network generated with vConTACT2 0.9.19, which includes phage representatives of the four *Salmonella* MTs (MT1 to MT4) from Kenya.

A BLASTn search of Kenyan phage genomes against NCBI database revealed significant (≥90%) identities with previously published phage genomes for all four MTs. These other phage genomes were isolated from different countries, such as Poland: MT1-like phages (45), Hungary: MT2-like (46), USA: MT3-like (47), China: MT4-like (48), and so on. Top NCBI BLASTn hits to the Kenyan reference phages are shown in Table S14 and illustrated in Fig. S3. A vConTACT2 analysis revealed that the four MTs belong to four genera of phages (Fig. 6). Phages within the MT1 cluster belong to the genus called *Jerseyvirus*, which contains several siphophages infecting *Salmonella*. Phages grouped in MT2 belong to the genus *Tequintavirus*. Members of this genus include the Escherichia phage T5 as well as other phages infecting *E. coli*, *Salmonella* and/or *Shigella* strains. Phages clustered in MT3 belong to the genus *Zindervirus*, which includes a group of podophages infecting *Salmonella* and *E. coli* strains. Finally, the sole phage within MT4 is a member of genus *Dhillonvirus*, which is a genus that comprises phages infecting mostly *E. coli* strains. According to GenBank data, K19 (MT4) may be the first phage of this genus isolated using a *Salmonella* host. Finally, the genome network generated by vConTACT2 also suggested that MT1 and MT4 phages may share a common taxonomic group that lies between genus and class. To our knowledge, Jerseyviruses (MT1) and Dhillonviruses (MT4) have not been assigned to a viral family yet.

### Electron microscopy

**Fig 7:**
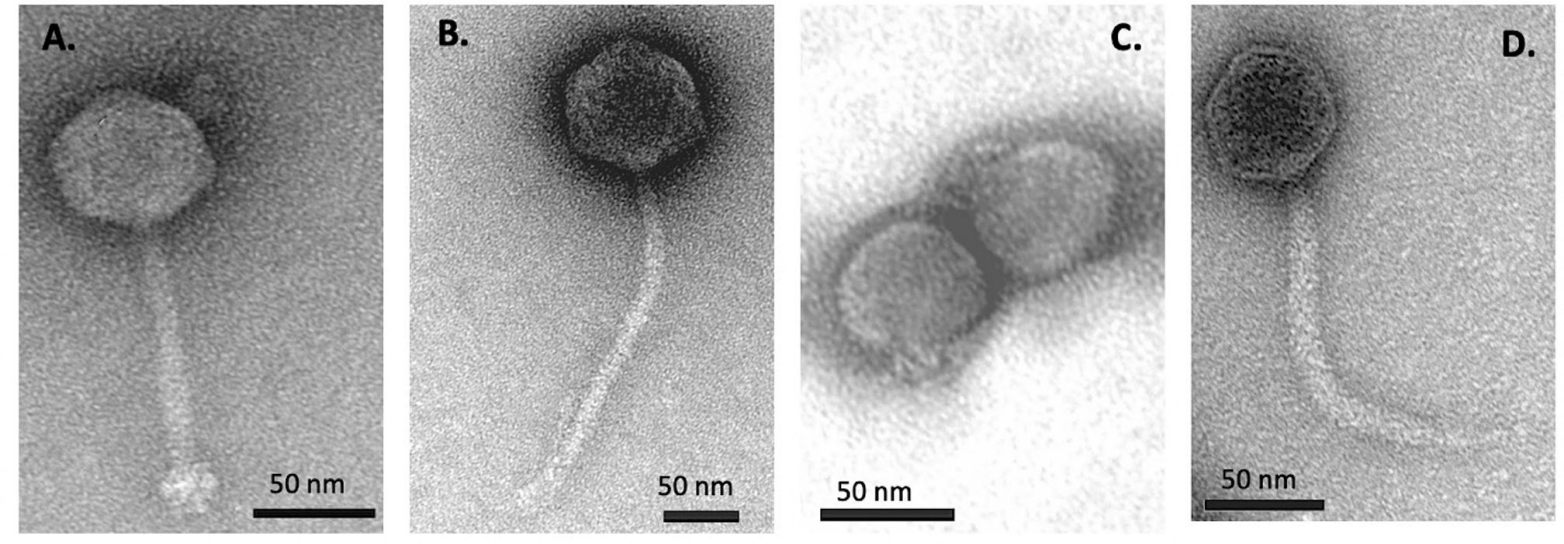
Morphological features of selected phage members of the four MTs. (A) MT1 siphophage K6 with capsid diameter of approx. 50 nm and tail of 100 nm. (B) MT2 siphophage K18 with capsid diameter of 75 nm and tail of 200 nm. (C) MT3 podophage K30, with capsid diameter of 50 nm. (D) MT4 siphophage K19, with capsid diameter of 50 nm and tail of 125 nm.

To confirm the morphology of these phages, 17 selected phage isolates were observed under a transmission electron microscope (TEM). Bacterial viruses belonging to MT1, MT2, and MT4 were found to be siphophages, while those of MT3 cluster were podophages. There were notable structural differences (capsid diameter, tail length, baseplates, and tail fibers) among siphophages from different MTs, but no differences were observed among STs within one MT. The morphology of a selected member of each of the four MTs is shown in Fig. 7. Taken together, the phage morphology suggested by the genome analyses was confirmed by TEM observations.

### Host range

As some of these phages may be used in phage cocktails to control *Salmonella* in the poultry industry in Kenya, their host range was determined. A set of 16 *Salmonella* strains isolated from Kenyan chickens and slaughterhouses were selected for the host range assay (Table 4). These strains covered three serotypes, namely Enteritidis, Kentucky, and Heidelberg. Based on the genomic and taxonomic analyses above, we also added *Salmonella* strains from other geographic locations as well as strains of *E. coli*, *Shigella*, *Citrobacter*, *Enterobacter*, and *Kluyvera*, which all belong to the *Enterobacteriaceae* family (order Enterobacteriales). We also included a few strains from distantly related genera from the Enterobacteriales order, namely *Hafnia* and *Erwinia*. The host ranges of the 17 reference phages on the Kenya *Salmonella* strains and other related bacterial hosts are summarized in Tables 3 and 4. The host ranges of the 59 isolates are provided in Table S15.

Overall, phages within MT1 had a similar host range on the *Salmonella* strains isolated from Kenya (Table 4) and on other *Salmonella* strains and related bacterial hosts (Table 3). The host ranges of the phages belonging to the MT2 cluster were also similar in the Kenyan *Salmonella* strains with the notable exception of phage K37 (MT2, ST2-6) which infected three more Kenyan hosts than the rest of the MT2 phages (Table 4). However, significant differences were observed in the host ranges of the various STs within MT2 on the other *Salmonella* and related host species (Table 3). Interestingly, all MT2 phages infected *E. coli* strains as well as some *Shigella* strains. Some MT2-STs even infected strains of *E. coli* O157:H7. These differences in the host ranges of the STs in the MT2 cluster are likely due to amino acid variations observed in the receptor-binding proteins such as tail spike proteins (Fig. 4). As mentioned previously, phage K30 within the MT3 group had the broadest host range against the *Salmonella* strains from Kenya and against other serovars as well (Table 3). The only phage in MT4 group also infected *E. coli* and *Shigella* strains in addition to very few *Salmonella* strains. Of note, the Kenyan phage set isolated in this study could kill all the 16 *Salmonella* strains from Kenya.

### Replication and packaging mechanisms of MTs

**Fig 8:**
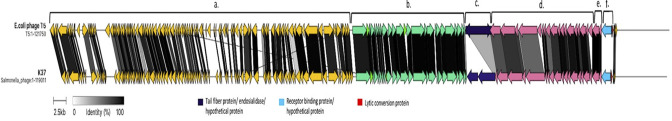
Alignment of deduced proteins between Salmonella phage K37 (MT2) and Escherichia phage T5. (A) Genomic region coding for proteins with unknown or other functions (orange), (B) Proteins involved in replication (green), (C) Tail fiber proteins or endosialidase in phage K37 and hypothetical protein in T5, (D) Phage structural proteins (pink), (E) Phage proteins involved in packaging (pink), and (F) Receptor binding and lytic conversion proteins of phage T5 and a hypothetical and lytic conversion proteins in phage K37.

**Fig 9:**
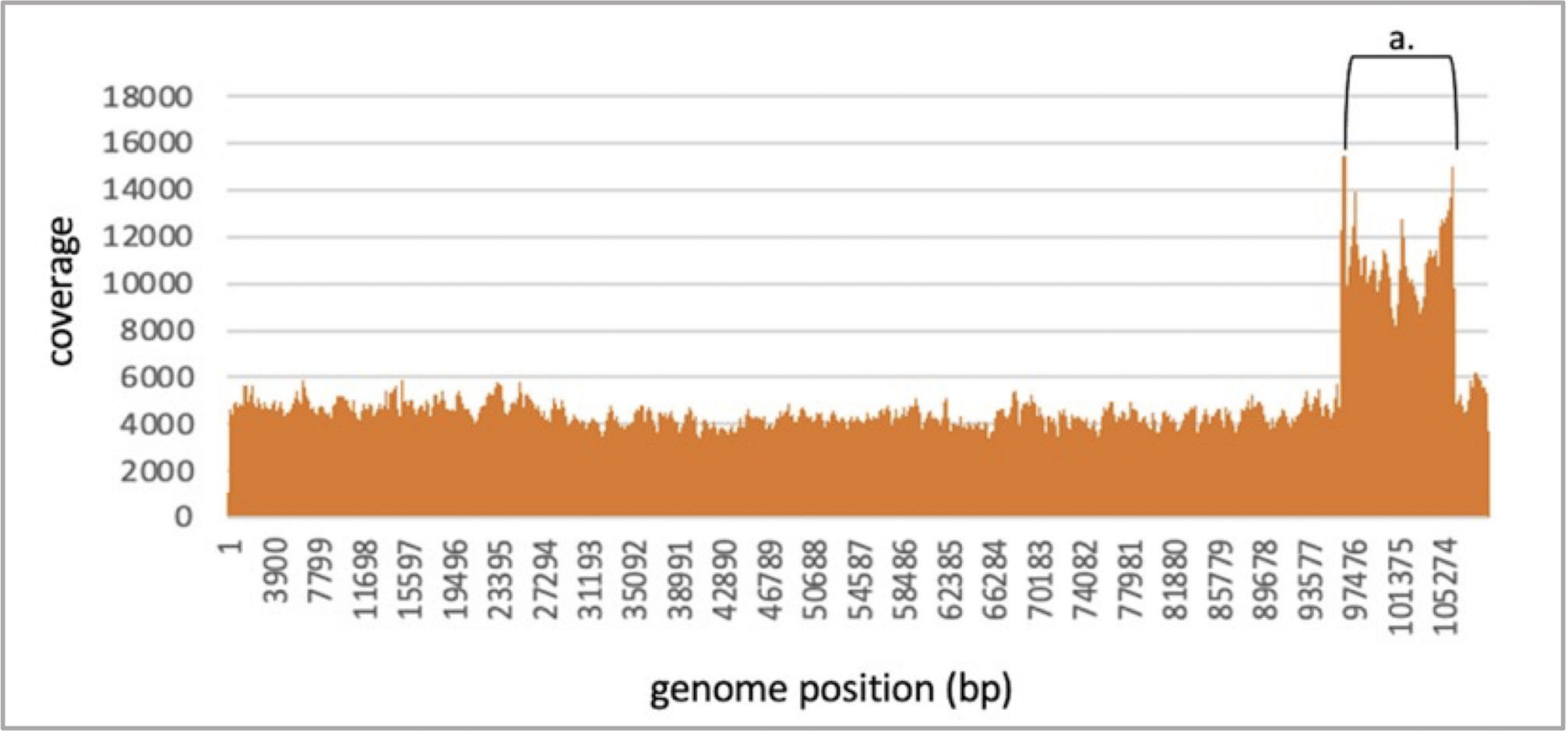
Genome coverage at each base position of the phage K37 genome after Illumina MiSeq sequencing. a. Region indicating high coverage due to the overlapping direct LTRs.

**Fig 10:**
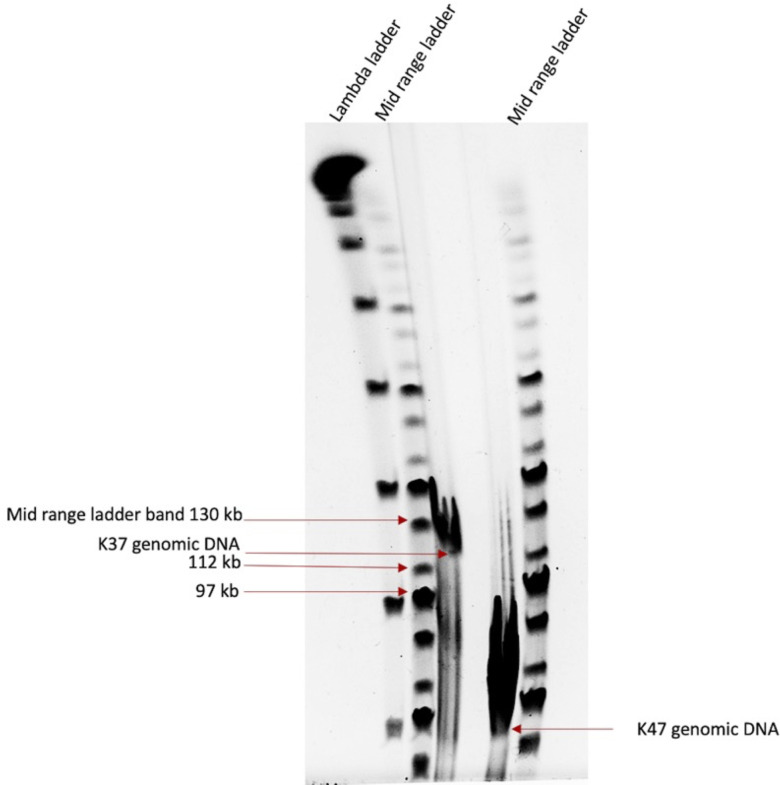
Pulsed-field gel electrophoresis (PFGE) of undigested genomic DNA from phage K47 (MT1) and phage K37 (MT2).

As indicated above, phages belonging to the MT2 cluster were classified into the T5-like group, namely the *Tequintavirus* genus. In fact, the MT2 genomes/proteomes are very similar to that of Escherichia phage T5, including the replication and the structural modules as well as the packaging proteins (Fig. 3 and 8). For example, the DNA polymerases of MT2 phages are 99% similar to that of T5. It has been shown previously that the phage replication and packaging mechanisms can be predicted by observing the phylogenetic relationships of DNA polymerase and terminase large subunit protein sequences (49, 50). Hence, we predicted that the MT2 phages have a recombination-based replication strategy as in phage T5 (51). The terminase large subunit protein of T5 is identical to MT2 terminases. Phage T5 has a long terminal repeat (LTR)-based packaging mechanism due to the presence of 10,219 bp LTRs (52). PhageTerm analysis predicted that the MT2 phages also have T5-like LTRs. Examination of the Illumina MiSeq coverage maps revealed that each MT2 genome had a high coverage region, which was more than twice the rest of the genome (Fig. 9). This is an evidence by the presence of two direct terminal repeats which usually overlap during a read assembly to produce a single high coverage segment in a genome assembled into a circular topology (53). Experimental confirmation of the LTRs was established by migrating phage K37 (MT2) genomic DNA by Pulsed-Field Gel Electrophoresis (PFGE). The K37 genome size estimation by PFGE showed that the actual genome size is larger than the ~109 kbp sequence length of the contig assembly (Fig. 10). That is because the actual genome should be ~119 kbp long with the presence of LTRs. All these evidences point to the fact that MT2 phages use an LTR-based packaging mechanism, as in phage T5.

**Fig 11:**
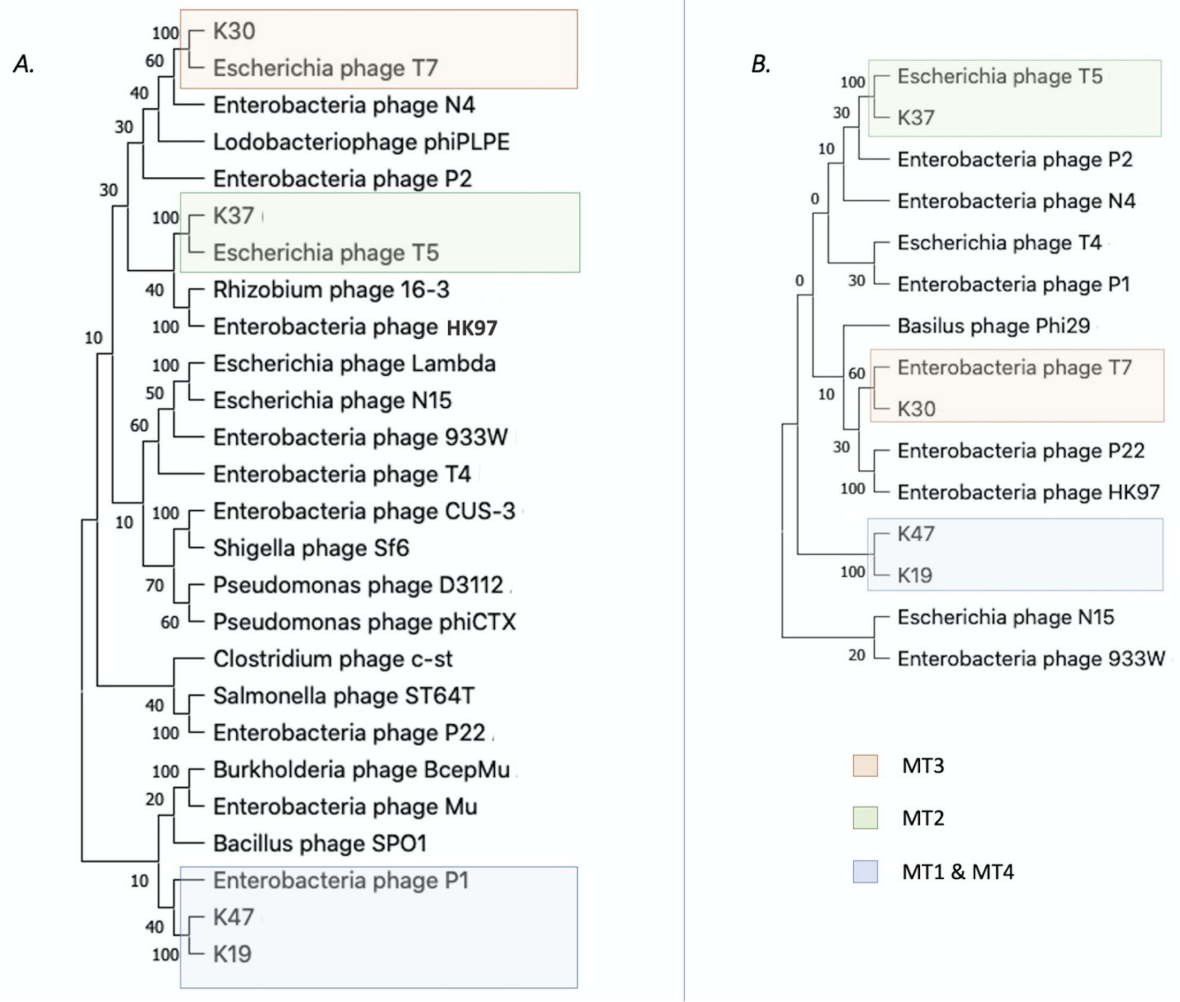
Phylogenetic trees with the terminase large subunits and phage DNA polymerases. (A) Bootstrapped maximum likelihood tree generated with terminase large subunit protein sequences of four Kenyan phages representing the four MTs and other terminases from phages with previously characterized packaging mechanisms. (B) Bootstrapped maximum likelihood tree generated with DNA polymerase protein sequences of four Kenyan phages representing the four MTs and other DNA polymerases from phages with previously characterized replication mechanisms.

The DNA polymerases of MT1 phages showed a phylogenetic relationship with its counterpart from MT4 (K19), but it did not show a notable phylogenetic relationship to any other known DNA polymerases (Fig. 11B). The MT1 terminase large subunits, however, had a phylogenetic relationship to its MT4 counterpart (K19), as well as to Enterobacteria phage P1, which has a headful packaging mechanism and the initiation of packaging occurs at a *pac* site (52). Upon further analysis, PhageTerm bioinformatic analysis of the phage termini of MT1 (K47) also concluded that it has a P1-like headful packaging mechanism. A restriction profile of a headful packaging phage usually resembles a simulated profile if the genome is circular. When there is headful packaging with *pac* initiation, it could be a restriction profile with all bands from a circular profile plus a submolar *pac* fragment (50). However, as shown previously (54), the EcoRV restriction profile of K47 resembled the circular profile (Fig. S4). Another feature of headful packaging mechanism is that they tend to produce a wider band when undigested genomic phage DNA is migrated on PFGE as observed in Fig. 10. This is in contrast to the concise bands that are produced by non-headful packaging phages (50, 55). Taken together, MT1 phages have headful packaging mechanisms, likely with a *pac* site-based packaging initiation.

Because the DNA polymerase and terminase large subunit of the podophage K30 (MT3) showed phylogenetic relationships to those proteins of coliphage T7, MT3 phages may have a T7-like replication and packaging mechanism. The T7 replication mechanism has been identified as replication initiated by transcription or tDR (51) and uses its 200 bp terminal repeats for packaging (52). However, K30 DNA polymerase showed only partial similarity to T7 DNA polymerase, with 34.3% amino acid identity (30% query cover). On the other hand, K30 terminase large subunit (TerL) showed 35.9% amino acid identity (83% query cover) to T7 TerL, and it consists of a conserved domain that belongs to Termin_lrg_T7 protein superfamily. However, other than the single protein phylogenetic relationships to T7, we were unable to produce any other evidence or experimental data to support the T7-like replication and packaging for MT3 phages. The MT4 and MT1 (K47) DNA polymerases share 65.2% amino acid identity (99% query cover). TerLs of MT4 (K19) and MT1 (K47) have 41.4% shared amino acid identity (94% query cove). Hence, the replication mechanism of MT4 may be similar to that of MT1 (Fig. 11B). Yet, we were unable to support these claims with any other bioinformatic or experimental evidence.

### Conclusion

All the phages isolated from samples collected in Kenyan chicken farms and slaughterhouses belong to four major types of phages, with each MT representing a phage genus. MT1 and MT2 consisted of multiple phage genomes that could be clustered into subtypes. Siphophages were grouped in MT1, MT2, or MT4, while podophages were in MT3. MT1 phages belong to the *Jerseyvirus* genus with a headful packaging mechanism with *pac* initiation. MT2 phages are T5-like viruses in the genus *Tequintavirus*. MT3 phages belong to the *Zindervirus* genus and may have T7-like replication and packaging. Phage K19 (MT4) was unique in our *Salmonella* phage set, but it belongs to the *Dhillonvirus* genus, for which no *Salmonella* phages had been included yet. Host range assays indicated that MT1 and MT3 phages could infect only *Salmonella* strains, while MT2 and MT4 phages could also infect *Salmonella*, *E. coli*, and *Shigella* strains. It remains to be seen if these phages can be used in combination with each other and/or with other characterized phages to control diverse *Salmonella* strains in poultry applications.

## MATERIALS AND METHODS

### Sample collection, *Salmonella enterica* hosts, and phage isolation

To isolate the hosts and phages, 631 samples were collected from 61 chicken farms and 4 slaughterhouses in Kenya. Specifically, 138 samples of chicken droppings, 53 samples from various water sources and 43 environmental swabs were collected from the county of Nairobi. Similarly, 321 samples of chicken droppings, 61 samples from various water sources, and 15 environmental swabs were collected from Kiambu County.

### *Salmonella* strains isolation and identification

To isolate *Salmonella*, 1 g from each chicken fecal sample and 1 mL from each water sample were inoculated separately into 10 mL of Tryptic-soy broth medium (TSB; Oxoid, Hampshire, UK) and incubated at 37°C for 16–18 h. Then, 0.1 mL of these cultures were transferred to 10 mL of Rappaport–Vassiliadis (RV) broth (Oxoid) and incubated at 37°C for 24 h. On the following day, a loop of inoculum from RV broth was plated onto various media including MacConkey, Salmonella-Shigella, Brilliance Salmonella, and Xylose lysine Tergitol 4 to isolate pure colonies of suspected *Salmonella* strains. After another 24–48 h incubation at 37°C, the colonies that displayed characteristics of *Salmonella* were re-streaked and selected from each plate for preliminary identification using an API-20E test kit (bioMérieux, Marcy l’Etoile, France).

The serological identification of the suspected *Salmonella* isolates was done by slide agglutination using Polyvalent O and H *Salmonella* antisera (*Salmonella* Agglutinating Serum, Remel Europe Ltd, Cambridge, UK), according to the White–Kauffmann–LeMinor scheme (52). The *Salmonella* serotypes were confirmed by the analyses of the two CRISPR loci (CRISPR-1 and CRISPR-2) (56, 57). CRISPR loci were amplified by PCR using 1 µL of 10^-2^ diluted overnight *Salmonella* culture as the template. Primers are listed in Table 5. PCR products were sequenced at the Plateforme de séquençage Sanger du Centre de recherche du CHU de Québec-Université Laval. CRISPR sequences were compared to serotype-specific CRISPR loci using NCBI BLASTn.

**TABLE 5:** Primer sets for CRISPR typing

Primer name	CRISPR locus	Orientation	Sequence (5′−3′)	Ref.
CRISPR1-5	1	Fwd	TGAAAACAGACGTATTCCGGTAGATT	(48)
CRISPR1-1		Rev	CAGCATATTGACAAGGCGCT	
A1		Fwd	GTRGTRCGGATAATGCTGCC	(49)
A2		Rev	CGTATTCCGGTAGATBTDGATGG	
CRISPR2-3	2	Fwd	ATTGTTGCGATTATGTTGGT	(48)
CRISPR2-1		Rev	GATCCTTAACGCCATGGCCT	
B1		Fwd	GAGCAATACYYTRATCGTTAACGCC	(49)
B3		Rev	CTGGCGGCTGTCTATGCAAAC	

### Isolation of *Salmonella* phages

To isolate *Salmonella* phages, 1 g from each chicken fecal sample and 1 mL from each water sample were inoculated separately into 10 mL of TSB. After incubating at 42°C for 24 h, the cultures were centrifuged 4,000 × *g* for 15 min at room temperature and the supernatants were filtered through 0.22 µm PEP syringe filters. Using the agar overlay method, 4 µL of the filtrate were spotted on TSA 1% (wt/vol) agar plate (Thermo Fisher Scientific, UK) with TSA 0.5% (wt/vol) agar as the top layer, which was mixed with 100 µL of exponentially growing *Salmonella* host culture. Plates were incubated at 37°C for 18 h and examined for phage plaques.

For phage purification, individual plaques were picked with a sterile loop and mixed with 500 µL of SM buffer [100 mM NaCl, 8 mM MgSO_4_, 50 mM Tris–HCl and 0.01% (wt/vol) gelatin]. Phages were let to diffuse in the buffer for 12 h at 4°C and then centrifuged at 21,000 × *g* for 10 min. The resulting supernatant was used for the second round of phage purification by the agar overlay method. The plaque purification process was repeated for a total of five times. Phage lysates were produced by mixing exponentially growing liquid cultures of host strains (OD_600nm_=0.2–0.5) in TSB broth with phage preparations (titer of 10^7^ PFU/mL) and incubated at 37°C with shaking (200 rpm) for 5 h or until the culture was clear. These phage lysates were centrifuged at 4,200 × *g* for 15 min and the supernatant was filtered (0.22 µm). Filtrates were stored at 4°C until use or frozen at −80°C with 15% glycerol. To determine the phage titer, each phage lysate was serially diluted (10-fold), and plaque assays were conducted using the agar overlay method.

### Phage DNA extraction, sequencing, and sequence analyses

Phage genomic DNA was purified from high-titer lysates using phenol-chloroform extraction (58). The library for next-generation sequencing was prepared with a Nextera XT DNA Sample Preparation kit (Illumina, San Diego, CA, USA). Paired-end reads (250 bp) were generated using the Illumina MiSeq Platform with Reagent Kit v2. Once the reads were produced, FastQC (59) was used to evaluate the read quality. Prior to assembly, the Illumina adapters were removed and reads were trimmed using Trimmomatic v0.39 (60). The assembly was performed using Spades assembler v3.13.0 (61) with the default parameters. The reads were also assembled with Ray v2.3.2 to compare and verify the accuracy of each assembly (62). The genome sequences were subjected to pairwise analysis with JSpeciesWS (28) and mash v2.3.4 (63) to identify similar genomes and to cluster them according to their nucleotide identities. These clusters were visualized using seaborn (29). The SNP (Single Nucleotide Polymorphisms) and small insertion/deletion variations among highly similar genomes (Tables S3 to S12) were determined with Geneious R11 (64). From each group of similar phages, one sequence was used as the reference genome for further analyses. NCBI BLASTn (65) was used to determine whether similar phage genomes were already present in public databases.

The gene prediction and functional annotation of the reference genomes were performed using RAST (Rapid Annotation using Subsystem Technology) v2.0 (66) in combination with NCBI domain searches (67) and BLASTp (65). The presence of virulence factors in phage genomes was checked against the virulence factor database, VFDB (68). Genome visualizations and GenBank submissions were carried out using Geneious R11 (64). The nucleotide alignments of the reference genomes were performed with Mauve alignment tool (69). The protein similarity illustrations were drawn with the Clinker protein alignment visualization tool (70). Phylogenetic analyses were performed with MEGA 11.0 (71). The taxonomic classification of phages was determined with vConTACT2 0.9.19 (72), available at KBase (https://www.kbase.us/). This tool clustered the Kenyan phage genomes using an inbuilt viral database with a protein cluster value range of 0.75–1 and a viral cluster value of 0.9–1. The program then generated phage genome network files that were visualized and edited with Cytoscape 3.9.1 (73). Bioinformatic determination of phage termini and packaging mechanisms were carried out using PhageTerm (74). To confirm phage genome termini, restriction mapping and pulsed-field gel electrophoresis (PFGE) were performed. The PFGE was performed as described previously (75) with the exception that the undigested genomic DNAs were directly loaded onto the gel after mixing with a gel loading dye (NEB). The electrophoresis was performed with 10 V/cm for 24 h.

### Host range determination

The host range of each phage isolate was determined by spotting undiluted and diluted phage lysates on lawns of Kenyan *Salmonella* strains isolated in this study. The phage lysates (10^9^–10^10^ PFU/mL) were serially diluted into 10-fold dilutions and the host bacterial lawns were prepared by mixing 200 µL of the overnight host culture with 7 mL of soft agar (0.75% wt/vol in TSA) as the top layer. From each undiluted and diluted phage lysate, 2 µL was spotted on the bacterial lawn and incubated at 37°C for 18 h. A clear zone or plaque formation was recorded as a positive phage infection. This experiment was done in two independent replicates. For each phage lysate, the phage titer on each host was also determined using plaque assays.

The host range of selected phage isolates was also tested on other strains of *Salmonella* and related bacterial species. For this experiment, we included eight non-typhoidal *Salmonella* strains reportedly isolated from North America, three strains of Typhoidal *Salmonella*, eight strains of *E. coli*, four strains of *Shigella*, two strains of *Citrobacter freundii*, two strains of *Erwinia* and one strain from each *Enterobacter cloacae*, *Hafnia alvei*, and *Kluyvera cryocrescens*. Positive phage infections on these hosts were recorded as above. This experiment was also performed in two replicates and plaque assays were used to calculate the phage titers on each host.

### Transmission electron microscopy

About 1 mL of phage lysate (10^9^–10^10^ PFU/mL) was pelleted by centrifugation at 24,000 × *g* for 1 h at 4°C. The supernatant was gently discarded, and the pellet was washed by adding 1 mL of sterile distilled water and centrifuging again. This step was repeated a second time. The supernatant was removed, and the pellet was suspended in 10 µL of sterile distilled water. For the grid preparation, 10 µL of 2% uranyl acetate was placed on a 200-mesh Formvar/carbon-coated copper grid (Pelco International, Redding, CA, USA) for 30 s. Then, 10 µL of phage suspension were mixed with the stain on the grid by gently pipetting up and down. The mixture was left on the grid for another 30 s. The excessive liquid on the grid was blotted using a Whatman paper. A thin layer of uranyl acetate was left on the grid by avoiding a complete blotting. The grids were air-dried and observed with a JEM-1230 transmission electron microscope (JEOL, Tokyo, Japan). Images were captured using the Gatan Digital Micrograph software (version 1.3) after 10 s exposure (76).

## SUPPLEMENTAL MATERIAL

Fig. S1 spectrum.02729-23-s0001.tiff Distance matrix of the 59 phage isolates showing 4 different major types and possible subtypes based on their genomic similarities after pairwise analyses.

Fig. S2 spectrum.02729-23-s0002.tiff Mauve alignments of phage genomes.

Fig. S3 spectrum.02729-23-s0003.tiff Proteome comparison of 4 reference phages from 4 MTs (K47 from MT1, K32 from MT2, K30 from MT3, and K19 from MT4) with their top BLASTn hits from NCBI.

Fig. S4 spectrum.02729-23-s0004.tiff Simulated EcoRV restriction profiles of phage K47 linear and circular genome by Geneious software.

Supplemental tables spectrum.02729-23-s0005.docx Tables S1 to S15; legends for Fig. S1 to S4.

ASM does not own the copyrights to Supplemental Material that may be linked to, or accessed through, an article. The authors have granted ASM a non-exclusive, world-wide license to publish the Supplemental Material files. Please contact the corresponding author directly for reuse.
