# Supplementary figures and images for: Diversity of Salmonella enterica phages isolated from chicken farms in Kenya
Source: Microbiol Spectr. 2023 Dec 11;12(1):e02729-23. doi: 10.1128/spectrum.02729-23 (PMC10783031; doi:10.1128/spectrum.02729-23)

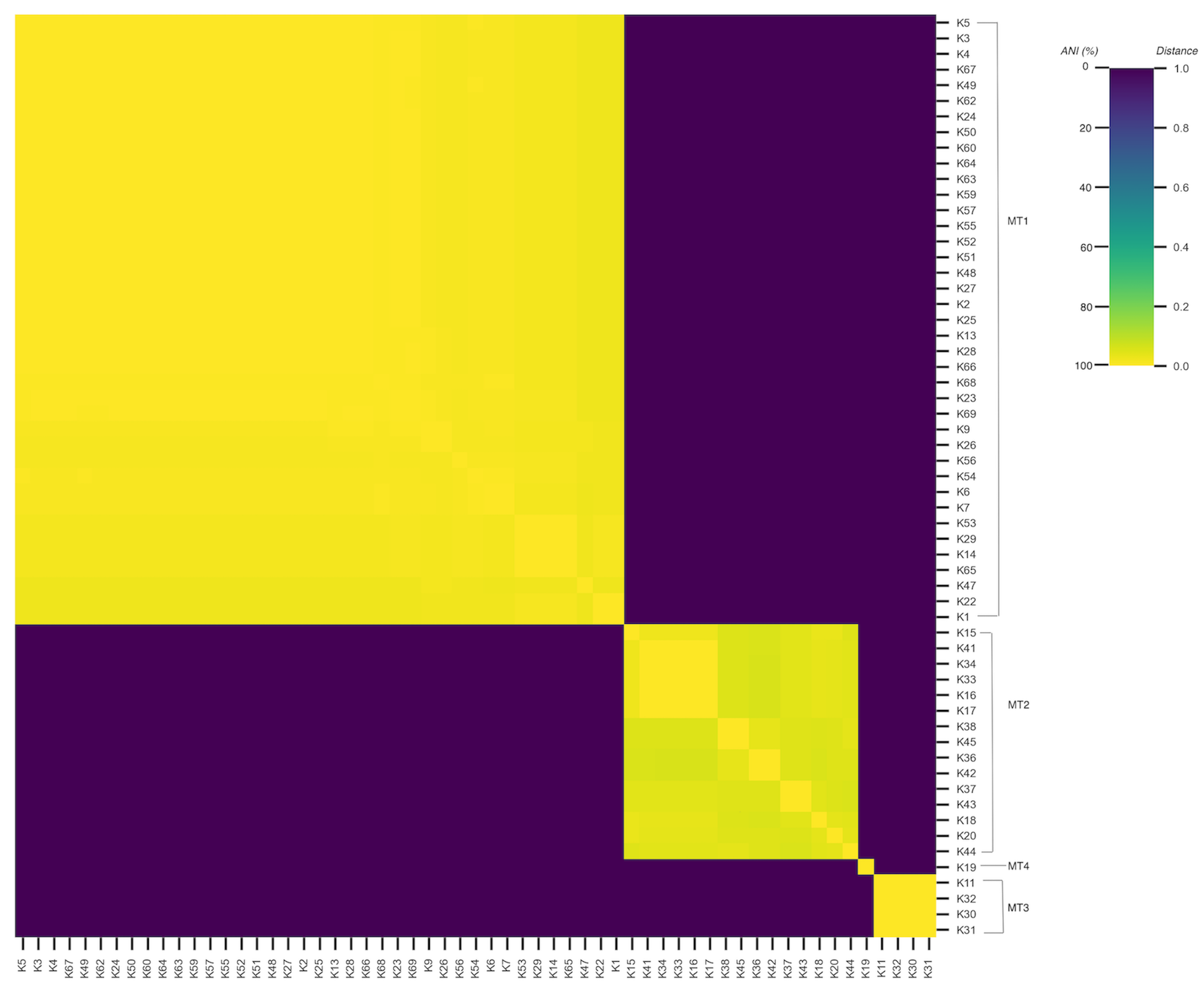

Supplement: Fig. S1 — Distance matrix of the 59 phage isolates showing 4 different major types and possible subtypes based on their genomic similarities after pairwise analyses. [file spectrum.02729-23-s0001.tiff]

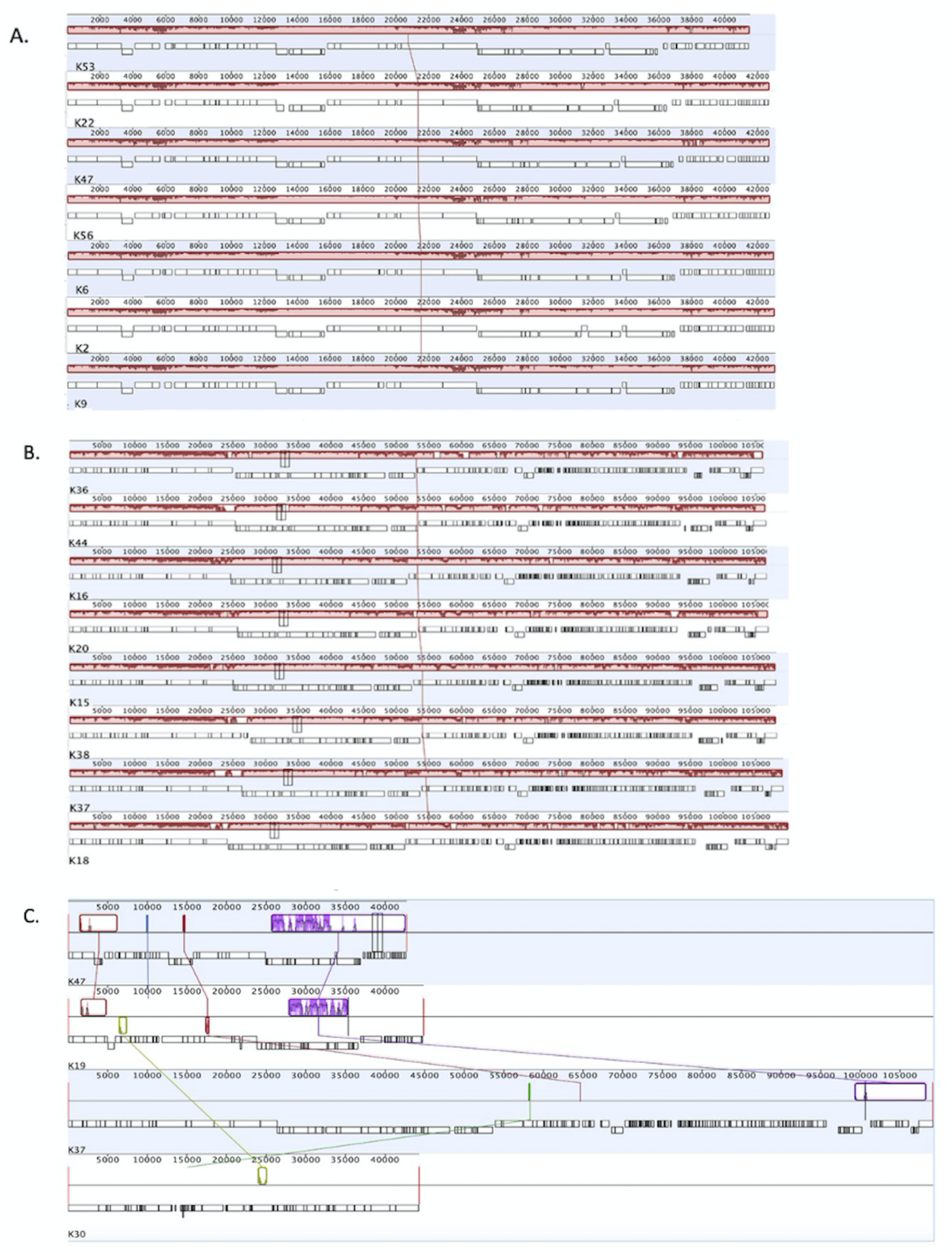

Supplement: Fig. S2 — Mauve alignments of phage genomes. [file spectrum.02729-23-s0002.tiff]

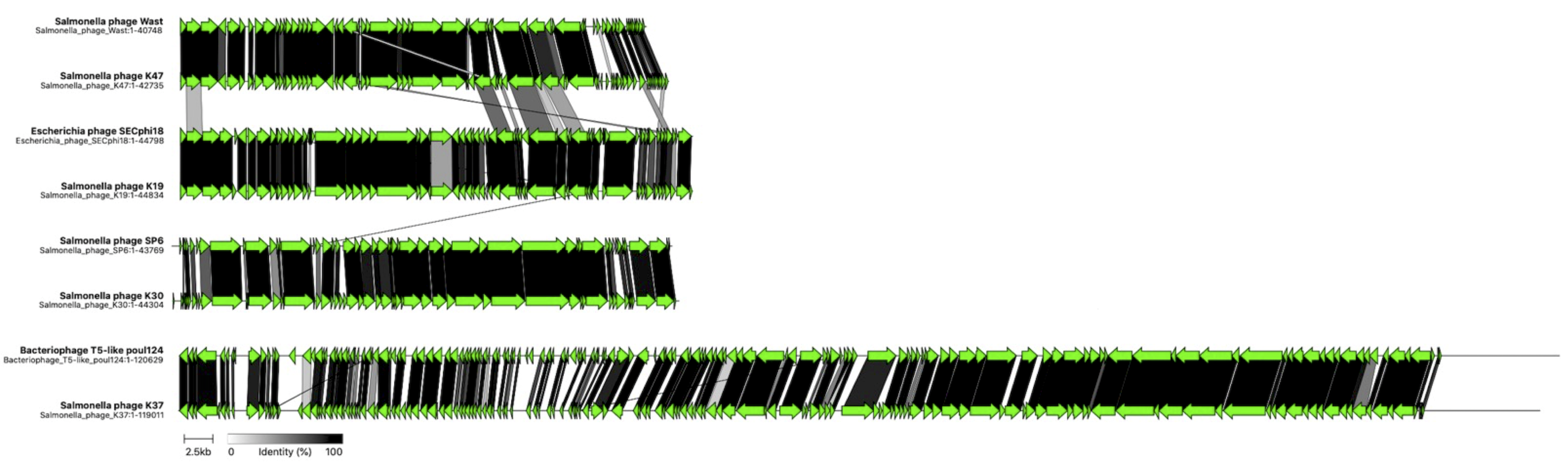

Supplement: Fig. S3 — Proteome comparison of 4 reference phages from 4 MTs (K47 from MT1, K32 from MT2, K30 from MT3, and K19 from MT4) with their top BLASTn hits from NCBI. [file spectrum.02729-23-s0003.tiff]

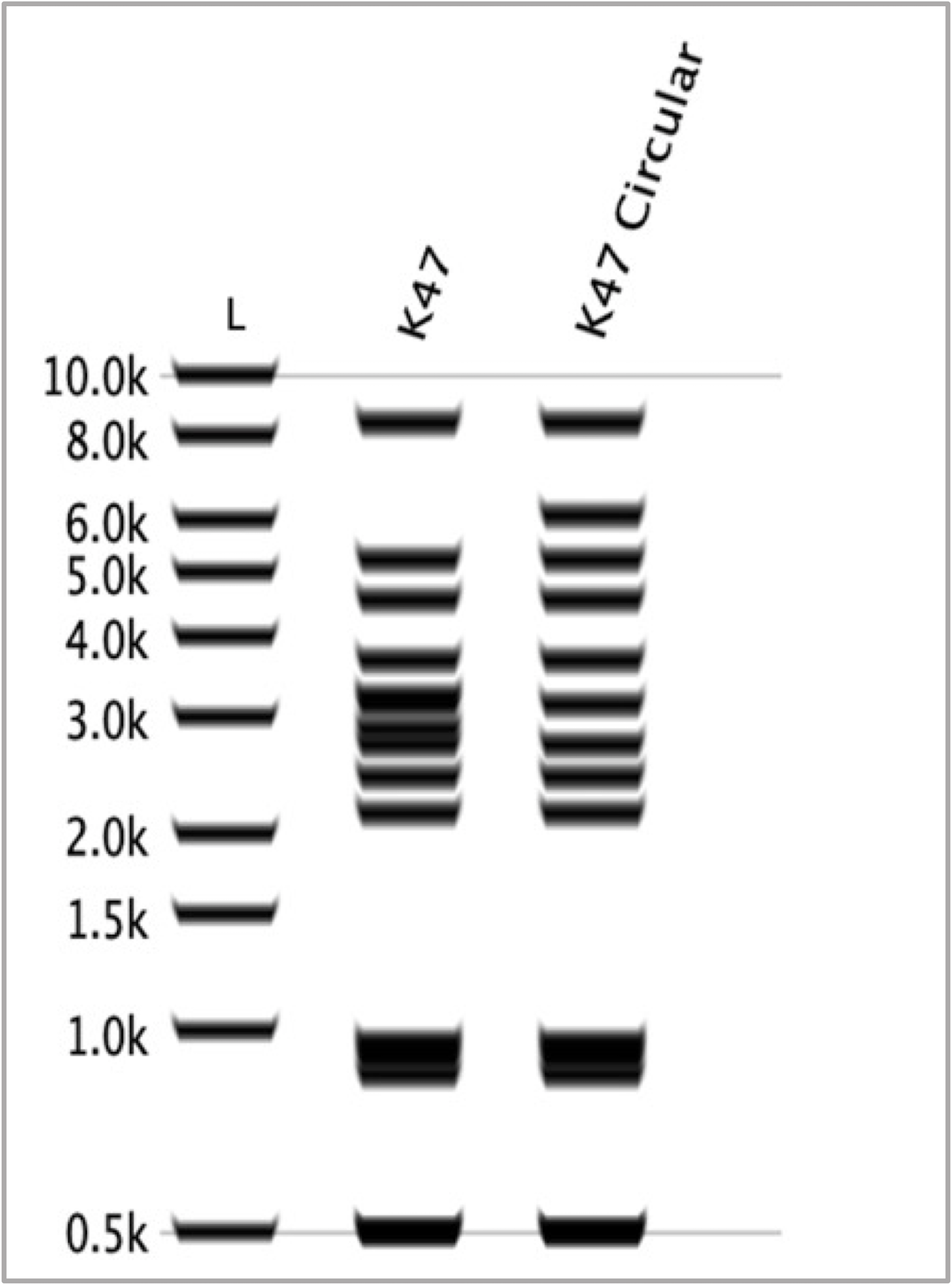

Supplement: Fig. S4 — Simulated EcoRV restriction profiles of phage K47 linear and circular genome by Geneious software. [file spectrum.02729-23-s0004.tiff]
