# Supplementary material for: Diversity of Salmonella enterica phages isolated from chicken farms in Kenya
Source: Microbiol Spectr. 2023 Dec 11;12(1):e02729-23. doi: 10.1128/spectrum.02729-23 (PMC10783031; doi:10.1128/spectrum.02729-23)
Supplement: Supplemental tables — Tables S1 to S15; legends for Fig. S1 to S4. [file spectrum.02729-23-s0005.docx]

Supplement Table 1: GenBank accession numbers and sample metadata of the phage genome sequences other than the 17 reference genomes.

| Phage isolate | Host (Kenyan *Salmonella* isolate) | GenBank  accession number | Sample metadata | | |
| --- | --- | --- | --- | --- | --- |
|  |  |  | Type of sample (droppings/water/  chicken surface swabs) | Location | |
|  |  |  |  | City | Farm (PF)/  Slaughterhouse number (SH) |
| Salmonella_phage_Kenya-K1 | Sal16 | OQ291037 | Droppings | Kiambu | PF17 |
| Salmonella_phage_Kenya-K3 | Sal16 | OQ291038 | Water | Nairobi | SH2 |
| Salmonella_phage_Kenya-K4 | Sal16 | OQ291039 | Water | Nairobi | SH2 |
| Salmonella_phage_Kenya-K5 | Sal16 | OQ291040 | Water | Nairobi | SH2 |
| Salmonella_phage_Kenya-K7 | Sal16 | OQ291041 | Water | Nairobi | SH3 |
| Salmonella_phage_Kenya-K11 | Sal73 | OQ291042 | Water | Kiambu | SH1 |
| Salmonella_phage_Kenya-K13 | Sal73 | OQ291043 | Water | Nairobi | SH2 |
| Salmonella_phage_Kenya-K14 | Sal73 | OQ291044 | Droppings | Kiambu | PF54 |
| Salmonella_phage_Kenya-K17 | Sal157 | OQ291045 | Water | Kiambu | SH1 |
| Salmonella_phage_Kenya-K23 | Sal177 | OQ291046 | Water | Kiambu | SH1 |
| Salmonella_phage_Kenya-K24 | Sal177 | OQ291047 | Water | Nairobi | SH2 |
| Salmonella_phage_Kenya-K25 | Sal177 | OQ291048 | Water | Nairobi | SH2 |
| Salmonella_phage_Kenya-K26_contigs 1 and 2 | Sal177 | OQ291049 OQ291050 | Swab | Nairobi | SH2 |
| Salmonella_phage_Kenya-K27 | Sal177 | OQ291051 | Water | Nairobi | SP3 |
| Salmonella_phage_Kenya-K28 | Sal177 | OQ291052 | Water | Nairobi | SP3 |
| Salmonella_phage_Kenya-K29 | Sal177 | OQ291053 | Droppings | Kiambu | PF54 |
| Salmonella_phage_Kenya-K31 | Sal187 | OQ291054 | Water | Kiambu | SH1 |
| Salmonella_phage_Kenya-K32 | Sal188 | OQ291055 | Water | Kiambu | SH1 |
| Salmonella_phage_Kenya-K33 | Sa192 | OQ291056 | water | Kiambu | SH1 |
| Salmonella_phage_Kenya-K34 | Sal192 | OQ291057 | Water | Kiambu | SH1 |
| Salmonella_phage_Kenya-K41 | Sal194 | OQ291058 | Water | Kiambu | SH1 |
| Salmonella_phage_Kenya-K42 | Sal194 | OQ291059 | Water | Nairobi | PF38 |
| Salmonella_phage_Kenya-K43 | Sal194 | OQ291060 | Droppings | Nairobi | PF41 |
| Salmonella_phage_Kenya-K45 | Sal194 | OQ291061 | Droppings | Kiambu | PF50 |
| Salmonella_phage_Kenya-K48 | Sal568 | OQ291062 | Droppings | Kiambu | PF1 |
| Salmonella_phage_Kenya-K49 | Sal568 | OQ291063 | Droppings | Kiambu | PF17 |
| Salmonella_phage_Kenya-K50 | Sal568 | OQ291064 | Water | Nairobi | SH2 |
| Salmonella_phage_Kenya-K51 | Sal568 | OQ291065 | Water | Nairobi | SH2 |
| Salmonella_phage_Kenya-K52 | Sal568 | OQ291066 | Water | Nairobi | SH3 |
| Salmonella_phage_Kenya-K54 | Sal569 | OQ291067 | Water | Nairobi | PF44 |
| Salmonella_phage_Kenya-K55 | Sal569 | OQ291068 | Droppings | Kiambu | PF54 |
| Salmonella_phage_Kenya-K57 | Sal572 | OQ291069 | Droppings | Kiambu | PF17 |
| Salmonella_phage_Kenya-K59 | Sal572 | OQ291070 | Water | Nairobi | SH2 |
| Salmonella_phage_Kenya-K60 | Sal572 | OQ291071 | Water | Nairobi | SH3 |
| Salmonella_phage_Kenya-K62 | Sal572 | OQ291072 | Water | Nairobi | SH3 |
| Salmonella_phage_Kenya-K63 | Sal572 | OQ291073 | Droppings | Kiambu | PF54 |
| Salmonella_phage_Kenya-K64 | Sal572 | OQ291074 | Water | Nairobi | SH3 |
| Salmonella_phage_Kenya-K65 | Sal572 | OQ291075 | Droppings | Kiambu | PF54 |
| Salmonella_phage_Kenya-K66 | Sal177 | OQ291076 | Water | Nairobi | SH2 |
| Salmonella_phage_Kenya-K67 | Sal568 | OQ291077 | Droppings | Kiambu | PF17 |
| Salmonella_phage_Kenya-K68 | Sal569 | OQ291078 | Water | Nairobi | SH2 |
| Salmonella_phage_Kenya-K69 | Sal572 | OQ291079 | Water | Nairobi | SH3 |
| ***Salmonella_phage_Kenya-K2** | **Sal16** | **OQ291020** | **Water** | **Nariobi** | **SH2** |
| **Salmonella_phage_Kenya-K6** | **Sal16** | **OQ291021** | **Water** | **Nairobi** | **SH3** |
| **Salmonella_phage_Kenya-K9** | **Sal16** | **OQ291022** | **Droppings** | **Nairobi** | **PF44** |
| **Salmonella_phage_Kenya-K15** | **Sal157** | **OQ291029** | **Water** | **Kiambu** | **SH1** |
| **Salmonella_phage_Kenya-K16** | **Sal157** | **OQ291030** | **Water** | **Kiambu** | **SH1** |
| **Salmonella_phage_Kenya-K18** | **Sal157** | **OQ291031** | **Water** | **Nairobi** | **PF43** |
| **Salmonella_phage_Kenya-K19** | **Sal157** | **OQ291023** | **Water** | **Nairobi** | **SH2** |
| **Salmonella_phage_Kenya-K20** | **Sal157** | **OQ291032** | **Droppings** | **Nairobi** | **SH46** |
| **Salmonella_phage_Kenya-K22** | **Sal177** | **OQ291024** | **Droppings** | **Kiambu** | **PF17** |
| **Salmonella_phage_Kenya-K30** | **Sal187** | **OQ291025** | **Water** | **Kiambu** | **SH1** |
| **Salmonella_phage_Kenya-K36** | **Sal192** | **OQ291033** | **Water** | **Nairobi** | **PF38** |
| **Salmonella_phage_Kenya-K37** | **Sal192** | **OQ291034** | **Droppings** | **Nairobi** | **PF41** |
| **Salmonella_phage_Kenya-K38** | **Sal192** | **OQ291035** | **Droppings** | **Kiambu** | **SP54** |
| **Salmonella_phage_Kenya-K44** | **Sal194** | **OQ291036** | **Water** | **Nairobi** | **SH2** |
| **Salmonella_phage_Kenya-K47** | **Sal312** | **OQ291026** | **Water** | **Nairobi** | **SH2** |
| **Salmonella_phage_Kenya-K53** | **Sal569** | **OQ291027** | **Water** | **Nairobi** | **SH2** |
| **Salmonella_phage_Kenya-K56** | **Sal572** | **OQ291028** | **Droppings** | **Kiambu** | **PF17** |

* The bolded and underlined phage names belong to the reference phage isolates described in the main text. Meaning in text PF: Poultry farm while SH: Slaughterhouse.

Supplement Table 2: Sample metadata of the 16 *Salmonella enterica* isolates from Kenya.

| Serotype | *Salmonella enterica* isolate |  | Sample location | |
| --- | --- | --- | --- | --- |
|  |  | Type of samples (faeces/water/  chicken surface swabs) |  |  |
|  |  |  | Kenyan City | Farm (PF) / Slaughterhouse (SH) |
|  |  |  |  |  |
|  |  |  |  |  |
| Enteritidis | Sal 16 | Droppings | Kiambu | PF2 |
|  | Sal 177 | Droppings | Kiambu | PF17 |
|  | Sal 568 | Droppings | Kiambu | PF54 |
|  | Sal 569 | Droppings | Kiambu | PF54 |
|  | Sal 572 | Droppings | Kiambu | PF54 |
|  | Sal 73 | Droppings | Kiambu | PF4 |
|  | Sal 312 | Droppings | Nairobi | PF53 |
| Heidelberg | Sal 157 | Water | Kiambu | PF13 |
|  | Sal 192 | Water | Kiambu | SH1 |
|  | Sal 194 | Water | Kiambu | SH1 |
|  | Sal 187 | Swab | Kiambu | PF17 |
|  | Sal 188 | Swab | Kiambu | PF17 |
| Kentucky | Sal 172 | Droppings | Kiambu | PF 17 |
|  | Sal 181 | Droppings | Kiambu | PF 17 |
|  | Sal 182 | Droppings | Kiambu | PF17 |
|  | Sal 571 | Droppings | Kiambu | PF54 |

Supplement Table 3: SNP variations and small in-del (insertion-deletion) found in the phage K2 reference genome in comparison to other phage genomes included in MT1-ST1.

| **Location of the variant** | **Locus**  **_tag** | **Function of the phage protein** | **Change**  **(K2 genome --> variant)** | **Polymorphism Type** | **Variant Frequency (%)** | **Number of genomes with the variant ^*^** |
| --- | --- | --- | --- | --- | --- | --- |
| 3048 | K2_003 | Structure | T -> C | SNP (transition) | 2.0% | 0.5 |
| 3057 | K2_003 | Structure | A -> T | SNP (transversion) | 2.0% | 0.5 |
| 3072 | K2_003 | Structure | T -> C | SNP (transition) | 2.0% | 0.5 |
| 3099 | K2_003 | Structure | C -> T | SNP (transition) | 2.0% | 0.5 |
| 3102 | K2_003 | Structure | A -> G | SNP (transition) | 2.0% | 0.5 |
| 3108 | K2_003 | Structure | C -> A | SNP (transversion) | 2.0% | 0.5 |
| 3109 to 3111 | K2_003 | Structure | AGG->CGT | Substitution | 2.0% | 0.5 |
| 3117 | K2_003 | Structure | C -> A | SNP (transversion) | 2.0% | 0.5 |
| 3144 | K2_003 | Structure | T -> G | SNP (transversion) | 2.0% | 0.5 |
| 3147 | K2_003 | Structure | G -> A | SNP (transition) | 2.0% | 0.5 |
| 3150 | K2_003 | Structure | C -> T | SNP (transition) | 2.0% | 0.5 |
| 3152 | K2_003 | Structure | T -> C | SNP (transition) | 2.0% | 0.5 |
| 3156 | K2_003 | Structure | G -> A | SNP (transition) | 2.0% | 0.5 |
| 3179 | K2_003 | Structure | A -> G | SNP (transition) | 2.0% | 0.5 |
| 3201 | K2_003 | Structure | A -> C | SNP (transversion) | 2.0% | 0.5 |
| 3206 to 3207 | K2_003 | Structure | CT -> AG | Substitution | 2.0% | 0.5 |
| 3214 to 3215 | K2_003 | Structure | AA -> CT | Substitution | 2.0% | 0.5 |
| 3217 | K2_003 | Structure | G -> A | SNP (transition) | 2.0% | 0.5 |
| 3223 to 3225 | K2_003 | Structure | GTG->ATT | Substitution | 2.0% | 0.5 |
| 3227 to 3228 | K2_003 | Structure | CT -> AA | Substitution | 2.0% | 0.5 |
| 5455 | K2_006 | Fibritin (wac) | C -> T | SNP (transition) | 36.0% | 9 |
| 5684 |  |  | AGTTATTCC | Insertion | 4.0% | 1 |
| 7436 | K2_010 | MCP | T -> C | SNP (transition) | 16.0% | 4 |
| 9580 | K2_014 |  | A -> G | SNP (transition) | 8.0% | 2 |
| 9785 | K2_015 |  | G -> A | SNP (transition) | 8.0% | 2 |
| 9966 | K2_015 |  | T -> A | SNP (transversion) | 4.0% | 1 |
| 10005 | K2_015 |  | T -> C | SNP (transition) | 4.0% | 1 |
| 10008 | K2_015 |  | T -> C | SNP (transition) | 4.0% | 1 |
| 10017 | K2_015 |  | T -> C | SNP (transition) | 4.0% | 1 |
| 10023 | K2_015 |  | T -> C | SNP (transition) | 4.0% | 1 |
| 10026 | K2_015 |  | T -> C | SNP (transition) | 4.0% | 1 |
| 10044 | K2_015 |  | G -> A | SNP (transition) | 4.0% | 1 |
| 10050 | K2_015 |  | G -> A | SNP (transition) | 4.0% | 1 |
| 10053 | K2_015 |  | G -> T | SNP (transversion) | 4.0% | 1 |
| 10068 | K2_015 |  | G -> A | SNP (transition) | 4.0% | 1 |
| 10071 | K2_015 |  | C -> G | SNP (transversion) | 4.0% | 1 |
| 10093 | K2_015 |  | C -> T | SNP (transition) | 4.0% | 1 |
| 10101 | K2_015 |  | C -> T | SNP (transition) | 4.0% | 1 |
| 10116 | K2_015 |  | T -> A | SNP (transversion) | 4.0% | 1 |
| 10122 | K2_015 |  | C -> A | SNP (transversion) | 4.0% | 1 |
| 10127 to 10128 | K2_015 |  | TC -> CG | Substitution | 4.0% | 1 |
| 10134 | K2_015 |  | G -> A | SNP (transition) | 4.0% | 1 |
| 10140 | K2_015 |  | T -> G | SNP (transversion) | 4.0% | 1 |
| 10147 | K2_015 |  | G -> A | SNP (transition) | 4.0% | 1 |
| 10455 | K2_016 |  | T -> C | SNP (transition) | 4.0% | 1 |
| 10469 | K2_016 |  | T -> C | SNP (transition) | 4.0% | 1 |
| 10783 | K2_017 |  | T -> C | SNP (transition) | 4.0% | 1 |
| 10811 | K2_017 |  | A -> C | SNP (transversion) | 12.0% | 3 |
| 10985 | K2_017 |  | T -> C | SNP (transition) | 4.0% | 1 |
| 11125 | K2_018 |  | T -> A | SNP (transversion) | 4.0% | 1 |
| 11187 | K2_018 |  | G -> A | SNP (transition) | 4.0% | 1 |
| 11205 | K2_018 |  | G -> A | SNP (transition) | 4.0% | 1 |
| 11432 | K2_018 |  | T -> C | SNP (transition) | 16.0% | 4 |
| 11478 | K2_018 |  | C -> T | SNP (transition) | 16.0% | 4 |
| 11484 | K2_018 |  | A -> C | SNP (transversion) | 16.0% | 4 |
| 11525 | K2_019 |  | A -> G | SNP (transition) | 16.0% | 4 |
| 11554 | K2_019 |  | G -> T | SNP (transversion) | 16.0% | 4 |
| 11559 to 11156 | K2_019 |  | GAA->ACG | Substitution | 16.0% | 4 |
| 11564 | K2_019 |  | C -> G | SNP (transversion) | 16.0% | 4 |
| 11570 | K2_019 |  | G -> T | SNP (transversion) | 16.0% | 4 |
| 11579 | K2_019 |  | A -> C | SNP (transversion) | 16.0% | 4 |
| 11581 | K2_019 |  | C -> A | SNP (transversion) | 16.0% | 4 |
| 11585 | K2_019 |  | C -> T | SNP (transition) | 16.0% | 4 |
| 11591 | K2_019 |  | A -> G | SNP (transition) | 16.0% | 4 |
| 11600 | K2_019 |  | A -> G | SNP (transition) | 16.0% | 4 |
| 11612 | K2_019 |  | A -> T | SNP (transversion) | 16.0% | 4 |
| 11621 | K2_019 |  | C -> T | SNP (transition) | 16.0% | 4 |
| 11624 | K2_019 |  | C -> G | SNP (transversion) | 16.0% | 4 |
| 11627 | K2_019 |  | G -> C | SNP (transversion) | 16.0% | 4 |
| 11633 | K2_019 |  | C -> G | SNP (transversion) | 16.0% | 4 |
| 11636 | K2_019 |  | A -> T | SNP (transversion) | 16.0% | 4 |
| 11654 | K2_019 |  | T -> C | SNP (transition) | 16.0% | 4 |
| 11671 | K2_019 |  | A -> G | SNP (transition) | 16.0% | 4 |
| 11682 | K2_019 |  | G -> T | SNP (transversion) | 16.0% | 4 |
| 11693 | K2_019 |  | T -> A | SNP (transversion) | 16.0% | 4 |
| 11696 | K2_019 |  | C -> T | SNP (transition) | 16.0% | 4 |
| 11702 | K2_019 |  | T -> A | SNP (transversion) | 16.0% | 4 |
| 11711 | K2_019 |  | A -> T | SNP (transversion) | 16.0% | 4 |
| 11717 | K2_019 |  | G -> A | SNP (transition) | 16.0% | 4 |
| 11724 to 11726 | K2_019 |  | ATT->GTC | Substitution | 16.0% | 4 |
| 11729 | K2_019 |  | G -> A | SNP (transition) | 16.0% | 4 |
| 11732 | K2_019 |  | A -> G | SNP (transition) | 16.0% | 4 |
| 11735 | K2_019 |  | T -> C | SNP (transition) | 16.0% | 4 |
| 11739 to 11740 | K2_019 |  | GA -> AC | Substitution | 16.0% | 4 |
| 11747 | K2_019 |  | A -> G | SNP (transition) | 16.0% | 4 |
| 11759 | K2_019 |  | A -> G | SNP (transition) | 16.0% | 4 |
| 11762 | K2_019 |  | A -> C | SNP (transversion) | 16.0% | 4 |
| 11765 | K2_019 |  | C -> T | SNP (transition) | 16.0% | 4 |
| 11774 | K2_019 |  | T -> C | SNP (transition) | 16.0% | 4 |
| 11789 | K2_019 |  | G -> C | SNP (transversion) | 16.0% | 4 |
| 11807 | K2_019 |  | A -> G | SNP (transition) | 16.0% | 4 |
| 11814 to 11816 | K2_019 |  | GTC->ATT | Substitution | 16.0% | 4 |
| 11819 | K2_019 |  | A -> G | SNP (transition) | 16.0% | 4 |
| 11822 | K2_019 |  | A -> T | SNP (transversion) | 16.0% | 4 |
| 11828 | K2_019 |  | C -> G | SNP (transversion) | 16.0% | 4 |
| 11853 | K2_019 |  | G -> T | SNP (transversion) | 16.0% | 4 |
| 11867 | K2_019 |  | G -> A | SNP (transition) | 16.0% | 4 |
| 11870 | K2_019 |  | A -> C | SNP (transversion) | 16.0% | 4 |
| 11873 | K2_019 |  | C -> T | SNP (transition) | 16.0% | 4 |
| 11879 | K2_019 |  | C -> T | SNP (transition) | 16.0% | 4 |
| 11888 | K2_019 |  | T -> C | SNP (transition) | 16.0% | 4 |
| 11903 | K2_019 |  | T -> C | SNP (transition) | 16.0% | 4 |
| 11919 | K2_019 |  | C -> A | SNP (transversion) | 16.0% | 4 |
| 11954 | K2_019 |  | C -> G | SNP (transversion) | 16.0% | 4 |
| 11960 | K2_019 |  | A -> G | SNP (transition) | 16.0% | 4 |
| 11963 | K2_019 |  | C -> A | SNP (transversion) | 16.0% | 4 |
| 11978 | K2_019 |  | T -> C | SNP (transition) | 16.0% | 4 |
| 12005 | K2_019 |  | C -> A | SNP (transversion) | 16.0% | 4 |
| 12010 | K2_019 |  | T -> C | SNP (transition) | 16.0% | 4 |
| 12025 | K2_019 |  | G -> A | SNP (transition) | 16.0% | 4 |
| 12065 | K2_019 |  | A -> C | SNP (transversion) | 16.0% | 4 |
| 12071 | K2_019 |  | G -> A | SNP (transition) | 16.0% | 4 |
| 12074 | K2_019 |  | T -> C | SNP (transition) | 16.0% | 4 |
| 12077 | K2_019 |  | T -> C | SNP (transition) | 16.0% | 4 |
| 12080 | K2_019 |  | G -> A | SNP (transition) | 16.0% | 4 |
| 12095 | K2_019 |  | G -> A | SNP (transition) | 16.0% | 4 |
| 12116 | K2_019 |  | T -> C | SNP (transition) | 4.0% | 1 |
| 12128 | K2_019 |  | C -> T | SNP (transition) | 4.0% | 1 |
| 12131 | K2_019 |  | C -> T | SNP (transition) | 4.0% | 1 |
| 12242 | K2_019 |  | A -> C | SNP (transversion) | 4.0% | 1 |
| 12254 | K2_019 |  | C -> T | SNP (transition) | 4.0% | 1 |
| 12263 | K2_019 |  | C -> G | SNP (transversion) | 4.0% | 1 |
| 12269 | K2_019 |  | T -> C | SNP (transition) | 4.0% | 1 |
| 12281 | K2_019 |  | A -> G | SNP (transition) | 4.0% | 1 |
| 12294 | K2_019 |  | G -> A | SNP (transition) | 4.0% | 1 |
| 12308 | K2_019 |  | T -> C | SNP (transition) | 4.0% | 1 |
| 12314 | K2_019 |  | G -> A | SNP (transition) | 4.0% | 1 |
| 12326 | K2_019 |  | T -> C | SNP (transition) | 4.0% | 1 |
| 12329 | K2_019 |  | T -> C | SNP (transition) | 4.0% | 1 |
| 12377 | K2_019 |  | G -> A | SNP (transition) | 4.0% | 1 |
| 12380 | K2_019 |  | A -> G | SNP (transition) | 4.0% | 1 |
| 12386 | K2_019 |  | C -> T | SNP (transition) | 4.0% | 1 |
| 12403 | K2_019 |  | A -> G | SNP (transition) | 4.0% | 1 |
| 12408 | K2_019 |  | A -> G | SNP (transition) | 4.0% | 1 |
| 12413 | K2_019 |  | C -> T | SNP (transition) | 4.0% | 1 |
| 12416 | K2_019 |  | G -> T | SNP (transversion) | 4.0% | 1 |
| 12425 | K2_019 |  | C -> T | SNP (transition) | 4.0% | 1 |
| 12434 | K2_019 |  | G -> T | SNP (transversion) | 4.0% | 1 |
| 12437 | K2_019 |  | A -> T | SNP (transversion) | 4.0% | 1 |
| 12515 | K2_019 |  | T -> C | SNP (transition) | 4.0% | 1 |
| 12518 | K2_019 |  | G -> A | SNP (transition) | 4.0% | 1 |
| 12524 | K2_019 |  | C -> T | SNP (transition) | 4.0% | 1 |
| 12539 | K2_019 |  | A -> G | SNP (transition) | 4.0% | 1 |
| 13466 |  |  | (A)9 -> (A)8 | Deletion (TR) | 24.0% | 6 |
| 13720 |  |  | C -> A | SNP (transversion) | 8.0% | 2 |
| 13739 | K2_022 | HNH homing endonuclease | T -> C | SNP (transition) | 4.0% | 1 |
| 13859 | K2_022 | HNH homing endonuclease | A -> T | SNP (transversion) | 16.0% | 4 |
| 13898 | K2_022 | HNH homing endonuclease | G -> A | SNP (transition) | 8.0% | 2 |
| 14194 | K2_022 | HNH homing endonuclease | A -> G | SNP (transition) | 8.0% | 2 |
| 14350 | K2_023 |  | A -> C | SNP (transversion) | 4.0% | 1 |
| 16153 | K2_025 |  | C -> T | SNP (transition) | 20.0% | 5 |
| 16867 | K2_027 | TMP | A -> C | SNP (transversion) | 8.0% | 2 |
| 16895 | K2_027 | TMP | T -> G | SNP (transversion) | 4.0% | 1 |
| 17132 | K2_027 | TMP | G -> A | SNP (transition) | 8.0% | 2 |
| 17252 | K2_027 | TMP | A -> G | SNP (transition) | 8.0% | 2 |
| 18614 | K2_027 | TMP | G -> A | SNP (transition) | 4.0% | 1 |
| 19891 | K2_029 |  | A -> G | SNP (transition) | 8.0% | 2 |
| 19906 | K2_029 |  | T -> C | SNP (transition) | 12.0% | 3 |
| 19930 | K2_029 |  | A -> G | SNP (transition) | 12.0% | 3 |
| 19930 | K2_030 |  | A -> G | SNP (transition) | 12.0% | 3 |
| 19959 | K2_030 |  | G -> A | SNP (transition) | 8.0% | 2 |
| 19968 | K2_030 |  | T -> C | SNP (transition) | 8.0% | 2 |
| 19971 | K2_030 |  | C -> T | SNP (transition) | 8.0% | 2 |
| 19974 | K2_030 |  | T -> C | SNP (transition) | 4.0% | 1 |
| 20007 | K2_030 |  | T -> C | SNP (transition) | 12.0% | 3 |
| 20016 | K2_030 |  | A -> G | SNP (transition) | 4.0% | 1 |
| 20016 | K2_030 |  | A -> T | SNP (transversion) | 8.0% | 2 |
| 20019 to 20023 | K2_030 |  | ACTGC -> GTTAT | Substitution | 4.0% | 1 |
| 20019 to 20025 | K2_030 |  | ACTGCCG->GTTATCA | Substitution | 8.0% | 2 |
| 20028 | K2_030 |  | C -> T | SNP (transition) | 8.0% | 2 |
| 20031 | K2_030 |  | A -> G | SNP (transition) | 4.0% | 1 |
| 20037 | K2_030 |  | T -> C | SNP (transition) | 4.0% | 1 |
| 20040 | K2_030 |  | T -> C | SNP (transition) | 8.0% | 2 |
| 20043 | K2_030 |  | A -> C | SNP (transversion) | 8.0% | 2 |
| 20046 | K2_030 |  | C -> A | SNP (transversion) | 4.0% | 1 |
| 20063 | K2_030 |  | A -> G | SNP (transition) | 8.0% | 2 |
| 20064 | K2_030 |  | T -> C | SNP (transition) | 4.0% | 1 |
| 20065 to 20067 | K2_030 |  | TCT ->GCC | Substitution | 4.0% | 1 |
| 20065 to 20067 | K2_030 |  | TCT ->GCG | Substitution | 8.0% | 2 |
| 20070 | K2_030 |  | C -> T | SNP (transition) | 8.0% | 2 |
| 20079 | K2_030 |  | C -> T | SNP (transition) | 4.0% | 1 |
| 20086 | K2_030 |  | G -> T | SNP (transversion) | 4.0% | 1 |
| 20100 | K2_030 |  | C -> A | SNP (transversion) | 12.0% | 3 |
| 20101 | K2_030 |  | C -> T | SNP (transition) | 4.0% | 1 |
| 20109 | K2_030 |  | T -> C | SNP (transition) | 4.0% | 1 |
| 20116 to 20117 | K2_030 |  | CA -> AT | Substitution | 4.0% | 1 |
| 20121 | K2_030 |  | C -> T | SNP (transition) | 4.0% | 1 |
| 20130 | K2_030 |  | C -> T | SNP (transition) | 12.0% | 3 |
| 20178 | K2_030 |  | T -> C | SNP (transition) | 12.0% | 3 |
| 20205 | K2_030 |  | A -> T | SNP (transversion) | 12.0% | 3 |
| 20357 | K2_031 |  | A -> G | SNP (transition) | 12.0% | 3 |
| 20378 | K2_031 |  | T -> C | SNP (transition) | 12.0% | 3 |
| 20384 | K2_031 |  | G -> C | SNP (transversion) | 12.0% | 3 |
| 20387 | K2_031 |  | C -> T | SNP (transition) | 12.0% | 3 |
| 20393 | K2_031 |  | T -> G | SNP (transversion) | 12.0% | 3 |
| 20399 | K2_031 |  | A -> T | SNP (transversion) | 12.0% | 3 |
| 20402 | K2_031 |  | T -> C | SNP (transition) | 12.0% | 3 |
| 20411 | K2_031 |  | T -> C | SNP (transition) | 12.0% | 3 |
| 20435 | K2_031 |  | C -> T | SNP (transition) | 12.0% | 3 |
| 20492 | K2_031 |  | T -> C | SNP (transition) | 12.0% | 3 |
| 20495 | K2_031 |  | C -> G | SNP (transversion) | 12.0% | 3 |
| 20501 | K2_031 |  | C -> T | SNP (transition) | 12.0% | 3 |
| 20510 | K2_031 |  | T -> C | SNP (transition) | 12.0% | 3 |
| 20538 | K2_031 |  | G -> A | SNP (transition) | 12.0% | 3 |
| 20552 | K2_031 |  | A -> G | SNP (transition) | 12.0% | 3 |
| 20562 | K2_031 |  | T -> C | SNP (transition) | 12.0% | 3 |
| 20567 | K2_031 |  | A -> G | SNP (transition) | 12.0% | 3 |
| 20582 | K2_031 |  | G -> T | SNP (transversion) | 12.0% | 3 |
| 20585 | K2_031 |  | G -> A | SNP (transition) | 12.0% | 3 |
| 20586 | K2_031 |  | T -> C | SNP (transition) | 4.0% | 1 |
| 20588 | K2_031 |  | A -> C | SNP (transversion) | 12.0% | 3 |
| 20597 | K2_031 |  | T -> G | SNP (transversion) | 12.0% | 3 |
| 20621 | K2_031 |  | G -> A | SNP (transition) | 12.0% | 3 |
| 20636 | K2_031 |  | C -> T | SNP (transition) | 12.0% | 3 |
| 20654 | K2_031 |  | C -> G | SNP (transversion) | 12.0% | 3 |
| 20657 | K2_031 |  | T -> C | SNP (transition) | 12.0% | 3 |
| 20660 | K2_031 |  | A -> G | SNP (transition) | 12.0% | 3 |
| 20666 | K2_031 |  | G -> A | SNP (transition) | 12.0% | 3 |
| 20669 | K2_031 |  | T -> C | SNP (transition) | 12.0% | 3 |
| 20672 | K2_031 |  | C -> T | SNP (transition) | 12.0% | 3 |
| 20699 | K2_031 |  | T -> C | SNP (transition) | 12.0% | 3 |
| 20705 | K2_031 |  | A -> C | SNP (transversion) | 12.0% | 3 |
| 20718 | K2_031 |  | T -> A | SNP (transversion) | 12.0% | 3 |
| 20744 | K2_031 |  | T -> C | SNP (transition) | 12.0% | 3 |
| 20765 | K2_031 |  | C -> G | SNP (transversion) | 4.0% | 2 |
| 20765 | K2_031 |  | C -> T | SNP (transition) | 8.0% | 1 |
| 20786 | K2_031 |  | T -> A | SNP (transversion) | 12.0% | 3 |
| 20804 | K2_031 |  | G -> T | SNP (transversion) | 12.0% | 3 |
| 20807 | K2_031 |  | T -> A | SNP (transversion) | 12.0% | 3 |
| 20810 | K2_031 |  | C -> T | SNP (transition) | 12.0% | 3 |
| 20816 | K2_031 |  | G -> T | SNP (transversion) | 12.0% | 3 |
| 20825 | K2_031 |  | T -> C | SNP (transition) | 12.0% | 3 |
| 20831 | K2_031 |  | T -> C | SNP (transition) | 12.0% | 3 |
| 20840 | K2_031 |  | T -> C | SNP (transition) | 12.0% | 3 |
| 20861 | K2_031 |  | G -> C | SNP (transversion) | 12.0% | 3 |
| 20867 | K2_031 |  | T -> C | SNP (transition) | 12.0% | 3 |
| 20870 | K2_031 |  | C -> A | SNP (transversion) | 12.0% | 3 |
| 20873 | K2_031 |  | G -> A | SNP (transition) | 12.0% | 3 |
| 20876 | K2_031 |  | C -> T | SNP (transition) | 12.0% | 3 |
| 20885 | K2_031 |  | C -> G | SNP (transversion) | 12.0% | 3 |
| 20915 | K2_031 |  | A -> C | SNP (transversion) | 12.0% | 3 |
| 20933 | K2_031 |  | G -> A | SNP (transition) | 12.0% | 3 |
| 20939 | K2_031 |  | G -> A | SNP (transition) | 12.0% | 3 |
| 20966 | K2_031 |  | C -> T | SNP (transition) | 12.0% | 3 |
| 20987 | K2_031 |  | G -> A | SNP (transition) | 12.0% | 3 |
| 20990 | K2_031 |  | C -> G | SNP (transversion) | 12.0% | 3 |
| 20996 | K2_031 |  | C -> T | SNP (transition) | 12.0% | 3 |
| 21002 | K2_031 |  | T -> C | SNP (transition) | 12.0% | 3 |
| 21048 | K2_031 |  | G -> A | SNP (transition) | 12.0% | 3 |
| 21095 to 21096 | K2_031 |  | GT -> AC | Substitution | 8.0% | 2 |
| 21101 | K2_031 |  | T -> G | SNP (transversion) | 8.0% | 2 |
| 21113 | K2_031 |  | G -> A | SNP (transition) | 8.0% | 2 |
| 21120 | K2_031 |  | T -> C | SNP (transition) | 8.0% | 2 |
| 21143 | K2_031 |  | G -> A | SNP (transition) | 8.0% | 2 |
| 21149 | K2_031 |  | C -> T | SNP (transition) | 12.0% | 3 |
| 21153 to 21155 | K2_031 |  | TTG -> CTC | Substitution | 8.0% | 2 |
| 21167 | K2_031 |  | G -> A | SNP (transition) | 8.0% | 2 |
| 21170 | K2_031 |  | C -> T | SNP (transition) | 12.0% | 3 |
| 21176 to 21179 | K2_031 |  | GTTA-AGTC | Substitution | 8.0% | 2 |
| 21185 | K2_031 |  | A -> G | SNP (transition) | 8.0% | 2 |
| 21188 | K2_031 |  | T -> C | SNP (transition) | 12.0% | 3 |
| 21198 | K2_031 |  | A -> G | SNP (transition) | 8.0% | 2 |
| 21215 | K2_031 |  | C -> T | SNP (transition) | 12.0% | 3 |
| 21224 | K2_031 |  | T -> C | SNP (transition) | 4.0% | 1 |
| 21227 | K2_031 |  | T -> C | SNP (transition) | 12.0% | 3 |
| 21251 | K2_031 |  | A -> C | SNP (transversion) | 36.0% | 9 |
| 21272 | K2_031 |  | C -> T | SNP (transition) | 8.0% | 2 |
| 21275 | K2_031 |  | A -> G | SNP (transition) | 28.0% | 7 |
| 21278 | K2_031 |  | G -> A | SNP (transition) | 28.0% | 7 |
| 21281 | K2_031 |  | G -> C | SNP (transversion) | 8.0% | 2 |
| 21293 | K2_031 |  | G -> A | SNP (transition) | 8.0% | 2 |
| 21296 | K2_031 |  | T -> C | SNP (transition) | 8.0% | 2 |
| 21299 | K2_031 |  | T -> C | SNP (transition) | 36.0% | 9 |
| 21302 | K2_031 |  | A -> G | SNP (transition) | 36.0% | 9 |
| 21305 | K2_031 |  | C -> T | SNP (transition) | 8.0% | 2 |
| 21329 | K2_031 |  | G -> T | SNP (transversion) | 8.0% | 2 |
| 21332 | K2_031 |  | T -> A | SNP (transversion) | 8.0% | 2 |
| 21341 | K2_031 |  | C -> T | SNP (transition) | 8.0% | 2 |
| 21356 | K2_031 |  | C -> T | SNP (transition) | 8.0% | 2 |
| 21371 | K2_031 |  | A -> C | SNP (transversion) | 4.0% | 2 |
| 21371 | K2_031 |  | A -> T | SNP (transversion) | 8.0% | 1 |
| 21374 | K2_031 |  | C -> G | SNP (transversion) | 4.0% | 1 |
| 21377 | K2_031 |  | G -> C | SNP (transversion) | 12.0% | 3 |
| 21380 | K2_031 |  | C -> A | SNP (transversion) | 4.0% | 1 |
| 21380 | K2_031 |  | C -> G | SNP (transversion) | 8.0% | 2 |
| 21383 | K2_031 |  | T -> C | SNP (transition) | 8.0% | 2 |
| 21386 | K2_031 |  | G -> C | SNP (transversion) | 12.0% | 3 |
| 21392 | K2_031 |  | C -> T | SNP (transition) | 12.0% | 3 |
| 21407 | K2_031 |  | G -> A | SNP (transition) | 8.0% | 2 |
| 21410 | K2_031 |  | A -> G | SNP (transition) | 8.0% | 2 |
| 21417 | K2_031 |  | C -> T | SNP (transition) | 8.0% | 2 |
| 21425 | K2_031 |  | T -> G | SNP (transversion) | 4.0% | 1 |
| 21428 | K2_031 |  | T -> A | SNP (transversion) | 8.0% | 2 |
| 21434 | K2_031 |  | C -> T | SNP (transition) | 4.0% | 1 |
| 21437 | K2_031 |  | G -> A | SNP (transition) | 8.0% | 2 |
| 21440 | K2_031 |  | A -> T | SNP (transversion) | 4.0% | 1 |
| 21446 | K2_031 |  | C -> T | SNP (transition) | 8.0% | 2 |
| 21449 to 21452 | K2_031 |  | GCTA-ATTG | Substitution | 8.0% | 2 |
| 21455 | K2_031 |  | T -> C | SNP (transition) | 8.0% | 2 |
| 21464 | K2_031 |  | T -> C | SNP (transition) | 8.0% | 2 |
| 21468 to 21469 | K2_031 |  | GT -> AC | Substitution | 8.0% | 2 |
| 21476 | K2_031 |  | C -> T | SNP (transition) | 8.0% | 2 |
| 21479 | K2_031 |  | G -> A | SNP (transition) | 8.0% | 2 |
| 21482 | K2_031 |  | A -> G | SNP (transition) | 8.0% | 2 |
| 21488 | K2_031 |  | C -> T | SNP (transition) | 8.0% | 2 |
| 21491 | K2_031 |  | G -> C | SNP (transversion) | 8.0% | 2 |
| 21491 | K2_031 |  | G -> T | SNP (transversion) | 8.0% | 2 |
| 21494 | K2_031 |  | G -> A | SNP (transition) | 8.0% | 2 |
| 21503 | K2_031 |  | C -> T | SNP (transition) | 8.0% | 2 |
| 21515 | K2_031 |  | T -> A | SNP (transversion) | 8.0% | 2 |
| 21518 | K2_031 |  | T -> A | SNP (transversion) | 8.0% | 2 |
| 21518 | K2_031 |  | T -> C | SNP (transition) | 8.0% | 2 |
| 21521 | K2_031 |  | A -> G | SNP (transition) | 8.0% | 2 |
| 21533 | K2_031 |  | T -> C | SNP (transition) | 8.0% | 2 |
| 21542 | K2_031 |  | T -> C | SNP (transition) | 8.0% | 2 |
| 21554 | K2_031 |  | A -> G | SNP (transition) | 16.0% | 4 |
| 21560 | K2_031 |  | C -> T | SNP (transition) | 16.0% | 4 |
| 21566 | K2_031 |  | G -> A | SNP (transition) | 8.0% | 2 |
| 21567 | K2_031 |  | A -> C | SNP (transversion) | 16.0% | 4 |
| 21575 | K2_031 |  | T -> G | SNP (transversion) | 16.0% | 4 |
| 21581 | K2_031 |  | G -> C | SNP (transversion) | 16.0% | 4 |
| 21588 | K2_031 |  | C -> A | SNP (transversion) | 16.0% | 4 |
| 21593 | K2_031 |  | G -> A | SNP (transition) | 16.0% | 4 |
| 21596 | K2_031 |  | A -> G | SNP (transition) | 8.0% | 2 |
| 21602 | K2_031 |  | T -> C | SNP (transition) | 8.0% | 2 |
| 21602 | K2_031 |  | T -> G | SNP (transversion) | 8.0% | 2 |
| 21623 | K2_031 |  | T -> C | SNP (transition) | 8.0% | 2 |
| 21629 | K2_031 |  | T -> C | SNP (transition) | 16.0% | 4 |
| 21636 | K2_031 |  | A -> G | SNP (transition) | 8.0% | 2 |
| 21641 | K2_031 |  | A -> G | SNP (transition) | 16.0% | 4 |
| 21644 | K2_031 |  | T -> C | SNP (transition) | 8.0% | 2 |
| 21653 | K2_031 |  | G -> A | SNP (transition) | 8.0% | 2 |
| 21668 | K2_031 |  | C -> T | SNP (transition) | 8.0% | 2 |
| 21671 | K2_031 |  | T -> C | SNP (transition) | 8.0% | 2 |
| 21677 | K2_031 |  | T -> C | SNP (transition) | 16.0% | 4 |
| 21680 | K2_031 |  | G -> A | SNP (transition) | 8.0% | 2 |
| 21695 | K2_031 |  | G -> A | SNP (transition) | 8.0% | 2 |
| 21698 | K2_031 |  | T -> A | SNP (transversion) | 8.0% | 2 |
| 21698 | K2_031 |  | T -> C | SNP (transition) | 8.0% | 2 |
| 21701 | K2_031 |  | C -> A | SNP (transversion) | 8.0% | 2 |
| 21710 | K2_031 |  | A -> G | SNP (transition) | 8.0% | 2 |
| 21725 | K2_031 |  | A -> C | SNP (transversion) | 10.0% | 2.5 |
| 21728 | K2_031 |  | A -> C | SNP (transversion) | 10.0% | 2.5 |
| 21731 | K2_031 |  | C -> T | SNP (transition) | 10.0% | 2.5 |
| 21734 | K2_031 |  | C -> T | SNP (transition) | 10.0% | 2.5 |
| 21743 | K2_031 |  | C -> A | SNP (transversion) | 10.0% | 2.5 |
| 21746 | K2_031 |  | A -> G | SNP (transition) | 10.0% | 2.5 |
| 21749 | K2_031 |  | G -> A | SNP (transition) | 10.0% | 2.5 |
| 21752 | K2_031 |  | A -> G | SNP (transition) | 10.0% | 2.5 |
| 21758 | K2_031 |  | A -> G | SNP (transition) | 10.0% | 2.5 |
| 21761 | K2_031 |  | C -> T | SNP (transition) | 18.0% | 4.5 |
| 21770 | K2_031 |  | C -> T | SNP (transition) | 10.0% | 2.5 |
| 21773 | K2_031 |  | C -> T | SNP (transition) | 10.0% | 2.5 |
| 21782 | K2_031 |  | A -> G | SNP (transition) | 10.0% | 2.5 |
| 21783 | K2_031 |  | C -> T | SNP (transition) | 10.0% | 2.5 |
| 21794 | K2_031 |  | T -> C | SNP (transition) | 10.0% | 2.5 |
| 21800 | K2_031 |  | T -> C | SNP (transition) | 18.0% | 4.5 |
| 21824 | K2_031 |  | C -> A | SNP (transversion) | 10.0% | 2.5 |
| 21827 | K2_031 |  | C -> T | SNP (transition) | 10.0% | 2.5 |
| 21833 | K2_031 |  | C -> A | SNP (transversion) | 18.0% | 4.5 |
| 21836 | K2_031 |  | A -> G | SNP (transition) | 18.0% | 4.5 |
| 21839 | K2_031 |  | C -> T | SNP (transition) | 18.0% | 4.5 |
| 21842 | K2_031 |  | G -> A | SNP (transition) | 18.0% | 4.5 |
| 21848 | K2_031 |  | T -> C | SNP (transition) | 16.0% | 4 |
| 21851 | K2_031 |  | T -> C | SNP (transition) | 16.0% | 4 |
| 21863 | K2_031 |  | C -> A | SNP (transversion) | 8.0% | 2 |
| 21863 | K2_031 |  | C -> G | SNP (transversion) | 8.0% | 2 |
| 21872 | K2_031 |  | G -> A | SNP (transition) | 8.0% | 2 |
| 21875 | K2_031 |  | T -> C | SNP (transition) | 8.0% | 2 |
| 21881 | K2_031 |  | A -> C | SNP (transversion) | 8.0% | 2 |
| 21881 | K2_031 |  | A -> G | SNP (transition) | 8.0% | 2 |
| 21926 | K2_031 |  | G -> T | SNP (transversion) | 8.0% | 2 |
| 21950 | K2_031 |  | C -> T | SNP (transition) | 16.0% | 4 |
| 21953 | K2_031 |  | T -> C | SNP (transition) | 8.0% | 2 |
| 21965 | K2_031 |  | G -> C | SNP (transversion) | 16.0% | 4 |
| 21968 | K2_031 |  | G -> A | SNP (transition) | 8.0% | 2 |
| 21977 | K2_031 |  | T -> C | SNP (transition) | 8.0% | 2 |
| 22031 | K2_031 |  | C -> T | SNP (transition) | 8.0% | 2 |
| 22049 | K2_031 |  | T -> C | SNP (transition) | 8.0% | 2 |
| 22056 | K2_031 |  | A -> C | SNP (transversion) | 8.0% | 2 |
| 22061 | K2_031 |  | T -> C | SNP (transition) | 8.0% | 2 |
| 22088 | K2_031 |  | A -> G | SNP (transition) | 8.0% | 2 |
| 22103 | K2_031 |  | T -> C | SNP (transition) | 16.0% | 4 |
| 22115 | K2_031 |  | G -> A | SNP (transition) | 8.0% | 2 |
| 22130 | K2_031 |  | C -> T | SNP (transition) | 8.0% | 2 |
| 22136 | K2_031 |  | C -> T | SNP (transition) | 4.0% | 1 |
| 22145 | K2_031 |  | T -> C | SNP (transition) | 12.0% | 3 |
| 22151 | K2_031 |  | G -> A | SNP (transition) | 12.0% | 3 |
| 22163 | K2_031 |  | A -> C | SNP (transversion) | 12.0% | 3 |
| 22173 | K2_031 |  | T -> G | SNP (transversion) | 12.0% | 3 |
| 22181 | K2_031 |  | T -> G | SNP (transversion) | 12.0% | 3 |
| 22184 | K2_031 |  | T -> C | SNP (transition) | 12.0% | 3 |
| 22187 | K2_031 |  | C -> T | SNP (transition) | 12.0% | 3 |
| 22190 | K2_031 |  | C -> T | SNP (transition) | 12.0% | 3 |
| 22195 | K2_031 |  | A -> G | SNP (transition) | 8.0% | 2 |
| 22214 | K2_031 |  | C -> T | SNP (transition) | 12.0% | 3 |
| 22241 | K2_031 |  | C -> A | SNP (transversion) | 12.0% | 3 |
| 22244 | K2_031 |  | T -> A | SNP (transversion) | 12.0% | 3 |
| 22247 | K2_031 |  | A -> T | SNP (transversion) | 12.0% | 3 |
| 22256 | K2_031 |  | A -> G | SNP (transition) | 12.0% | 3 |
| 22298 | K2_031 |  | T -> C | SNP (transition) | 12.0% | 3 |
| 22319 | K2_031 |  | C -> G | SNP (transversion) | 12.0% | 3 |
| 22331 | K2_031 |  | T -> C | SNP (transition) | 12.0% | 3 |
| 22334 | K2_031 |  | T -> C | SNP (transition) | 12.0% | 3 |
| 22346 | K2_031 |  | A -> T | SNP (transversion) | 12.0% | 3 |
| 22349 | K2_031 |  | G -> A | SNP (transition) | 12.0% | 3 |
| 22355 | K2_031 |  | G -> A | SNP (transition) | 12.0% | 3 |
| 22361 | K2_031 |  | A -> G | SNP (transition) | 12.0% | 3 |
| 22367 | K2_031 |  | T -> G | SNP (transversion) | 12.0% | 3 |
| 22372 | K2_031 |  | G -> C | SNP (transversion) | 12.0% | 3 |
| 22376 | K2_031 |  | T -> C | SNP (transition) | 12.0% | 3 |
| 22385 | K2_031 |  | A -> G | SNP (transition) | 12.0% | 3 |
| 22388 | K2_031 |  | A -> T | SNP (transversion) | 12.0% | 3 |
| 22391 | K2_031 |  | C -> T | SNP (transition) | 12.0% | 3 |
| 22394 | K2_031 |  | T -> C | SNP (transition) | 8.0% | 2 |
| 22400 | K2_031 |  | G -> T | SNP (transversion) | 12.0% | 3 |
| 22403 | K2_031 |  | A -> G | SNP (transition) | 12.0% | 3 |
| 22427 | K2_031 |  | A -> G | SNP (transition) | 12.0% | 3 |
| 22433 | K2_031 |  | C -> G | SNP (transversion) | 12.0% | 3 |
| 22440 to 22442 | K2_031 |  | GCC->TCT | Substitution | 8.0% | 2 |
| 22442 | K2_031 |  | C -> T | SNP (transition) | 4.0% | 1 |
| 22445 | K2_031 |  | C -> T | SNP (transition) | 12.0% | 3 |
| 22463 | K2_031 |  | T -> C | SNP (transition) | 12.0% | 3 |
| 22469 | K2_031 |  | G -> T | SNP (transversion) | 12.0% | 3 |
| 22475 | K2_031 |  | C -> T | SNP (transition) | 12.0% | 3 |
| 22478 | K2_031 |  | C -> T | SNP (transition) | 12.0% | 3 |
| 22490 | K2_031 |  | T -> A | SNP (transversion) | 12.0% | 3 |
| 22493 | K2_031 |  | A -> C | SNP (transversion) | 12.0% | 3 |
| 22499 | K2_031 |  | A -> G | SNP (transition) | 12.0% | 3 |
| 22511 | K2_031 |  | C -> A | SNP (transversion) | 12.0% | 3 |
| 22517 | K2_031 |  | G -> A | SNP (transition) | 12.0% | 3 |
| 22526 | K2_031 |  | T -> C | SNP (transition) | 8.0% | 2 |
| 22528 | K2_031 |  | C -> A | SNP (transversion) | 12.0% | 3 |
| 22538 | K2_031 |  | T -> G | SNP (transversion) | 12.0% | 3 |
| 22542 to 22547 | K2_031 |  | CTGAGA  -> TTACGT | Substitution | 12.0% | 3 |
| 22550 | K2_031 |  | C -> T | SNP (transition) | 4.0% | 1 |
| 22553 | K2_031 |  | T -> C | SNP (transition) | 4.0% | 1 |
| 22556 | K2_031 |  | C -> A | SNP (transversion) | 4.0% | 1 |
| 22571 | K2_031 |  | C -> T | SNP (transition) | 4.0% | 1 |
| 22577 | K2_031 |  | T -> C | SNP (transition) | 12.0% | 3 |
| 22586 | K2_031 |  | A -> G | SNP (transition) | 4.0% | 1 |
| 22589 | K2_031 |  | C -> T | SNP (transition) | 4.0% | 1 |
| 22613 | K2_031 |  | G -> T | SNP (transversion) | 24.0% | 6 |
| 22640 | K2_031 |  | T -> C | SNP (transition) | 4.0% | 1 |
| 22715 | K2_031 |  | A -> G | SNP (transition) | 20.0% | 5 |
| 22724 | K2_031 |  | T -> C | SNP (transition) | 4.0% | 1 |
| 22727 | K2_031 |  | T -> C | SNP (transition) | 24.0% | 6 |
| 22784 | K2_031 |  | C -> T | SNP (transition) | 20.0% | 5 |
| 22793 | K2_031 |  | A -> G | SNP (transition) | 20.0% | 5 |
| 22796 | K2_031 |  | G -> T | SNP (transversion) | 20.0% | 5 |
| 22821 | K2_031 |  | G -> A | SNP (transition) | 4.0% | 1 |
| 22835 | K2_031 |  | C -> T | SNP (transition) | 20.0% | 5 |
| 22860 to 22862 | K2_032 | Tailspike | TCT -> CCC | Substitution | 24.0% | 6 |
| 22881 | K2_032 | Tailspike | T -> A | SNP (transversion) | 20.0% | 5 |
| 22892 | K2_032 | Tailspike | C -> T | SNP (transition) | 20.0% | 5 |
| 22955 | K2_032 | Tailspike | T -> C | SNP (transition) | 4.0% | 1 |
| 22964 | K2_032 | Tailspike | G -> T | SNP (transversion) | 4.0% | 1 |
| 22967 | K2_032 | Tailspike | T -> C | SNP (transition) | 4.0% | 1 |
| 22988 | K2_032 | Tailspike | T -> C | SNP (transition) | 4.0% | 1 |
| 23022 | K2_032 | Tailspike | C -> T | SNP (transition) | 4.0% | 1 |
| 23044 | K2_032 | Tailspike | T -> C | SNP (transition) | 4.0% | 1 |
| 23057 | K2_032 | Tailspike | A -> G | SNP (transition) | 4.0% | 1 |
| 23060 | K2_032 | Tailspike | A -> G | SNP (transition) | 4.0% | 1 |
| 23261 | K2_032 | Tailspike | T -> C | SNP (transition) | 4.0% | 1 |
| 23264 | K2_032 | Tailspike | C -> T | SNP (transition) | 4.0% | 1 |
| 23267 | K2_032 | Tailspike | G -> C | SNP (transversion) | 4.0% | 1 |
| 23273 | K2_032 | Tailspike | G -> A | SNP (transition) | 4.0% | 1 |
| 23345 | K2_032 | Tailspike | C -> T | SNP (transition) | 4.0% | 1 |
| 23348 | K2_032 | Tailspike | C -> T | SNP (transition) | 4.0% | 1 |
| 23351 | K2_032 | Tailspike | C -> T | SNP (transition) | 4.0% | 1 |
| 23360 | K2_032 | Tailspike | C -> T | SNP (transition) | 4.0% | 1 |
| 23366 | K2_032 | Tailspike | T -> C | SNP (transition) | 4.0% | 1 |
| 23369 | K2_032 | Tailspike | C -> T | SNP (transition) | 4.0% | 1 |
| 23374 to 23375 | K2_032 | Tailspike | AT -> CC | Substitution | 4.0% | 1 |
| 23378 | K2_032 | Tailspike | C -> T | SNP (transition) | 4.0% | 1 |
| 23380 | K2_032 | Tailspike | G -> C | SNP (transversion) | 4.0% | 1 |
| 23385 to 23387 | K2_032 | Tailspike | AAC->GGT | Substitution | 4.0% | 1 |
| 23393 | K2_032 | Tailspike | C -> T | SNP (transition) | 4.0% | 1 |
| 23405 | K2_032 | Tailspike | C -> A | SNP (transversion) | 4.0% | 1 |
| 23408 | K2_032 | Tailspike | T -> G | SNP (transversion) | 4.0% | 1 |
| 23411 to 23413 | K2_032 | Tailspike | GAC->AGT | Substitution | 4.0% | 1 |
| 23415 to 23417 | K2_032 | Tailspike | CTG->TTA | Substitution | 4.0% | 1 |
| 23423 | K2_032 | Tailspike | A -> T | SNP (transversion) | 4.0% | 1 |
| 23435 | K2_032 | Tailspike | G -> T | SNP (transversion) | 4.0% | 1 |
| 23455 | K2_032 | Tailspike | A -> G | SNP (transition) | 4.0% | 1 |
| 23455 to 23456 | K2_032 | Tailspike | AC -> TG | Substitution | 4.0% | 1 |
| 23459 | K2_032 | Tailspike | T -> A | SNP (transversion) | 4.0% | 1 |
| 23462 | K2_032 | Tailspike | T -> C | SNP (transition) | 4.0% | 1 |
| 23464 to 23467 | K2_032 | Tailspike | TCAC->GGGA | Substitution | 4.0% | 1 |
| 23474 | K2_032 | Tailspike | C -> T | SNP (transition) | 4.0% | 1 |
| 23477 to 23479 | K2_032 | Tailspike | TAG->CGA | Substitution | 4.0% | 1 |
| 23486 | K2_032 | Tailspike | G -> T | SNP (transversion) | 8.0% | 2 |
| 23490 | K2_032 | Tailspike | G -> T | SNP (transversion) | 4.0% | 1 |
| 23492 | K2_032 | Tailspike | G -> A | SNP (transition) | 4.0% | 1 |
| 23493 to 23494 | K2_032 | Tailspike | GT -> AA | Substitution | 4.0% | 1 |
| 23495 | K2_032 | Tailspike | C -> T | SNP (transition) | 4.0% | 1 |
| 23496 to 23498 | K2_032 | Tailspike | GGC->CAG | Substitution | 4.0% | 1 |
| 23498 | K2_032 | Tailspike | C -> T | SNP (transition) | 4.0% | 1 |
| 23499 to 23503 | K2_032 | Tailspike | GCTTT -> CCGCA | Substitution | 4.0% | 1 |
| 23503 | K2_032 | Tailspike | T -> A | SNP (transversion) | 4.0% | 1 |
| 23508 | K2_032 | Tailspike | G -> C | SNP (transversion) | 4.0% | 1 |
| 23512 to 23516 | K2_032 | Tailspike | GTGTC -> CAAAA | Substitution | 4.0% | 1 |
| 23527 to 23540 | K2_032 | Tailspike | GGGTGATTAAACCG -> ATACTGTATATAGA | Substitution | 4.0% | 1 |
| 23542 to 23545 | K2_032 | Tailspike | GGAC->TCGA | Substitution | 4.0% | 1 |
| 23550 to 23554 | K2_032 | Tailspike | GATAA -> AACGG | Substitution | 4.0% | 1 |
| 23556 to 23558 | K2_032 | Tailspike | CAG->AAC | Substitution | 4.0% | 1 |
| 23562 to 23564 | K2_032 | Tailspike | ATA->GTG | Substitution | 4.0% | 1 |
| 23567 | K2_032 | Tailspike | A -> C | SNP (transversion) | 8.0% | 2 |
| 23568 to 23570 | K2_032 | Tailspike | GAC->AAT | Substitution | 4.0% | 1 |
| 23573 | K2_032 | Tailspike | G -> C | SNP (transversion) | 4.0% | 1 |
| 23574 | K2_032 | Tailspike | G -> T | SNP (transversion) | 4.0% | 1 |
| 23576 | K2_032 | Tailspike | A -> G | SNP (transition) | 4.0% | 1 |
| 23577 to 23583 | K2_032 | Tailspike | GCAATCG -> ACGGTGC | Substitution | 4.0% | 1 |
| 23588 to 23591 | K2_032 | Tailspike | CACA->GTCG | Substitution | 4.0% | 1 |
| 23591 | K2_032 | Tailspike | A -> C | SNP (transversion) | 4.0% | 1 |
| 23592 to 23594 | K2_032 | Tailspike | CTT->GTA | Substitution | 4.0% | 1 |
| 23596 to 23597 | K2_032 | Tailspike | AA -> CT | Substitution | 4.0% | 1 |
| 23600 to 23603 | K2_032 | Tailspike | GTCT->AAGG | Substitution | 4.0% | 1 |
| 23605 to 23612 | K2_032 | Tailspike | AAACAGAT -> TGGACAAA | Substitution | 4.0% | 1 |
| 23615 | K2_032 | Tailspike | A -> G | SNP (transition) | 4.0% | 1 |
| 23618 to 23619 | K2_032 | Tailspike | CC -> TA | Substitution | 4.0% | 1 |
| 23624 | K2_032 | Tailspike | G -> T | SNP (transversion) | 4.0% | 1 |
| 23626 to 23630 | K2_032 | Tailspike | CGGTA -> ACATT | Substitution | 4.0% | 1 |
| 23633 | K2_032 | Tailspike | C -> T | SNP (transition) | 4.0% | 1 |
| 23638 to 23639 | K2_032 | Tailspike | AC -> TG | Substitution | 4.0% | 1 |
| 23641 to 23642 | K2_032 | Tailspike | CC -> AT | Substitution | 4.0% | 1 |
| 23644 to 23645 | K2_032 | Tailspike | AG -> TC | Substitution | 4.0% | 1 |
| 23647 to 23648 | K2_032 | Tailspike | TT -> GG | Substitution | 4.0% | 1 |
| 23648 | K2_032 | Tailspike | T -> C | SNP (transition) | 4.0% | 1 |
| 23649 23651 | K2_032 | Tailspike | CCG->GAC | Substitution | 4.0% | 1 |
| 23651 | K2_032 | Tailspike | G -> T | SNP (transversion) | 4.0% | 1 |
| 23652 to 23655 | K2_032 | Tailspike | GGTA->AGCT | Substitution | 4.0% | 1 |
| 23658 to 23678 | K2_032 | Tailspike | GAATCCCTTCTCCCTCCGGAG -> CCCGAGAACATAAAAAATCAA | Substitution | 4.0% | 1 |
| 23678 | K2_032 | Tailspike | G -> A | SNP (transition) | 8.0% | 2 |
| 23679 to 23684 | K2_032 | Tailspike | GCTAAA  ->ACAGCC | Substitution | 4.0% | 1 |
| 23687 to 23689 | K2_032 | Tailspike | GCA->TGC | Substitution | 4.0% | 1 |
| 23691 | K2_032 | Tailspike | A -> G | SNP (transition) | 8.0% | 2 |
| 23692 to 23694 | K2_032 | Tailspike | GCA->CAC | Substitution | 4.0% | 1 |
| 23696 | K2_032 | Tailspike | C -> A | SNP (transversion) | 8.0% | 2 |
| 23697 to 23698 | K2_032 | Tailspike | TC -> CG | Substitution | 4.0% | 1 |
| 23700 to 23701 | K2_032 | Tailspike | TC -> GT | Substitution | 4.0% | 1 |
| 23704 to 23708 | K2_032 | Tailspike | CCCTG -> TGAGC | Substitution | 4.0% | 1 |
| 23710 | K2_032 | Tailspike | GGTC | Insertion | 4.0% | 1 |
| 23713 to 23714 | K2_032 | Tailspike | TT | Deletion | 4.0% | 1 |
| 23714 | K2_032 | Tailspike | T -> C | SNP (transition) | 4.0% | 1 |
| 23715 to 23726 | K2_032 | Tailspike | CGGGAATGTACA | Deletion | 4.0% | 1 |
| 23726 | K2_032 | Tailspike | A -> G | SNP (transition) | 4.0% | 1 |
| 23727 to 23728 | K2_032 | Tailspike | GG | Deletion | 4.0% | 1 |
| 23730 to 23732 | K2_032 | Tailspike | GTC->ATA | Substitution | 4.0% | 1 |
| 23732 | K2_032 | Tailspike | C -> T | SNP (transition) | 4.0% | 1 |
| 23733 to 23735 | K2_032 | Tailspike | GAG->ATC | Substitution | 4.0% | 1 |
| 23738 | K2_032 | Tailspike | T -> A | SNP (transversion) | 4.0% | 1 |
| 23738 | K2_032 | Tailspike | T -> C | SNP (transition) | 4.0% | 1 |
| 23739 to 23745 | K2_032 | Tailspike | CACCGGG -> AATTCCC | Substitution | 4.0% | 1 |
| 23747 | K2_032 | Tailspike | G -> A | SNP (transition) | 4.0% | 1 |
| 23748 to 23750 | K2_032 | Tailspike | AGT->GAA | Substitution | 4.0% | 1 |
| 23750 | K2_032 | Tailspike | T -> C | SNP (transition) | 4.0% | 1 |
| 23752 to 23755 | K2_032 | Tailspike | GTCT->CGAC | Substitution | 4.0% | 1 |
| 23757 to 23759 | K2_032 | Tailspike | ATG->TTC | Substitution | 4.0% | 1 |
| 23761 to 23765 | K2_032 | Tailspike | CGTGT -> GTGGC | Substitution | 4.0% | 1 |
| 23767 to 23771 | K2_032 | Tailspike | TCCTG -> ATTTA | Substitution | 4.0% | 1 |
| 23775 to 23776 | K2_032 | Tailspike | CG -> AC | Substitution | 4.0% | 1 |
| 23777 | K2_032 | Tailspike | C -> T | SNP (transition) | 4.0% | 1 |
| 23778 to 23779 | K2_032 | Tailspike | GG -> CT | Substitution | 4.0% | 1 |
| 23783 to 23784 | K2_032 | Tailspike | CC -> TA | Substitution | 4.0% | 1 |
| 23787 to 23794 | K2_032 | Tailspike | TTCTGTAA->CGTATATT | Substitution | 4.0% | 1 |
| 23796 to 23800 | K2_032 | Tailspike | ATGGT -> GTTAA | Substitution | 4.0% | 1 |
| 23802 | K2_032 | Tailspike | G -> A | SNP (transition) | 4.0% | 1 |
| 23805 to 23810 | K2_032 | Tailspike | GCTGAC -> CCACGA | Substitution | 4.0% | 1 |
| 23813 to 23816 | K2_032 | Tailspike | CCCG->TTTT | Substitution | 4.0% | 1 |
| 23818 to 23819 | K2_032 | Tailspike | GC -> TT | Substitution | 4.0% | 1 |
| 23821 to 23825 | K2_032 | Tailspike | GCGGA -> CATGG | Substitution | 4.0% | 1 |
| 23825 | K2_032 | Tailspike | A -> T | SNP (transversion) | 4.0% | 1 |
| 23826 | K2_032 | Tailspike | A -> G | SNP (transition) | 4.0% | 1 |
| 23826 to 23828 | K2_032 | Tailspike | AAA->GTG | Substitution | 4.0% | 1 |
| 23829 | K2_032 | Tailspike | G -> C | SNP (transversion) | 4.0% | 1 |
| 23829 to 23831 | K2_032 | Tailspike | GAC->TCA | Substitution | 4.0% | 1 |
| 23834 to 23837 | K2_032 | Tailspike | CGTA->AATT | Substitution | 4.0% | 1 |
| 23839 | K2_032 | Tailspike | T -> C | SNP (transition) | 4.0% | 1 |
| 23840 | K2_032 | Tailspike | T -> C | SNP (transition) | 4.0% | 1 |
| 23841 to 23842 | K2_032 | Tailspike | AC -> TT | Substitution | 4.0% | 1 |
| 23844 to 23846 | K2_032 | Tailspike | TTT->GAA | Substitution | 4.0% | 1 |
| 23846 | K2_032 | Tailspike | T -> C | SNP (transition) | 4.0% | 1 |
| 23847 to 23850 | K2_032 | Tailspike | GAAA->AACC | Substitution | 4.0% | 1 |
| 23853 to 23855 | K2_032 | Tailspike | CTG->TTA | Substitution | 4.0% | 1 |
| 23854 to 23855 | K2_032 | Tailspike | TG -> AT | Substitution | 4.0% | 1 |
| 23857 to 23860 | K2_032 | Tailspike | GCGG->CATC | Substitution | 4.0% | 1 |
| 23861 | K2_032 | Tailspike | C -> A | SNP (transversion) | 4.0% | 1 |
| 23863 to 23864 | K2_032 | Tailspike | AT -> CA | Substitution | 4.0% | 1 |
| 23870 | K2_032 | Tailspike | C -> T | SNP (transition) | 8.0% | 2 |
| 23871 to 23873 | K2_032 | Tailspike | AAG->TAT | Substitution | 4.0% | 1 |
| 23873 | K2_032 | Tailspike | G -> A | SNP (transition) | 4.0% | 1 |
| 23876 | K2_032 | Tailspike | T -> G | SNP (transversion) | 4.0% | 1 |
| 23881 to 23882 | K2_032 | Tailspike | AT -> GG | Substitution | 4.0% | 1 |
| 23885 | K2_032 | Tailspike | T -> C | SNP (transition) | 4.0% | 1 |
| 23886 to 23888 | K2_032 | Tailspike | ATT->GTA | Substitution | 4.0% | 1 |
| 23891 | K2_032 | Tailspike | T -> A | SNP (transversion) | 4.0% | 1 |
| 23894 | K2_032 | Tailspike | G -> T | SNP (transversion) | 4.0% | 1 |
| 23895 to 23897 | K2_032 | Tailspike | CGC->AGA | Substitution | 4.0% | 1 |
| 23895 to 23897 | K2_032 | Tailspike | CGC->GAG | Substitution | 4.0% | 1 |
| 23899 | K2_032 | Tailspike | C -> T | SNP (transition) | 4.0% | 1 |
| 23900 | K2_032 | Tailspike | A -> T | SNP (transversion) | 4.0% | 1 |
| 23902 to 23903 | K2_032 | Tailspike | GT -> AG | Substitution | 4.0% | 1 |
| 23903 | K2_032 | Tailspike | T -> C | SNP (transition) | 4.0% | 1 |
| 23906 | K2_032 | Tailspike | C -> T | SNP (transition) | 8.0% | 2 |
| 23909 | K2_032 | Tailspike | T -> G | SNP (transversion) | 4.0% | 1 |
| 23912 | K2_032 | Tailspike | G -> A | SNP (transition) | 8.0% | 2 |
| 23914 to 23917 | K2_032 | Tailspike | TAAG->GGTC | Substitution | 4.0% | 1 |
| 23919 to 23920 | K2_032 | Tailspike | AG -> GC | Substitution | 4.0% | 1 |
| 23921 | K2_032 | Tailspike | C -> T | SNP (transition) | 4.0% | 1 |
| 23923 | K2_032 | Tailspike | C -> T | SNP (transition) | 4.0% | 1 |
| 23925 to 23927 | K2_032 | Tailspike | CAA->TTG | Substitution | 4.0% | 1 |
| 23927 | K2_032 | Tailspike | A -> G | SNP (transition) | 4.0% | 1 |
| 23930 | K2_032 | Tailspike | C -> T | SNP (transition) | 8.0% | 2 |
| 23931 to 23933 | K2_032 | Tailspike | TTA -> ATT | Substitution | 4.0% | 1 |
| 23936 | K2_032 | Tailspike | A -> C | SNP (transversion) | 4.0% | 1 |
| 23936 | K2_032 | Tailspike | A -> T | SNP (transversion) | 4.0% | 1 |
| 23939 | K2_032 | Tailspike | C -> T | SNP (transition) | 4.0% | 1 |
| 23940 to 23942 | K2_032 | Tailspike | AAT->GAC | Substitution | 4.0% | 1 |
| 23949 to 23951 | K2_032 | Tailspike | TTC->GAT | Substitution | 4.0% | 1 |
| 23953 to 23954 | K2_032 | Tailspike | CG -> AA | Substitution | 4.0% | 1 |
| 23953 to 23954 | K2_032 | Tailspike | CG -> AC | Substitution | 4.0% | 1 |
| 23956 to 23957 | K2_032 | Tailspike | GC -> AT | Substitution | 4.0% | 1 |
| 23963 | K2_032 | Tailspike | C -> A | SNP (transversion) | 8.0% | 2 |
| 23969 | K2_032 | Tailspike | C -> G | SNP (transversion) | 4.0% | 1 |
| 23969 | K2_032 | Tailspike | C -> T | SNP (transition) | 4.0% | 1 |
| 23971 to 23972 | K2_032 | Tailspike | TC -> GA | Substitution | 4.0% | 1 |
| 23972 | K2_032 | Tailspike | C -> T | SNP (transition) | 4.0% | 1 |
| 23974 to 23976 | K2_032 | Tailspike | GGT->ACC | Substitution | 4.0% | 1 |
| 23980 to 23981 | K2_032 | Tailspike | CT -> TA | Substitution | 4.0% | 1 |
| 23981 | K2_032 | Tailspike | T -> C | SNP (transition) | 4.0% | 1 |
| 23984 | K2_032 | Tailspike | G -> A | SNP (transition) | 4.0% | 1 |
| 23984 | K2_032 | Tailspike | G -> T | SNP (transversion) | 4.0% | 1 |
| 23987 | K2_032 | Tailspike | T -> C | SNP (transition) | 4.0% | 1 |
| 23992 to 23993 | K2_032 | Tailspike | CG -> TT | Substitution | 4.0% | 1 |
| 23993 | K2_032 | Tailspike | G -> T | SNP (transversion) | 4.0% | 1 |
| 23998 | K2_032 | Tailspike | A -> G | SNP (transition) | 4.0% | 1 |
| 24002 | K2_032 | Tailspike | T -> C | SNP (transition) | 8.0% | 2 |
| 24005 | K2_032 | Tailspike | T -> G | SNP (transversion) | 4.0% | 1 |
| 24008 | K2_032 | Tailspike | T -> A | SNP (transversion) | 4.0% | 1 |
| 24011 | K2_032 | Tailspike | G -> A | SNP (transition) | 4.0% | 1 |
| 24014 | K2_032 | Tailspike | G -> T | SNP (transversion) | 4.0% | 1 |
| 24016 to 24017 | K2_032 | Tailspike | GG -> AC | Substitution | 4.0% | 1 |
| 24020 | K2_032 | Tailspike | A -> G | SNP (transition) | 4.0% | 1 |
| 24020 | K2_032 | Tailspike | A | Deletion | 4.0% | 1 |
| 24021 to 24023 | K2_032 | Tailspike | GGT -> GA | Deletion | 4.0% | 1 |
| 24026 | K2_032 | Tailspike | G -> A | SNP (transition) | 4.0% | 1 |
| 24027 | K2_032 | Tailspike | G -> AAA | Insertion | 4.0% | 1 |
| 24032 to 24035 | K2_032 | Tailspike | TTCT->CGGC | Substitution | 4.0% | 1 |
| 24037 | K2_032 | Tailspike | C -> G | SNP (transversion) | 4.0% | 1 |
| 24039 | K2_032 | Tailspike | A -> T | SNP (transversion) | 4.0% | 1 |
| 24042 to 24044 | K2_032 | Tailspike | TCT->GCC | Substitution | 4.0% | 1 |
| 24047 | K2_032 | Tailspike | T -> G | SNP (transversion) | 4.0% | 1 |
| 24054 to 24055 | K2_032 | Tailspike | AA -> CG | Substitution | 4.0% | 1 |
| 24060 to 24061 | K2_032 | Tailspike | CA -> GT | Substitution | 4.0% | 1 |
| 24066 to 24069 | K2_032 | Tailspike | CGGG->GATA | Substitution | 4.0% | 1 |
| 24072 to 24081 | K2_032 | Tailspike | TCAGCAGTGC -> ATTACGACCA | Substitution | 4.0% | 1 |
| 24084 to 24092 | K2_032 | Tailspike | TACCCTGTA  -> CAGTGCTAT | Substitution | 4.0% | 1 |
| 24094 to 24095 | K2_032 | Tailspike | GG -> AC | Substitution | 4.0% | 1 |
| 24101 to 24104 | K2_032 | Tailspike | CTTC-> GATA | Substitution | 4.0% | 1 |
| 24108 to 24112 | K2_032 | Tailspike | TTAGG -> GTCAA | Substitution | 4.0% | 1 |
| 24122 | K2_032 | Tailspike | T -> C | SNP (transition) | 4.0% | 1 |
| 24124 to 24134 | K2_032 | Tailspike | ACACGAACCCC -> GTTCCCCGGTT | Substitution | 4.0% | 1 |
| 24127 | K2_032 | Tailspike | C -> T | SNP (transition) | 92.0% | 23 |
| 24137 to 24139 | K2_032 | Tailspike | AGA->GCG | Substitution | 4.0% | 1 |
| 24142 to 24148 | K2_032 | Tailspike | ACCGCCC->TAGATGATT | Insertion | 4.0% | 1 |
| 24150 to 24154 | K2_032 | Tailspike | GGGGA -> CCCAC | Substitution | 4.0% | 1 |
| 24161 | K2_032 | Tailspike | C -> A | SNP (transversion) | 4.0% | 1 |
| 24164 | K2_032 | Tailspike | T -> A | SNP (transversion) | 4.0% | 1 |
| 24167 to 24169 | K2_032 | Tailspike | TCA->CGT | Substitution | 4.0% | 1 |
| 24171 to 24176 | K2_032 | Tailspike | TACCCG -> GTTTCA | Substitution | 4.0% | 1 |
| 24179 to 24189 | K2_032 | Tailspike | ACATATGCTCC -> TGCCGACTAAA | Substitution | 4.0% | 1 |
| 24192 to 24194 | K2_032 | Tailspike | TTA->CAT | Substitution | 4.0% | 1 |
| 24197 to 24214 | K2_032 | Tailspike | CCATTTGATAGACAATCT -> TTCGCAACATAATCACGA | Substitution | 4.0% | 1 |
| 24216 to 24224 | K2_032 | Tailspike | TTTGTTAGA-> GGATTGCAT | Substitution | 4.0% | 1 |
| 24227 | K2_032 | Tailspike | T -> G | SNP (transversion) | 4.0% | 1 |
| 24229 to 24232 | K2_032 | Tailspike | CGCT-> ATTG | Substitution | 4.0% | 1 |
| 24234 to 24239 | K2_032 | Tailspike | GGGGTA -> TGCCTG | Substitution | 4.0% | 1 |
| 24241 to 24251 | K2_032 | Tailspike | GTTTCGGTATG -> TGGGATGGTCA | Substitution | 4.0% | 1 |
| 24254 to 24262 | K2_032 | Tailspike | CGGGCAAGG -> ATAATATCA | Substitution | 4.0% | 1 |
| 24264 to 24266 | K2_032 | Tailspike | CTG->AGA | Substitution | 4.0% | 1 |
| 24269 to 24272 | K2_032 | Tailspike | TGTC ->ATAT | Substitution | 4.0% | 1 |
| 24274 | K2_032 | Tailspike | C -> G | SNP (transversion) | 4.0% | 1 |
| 24276 | K2_032 | Tailspike | A -> G | SNP (transition) | 4.0% | 1 |
| 24282 to 24284 | K2_032 | Tailspike | ACT->TGA | Substitution | 4.0% | 1 |
| 24286 | K2_032 | Tailspike | T -> G | SNP (transversion) | 4.0% | 1 |
| 24288 to 24296 | K2_032 | Tailspike | GAGGATTGC -> CCACAAAGA | Substitution | 4.0% | 1 |
| 24298 to 24299 | K2_032 | Tailspike | CT -> GG | Substitution | 4.0% | 1 |
| 24301 | K2_032 | Tailspike | G -> A | SNP (transition) | 4.0% | 1 |
| 24304 | K2_032 | Tailspike | C -> T | SNP (transition) | 4.0% | 1 |
| 24306 to 24317 | K2_032 | Tailspike | GGGGCTTATATT -> TGATAGAGGTA | Deletion | 4.0% | 1 |
| 24320 to 24328 | K2_032 | Tailspike | TGCCCACGA -> AACAATGAT | Substitution | 4.0% | 1 |
| 24330 | K2_032 | Tailspike | A -> T | SNP (transversion) | 4.0% | 1 |
| 24333 | K2_032 | Tailspike | G -> C | SNP (transversion) | 4.0% | 1 |
| 24336 to 24338 | K2_032 | Tailspike | TTC->ACGTA | Insertion | 4.0% | 1 |
| 24341 to 24343 | K2_032 | Tailspike | TAA->GGT | Substitution | 4.0% | 1 |
| 24347 | K2_032 | Tailspike | C -> A | SNP (transversion) | 4.0% | 1 |
| 24349 to 24353 | K2_032 | Tailspike | CAATA -> GTGCG | Substitution | 4.0% | 1 |
| 24355 to 24357 | K2_032 | Tailspike | TCG | Deletion | 4.0% | 1 |
| 24361 to 24363 | K2_032 | Tailspike | CCA->AGG | Substitution | 4.0% | 1 |
| 24366 to 24368 | K2_032 | Tailspike | ACT->TTA | Substitution | 4.0% | 1 |
| 24370 to 24371 | K2_032 | Tailspike | AA -> CT | Substitution | 4.0% | 1 |
| 24375 to 24382 | K2_032 | Tailspike | TTCCCCGC  -> CTGAATCA | Substitution | 4.0% | 1 |
| 24384 to 24397 | K2_032 | Tailspike | AACCAGATATATAT -> CTTACCTGCGAAGG | Substitution | 4.0% | 1 |
| 24399 to 24407 | K2_032 | Tailspike | TCGGGGGCC -> AGTAGCCGA | Substitution | 4.0% | 1 |
| 24409 to 24410 | K2_032 | Tailspike | GT -> TG | Substitution | 4.0% | 1 |
| 24413 | K2_032 | Tailspike | T -> G | SNP (transversion) | 4.0% | 1 |
| 24415 to 24423 | K2_032 | Tailspike | TAAACGGCC  -> GCGTGTTGA | Substitution | 4.0% | 1 |
| 24427 | K2_032 | Tailspike | G -> A | SNP (transition) | 4.0% | 1 |
| 24429 to 24430 | K2_032 | Tailspike | TT -> GC | Substitution | 4.0% | 1 |
| 24432 to 24436 | K2_032 | Tailspike | GTCGG -> TATAC | Substitution | 4.0% | 1 |
| 24439 to 24445 | K2_032 | Tailspike | TCCGTTC -> CAGAAGG | Substitution | 4.0% | 1 |
| 24447 to 24448 | K2_032 | Tailspike | AC -> TA | Substitution | 4.0% | 1 |
| 24450 | K2_032 | Tailspike | A -> G | SNP (transition) | 4.0% | 1 |
| 24454 to 24458 | K2_032 | Tailspike | AACAG -> TGTAT | Substitution | 4.0% | 1 |
| 24460 to 24470 | K2_032 | Tailspike | GCATGACGATA -> CACCACAATCG | Substitution | 4.0% | 1 |
| 24473 to 24475 | K2_032 | Tailspike | CGC->AAT | Substitution | 4.0% | 1 |
| 24477 to 24484 | K2_032 | Tailspike | CCTAACTC->TCCGCTGT | Substitution | 4.0% | 1 |
| 24486 | K2_032 | Tailspike | A -> G | SNP (transition) | 4.0% | 1 |
| 24489 to 24493 | K2_032 | Tailspike | GTAAG -> TGCGC | Substitution | 4.0% | 1 |
| 24497 | K2_032 | Tailspike | A -> G | SNP (transition) | 4.0% | 2 |
| 24497 | K2_032 | Tailspike | A -> T | SNP (transversion) | 8.0% | 1 |
| 24498 to 24499 | K2_032 | Tailspike | AT -> TC | Substitution | 4.0% | 1 |
| 24501 to 24511 | K2_032 | Tailspike | ACGGGCTTCGT-> GGTACGAAAAA | Substitution | 4.0% | 1 |
| 24513 to 24515 | K2_032 | Tailspike | GAC->ATA | Substitution | 4.0% | 1 |
| 24517 to 24518 | K2_032 | Tailspike | CC -> TT | Substitution | 4.0% | 1 |
| 24520 to 24521 | K2_032 | Tailspike | CA -> GT | Substitution | 4.0% | 1 |
| 24523 to 24526 | K2_032 | Tailspike | GGAT->CCTA | Substitution | 4.0% | 1 |
| 24529 to 24530 | K2_032 | Tailspike | AC -> TT | Substitution | 4.0% | 1 |
| 24532 to 24533 | K2_032 | Tailspike | TA -> GT | Substitution | 4.0% | 1 |
| 24535 to 24541 | K2_032 | Tailspike | CCAATTT -> ATGTACA | Substitution | 4.0% | 1 |
| 24543 to 24545 | K2_032 | Tailspike | ATG->GGT | Substitution | 4.0% | 1 |
| 24547 to 24553 | K2_032 | Tailspike | AGGAAGG -> GCAACAT | Substitution | 4.0% | 1 |
| 24555 to 24556 | K2_032 | Tailspike | CT -> AA | Substitution | 4.0% | 1 |
| 24559 to 24565 | K2_032 | Tailspike | GTAACTC -> TCCAGCA | Substitution | 4.0% | 1 |
| 24567 to 24568 | K2_032 | Tailspike | CG -> AA | Substitution | 4.0% | 1 |
| 24570 to 24571 | K2_032 | Tailspike | AT -> GA | Substitution | 4.0% | 1 |
| 24576 to 24581 | K2_032 | Tailspike | AGTTTC -> CAGATG | Substitution | 4.0% | 1 |
| 24583 to 24586 | K2_032 | Tailspike | ATAA->CCCT | Substitution | 4.0% | 1 |
| 24589 | K2_032 | Tailspike | A -> C | SNP (transversion) | 4.0% | 1 |
| 24589 | K2_032 | Tailspike | A -> G | SNP (transition) | 4.0% | 1 |
| 24591 to 24604 | K2_032 | Tailspike | TCTGCGGCGCTTCG -> ATGCGTCCTGCGAT | Substitution | 4.0% | 1 |
| 24606 to 24611 | K2_032 | Tailspike | TTTCGC -> GGCGGT | Substitution | 4.0% | 1 |
| 24613 | K2_032 | Tailspike | T -> C | SNP (transition) | 4.0% | 1 |
| 24615 to 24617 | K2_032 | Tailspike | CAT->ATA | Substitution | 4.0% | 1 |
| 24620 | K2_032 | Tailspike | A -> T | SNP (transversion) | 4.0% | 1 |
| 24622 to 24623 | K2_032 | Tailspike | TG -> CA | Substitution | 4.0% | 1 |
| 24626 to 24628 | K2_032 | Tailspike | AAA->GCT | Substitution | 4.0% | 1 |
| 24630 to 24641 | K2_032 | Tailspike | ACCCTTGATAGT -> GTAATGACCGCA | Substitution | 4.0% | 1 |
| 24643 to 24644 | K2_032 | Tailspike | GG -> AT | Substitution | 4.0% | 1 |
| 24646 | K2_032 | Tailspike | C -> G | SNP (transversion) | 4.0% | 1 |
| 24648 to 24654 | K2_032 | Tailspike | GTGTACT->CAAGTTG | Substitution | 4.0% | 1 |
| 24658 to 24661 | K2_032 | Tailspike | ACAT->TACC | Substitution | 4.0% | 1 |
| 24663 to 24665 | K2_032 | Tailspike | AAC->GGG | Substitution | 4.0% | 1 |
| 24668 | K2_032 | Tailspike | C -> T | SNP (transition) | 4.0% | 1 |
| 24670 to 24672 | K2_032 | Tailspike | GCC->AAG | Substitution | 4.0% | 1 |
| 24675 | K2_032 | Tailspike | G -> A | SNP (transition) | 4.0% | 1 |
| 24678 to 24680 | K2_032 | Tailspike | TCC -> ATT | Substitution | 4.0% | 1 |
| 24682 to 24689 | K2_032 | Tailspike | GCTCAGCC->TTAAGCTT | Substitution | 4.0% | 1 |
| 2469 to 24697 | K2_032 | Tailspike | GGACCGA -> CAGCGAT | Substitution | 4.0% | 1 |
| 24699 | K2_032 | Tailspike | ATTACC -> CAGGAT | Substitution | 4.0% | 1 |
| 24706 to 24704 | K2_032 | Tailspike | C -> G | SNP (transversion) | 4.0% | 1 |
| 24708 to 24713 | K2_032 | Tailspike | ATTGCG -> GTACGA | Substitution | 4.0% | 1 |
| 24715 to 24721 | K2_032 | Tailspike | GGTCATT->TAGGTGA | Substitution | 4.0% | 1 |
| 24724 to 24737 | K2_032 | Tailspike | CGGATGCCGTGTCG -> TCCAACTTAACCGC | Substitution | 4.0% | 1 |
| 24741 to 24746 | K2_032 | Tailspike | AAAATA -> GGTTTT | Substitution | 4.0% | 1 |
| 24749 to 24761 | K2_032 | Tailspike | CAGGGGCGATTAT -> ACATATGAGCATA | Substitution | 4.0% | 1 |
| 24763 to 24764 | K2_032 | Tailspike | GC -> CA | Substitution | 4.0% | 1 |
| 24766 to 24776 | K2_032 | Tailspike | CTGTTGAGATA -> TATCCCCATCG | Substitution | 4.0% | 1 |
| 24778 to 24780 | K2_032 | Tailspike | CTG->AAT | Substitution | 4.0% | 1 |
| 24783 | K2_032 | Tailspike | T -> C | SNP (transition) | 4.0% | 1 |
| 24787 to 24791 | K2_032 | Tailspike | TGTCC -> ATAGT | Substitution | 4.0% | 1 |
| 24793 to 24799 | K2_032 | Tailspike | TCCTACC -> CATTGGA | Substitution | 4.0% | 1 |
| 24801 to 24803 | K2_032 | Tailspike | GAC->CAT | Substitution | 4.0% | 1 |
| 24807 to 24811 | K2_032 | Tailspike | GCTGT -> TCATC | Substitution | 4.0% | 1 |
| 24814 to 24815 | K2_032 | Tailspike | GG -> TA | Substitution | 4.0% | 1 |
| 24817 to 24820 | K2_032 | Tailspike | ATAA->GCTT | Substitution | 4.0% | 1 |
| 24822 to 24824 | K2_032 | Tailspike | GGG->TTC | Substitution | 4.0% | 1 |
| 24826 | K2_032 | Tailspike | C -> T | SNP (transition) | 4.0% | 1 |
| 24828 to 24829 | K2_032 | Tailspike | AT -> GG | Substitution | 4.0% | 1 |
| 24831 to 24841 | K2_032 | Tailspike | TCACTGTATCT -> AGTGACGG | Deletion | 4.0% | 1 |
| 24844 | K2_032 | Tailspike | A -> T | SNP (transversion) | 4.0% | 1 |
| 24846 to 24851 | K2_032 | Tailspike | GGCGAT -> TTGCGA | Substitution | 4.0% | 1 |
| 24853 | K2_032 | Tailspike | G -> T | SNP (transversion) | 4.0% | 1 |
| 24857 to 24862 | K2_032 | Tailspike | TAAGGC -> CGCCAA | Substitution | 4.0% | 1 |
| 24864 to 24865 | K2_032 | Tailspike | TT -> AA | Substitution | 4.0% | 1 |
| 24867 to 24873 | K2_032 | Tailspike | GTTAAGC -> CCAGACG | Substitution | 4.0% | 1 |
| 24878 to 24881 | K2_032 | Tailspike | CGAT->TTAC | Substitution | 4.0% | 1 |
| 24883 | K2_032 | Tailspike | G -> T | SNP (transversion) | 4.0% | 1 |
| 24886 | K2_032 | Tailspike | G -> C | SNP (transversion) | 4.0% | 1 |
| 24893 to 24895 | K2_032 | Tailspike | AAG->CCT | Substitution | 4.0% | 1 |
| 24898 to 24908 | K2_032 | Tailspike | TAACTTTGGCA -> CC | Deletion | 4.0% | 1 |
| 24910 | K2_032 | Tailspike | A -> G | SNP (transition) | 4.0% | 1 |
| 24928 |  |  | A -> T | SNP (transversion) | 4.0% | 1 |
| 24930 |  |  | T | Insertion | 4.0% | 1 |
| 24964 |  |  | C -> T | SNP (transition) | 4.0% | 1 |
| 24970 |  |  | G -> A | SNP (transition) | 4.0% | 1 |
| 24979 |  |  | A -> C | SNP (transversion) | 4.0% | 1 |
| 24981 to 24982 |  |  | AA -> GC | Substitution | 4.0% | 1 |
| 25001 |  |  | C -> T | SNP (transition) | 8.0% | 2 |
| 25007 |  |  | G -> A | SNP (transition) | 8.0% | 2 |
| 25019 |  |  | C -> A | SNP (transversion) | 8.0% | 2 |
| 25035 | K2_033 |  | T -> C | SNP (transition) | 8.0% | 2 |
| 25038 to 25039 | K2_033 |  | CG -> TT | Substitution | 8.0% | 2 |
| 25044 to 25045 | K2_033 |  | TC -> GG | Substitution | 8.0% | 2 |
| 25053 to 25054 | K2_033 |  | CA -> TT | Substitution | 8.0% | 2 |
| 25060 to 25061 | K2_033 |  | CG -> AA | Substitution | 8.0% | 2 |
| 25064 to 25074 | K2_033 |  | TGTTCCAATAA -> AT | Deletion | 8.0% | 2 |
| 26744 | K2_034 | DNA helicase | C -> T | SNP (transition) | 20.0% | 5 |
| 26842 | K2_034 | DNA helicase | A -> G | SNP (transition) | 40.0% | 10 |
| 27093 | K2_034 | DNA helicase | G -> A | SNP (transition) | 20.0% | 5 |
| 27215 | K2_034 | DNA helicase | T -> C | SNP (transition) | 20.0% | 5 |
| 27476 | K2_034 | DNA helicase | G -> A | SNP (transition) | 4.0% | 1 |
| 27620 to 27621 | K2_034 | DNA helicase | CT -> AA | Substitution | 16.0% | 4 |
| 27627 |  |  | C -> G | SNP (transversion) | 16.0% | 4 |
| 27639 to 27641 |  |  | CTC->AGG | Substitution | 16.0% | 4 |
| 27645 |  |  | G -> T | SNP (transversion) | 16.0% | 4 |
| 27647 to 27648 |  |  | GG -> TA | Substitution | 16.0% | 4 |
| 27650 to 27651 |  |  | TA -> CG | Substitution | 16.0% | 4 |
| 27654 |  |  | C -> T | SNP (transition) | 16.0% | 4 |
| 27665 to 27667 | K2_035 |  | CAG->TAC | Substitution | 16.0% | 4 |
| 27674 | K2_035 |  | T -> C | SNP (transition) | 16.0% | 4 |
| 27677 | K2_035 |  | A -> C | SNP (transversion) | 16.0% | 4 |
| 27680 | K2_035 |  | C -> T | SNP (transition) | 16.0% | 4 |
| 27683 | K2_035 |  | T -> C | SNP (transition) | 16.0% | 4 |
| 27686 | K2_035 |  | A -> C | SNP (transversion) | 16.0% | 4 |
| 27701 to 27704 | K2_035 |  | AACG->GGTA | Substitution | 16.0% | 4 |
| 27710 | K2_035 |  | C -> G | SNP (transversion) | 24.0% | 6 |
| 27716 | K2_035 |  | A -> G | SNP (transition) | 16.0% | 4 |
| 27722 | K2_035 |  | G -> A | SNP (transition) | 16.0% | 4 |
| 27728 | K2_035 |  | C -> A | SNP (transversion) | 24.0% | 6 |
| 27803 | K2_035 |  | A -> G | SNP (transition) | 8.0% | 2 |
| 28325 | K2_037 | HNH motif endonuclease | A -> C | SNP (transversion) | 4.0% | 1 |
| 28434 | K2_037 | HNH motif endonuclease | TAATATGGT | Insertion | 4.0% | 1 |
| 28541 | K2_037 | HNH motif endonuclease | C -> T | SNP (transition) | 16.0% | 4 |
| 28908 | K2_038 | DNA pol I | A -> C | SNP (transversion) | 4.0% | 1 |
| 29502 | K2_038 | DNA pol I | T -> C | SNP (transition) | 16.0% | 4 |
| 29936 | K2_038 | DNA pol I | T -> G | SNP (transversion) | 4.0% | 1 |
| 31771 | K2_041 | RecB-like nuclease | T -> C | SNP (transition) | 40.0% | 10 |
| 32458 | K2_041 | RecB-like nuclease | T -> C | SNP (transition) | 16.0% | 4 |
| 33660 |  |  | (T)6 -> (T)7 | Insertion (TR) | 12.0% | 3 |
| 34722 | K2_044 | Helicase, repA | A -> C | SNP (transversion) | 4.0% | 1 |
| 35969 | K2_044 | Helicase, repA | A -> G | SNP (transition) | 12.0% | 3 |
| 37454 | K2_048 |  | C -> T | SNP (transition) | 12.0% | 3 |
| 37667 | K2_049 |  | A -> G | SNP (transition) | 28.0% | 7 |
| 37786 | K2_049 |  | T -> A | SNP (transversion) | 4.0% | 1 |
| 38099 |  |  | T -> C | SNP (transition) | 12.0% | 3 |
| 38193 | K2_051 |  | G -> A | SNP (transition) | 8.0% | 2 |
| 38284 | K2_052 |  | T -> C | SNP (transition) | 12.0% | 3 |
| 38495 | K2_053 |  | A -> G | SNP (transition) | 8.0% | 2 |
| 38533 | K2_053 |  | C -> T | SNP (transition) | 8.0% | 2 |
| 38596 | K2_053 |  | G -> A | SNP (transition) | 8.0% | 2 |
| 38835 | K2_053 |  | C -> T | SNP (transition) | 8.0% | 2 |
| 39285 | K2_055 |  | T -> G | SNP (transversion) | 4.0% | 1 |
| 39849 | K2_056 |  | G -> A | SNP (transition) | 4.0% | 1 |
| 40573 | K2_058 |  | T -> A | SNP (transversion) | 20.0% | 5 |
| 41128 | K2_059 | Phage lysin | A -> G | SNP (transition) | 4.0% | 1 |
| 41240 |  |  | (T)8 -> (T)9 | Insertion (TR) | 8.0% | 2 |
| 41240 |  |  | (T)8 -> (T)9 | Insertion (TR) | 32.0% | 8 |
| 41250 |  |  | C -> A | SNP (transversion) | 32.0% | 8 |
| 41271 |  |  | G -> A | SNP (transition) | 32.0% | 8 |
| 41379 | K2_060 |  | G -> A | SNP (transition) | 4.0% | 1 |
| 41385 | K2_060 |  | C -> T | SNP (transition) | 4.0% | 1 |
| 41398 | K2_060 |  | T -> C | SNP (transition) | 4.0% | 1 |
| 41406 | K2_060 |  | C -> A | SNP (transversion) | 4.0% | 1 |
| 41415 | K2_060 |  | T -> C | SNP (transition) | 4.0% | 1 |
| 41434 | K2_060 |  | A -> C | SNP (transversion) | 4.0% | 1 |
| 41439 | K2_060 |  | A -> T | SNP (transversion) | 4.0% | 1 |
| 41442 | K2_060 |  | A -> C | SNP (transversion) | 4.0% | 1 |
| 41445 | K2_060 |  | G -> C | SNP (transversion) | 4.0% | 1 |
| 41448 | K2_060 |  | T -> C | SNP (transition) | 4.0% | 1 |
| 41451 | K2_060 |  | T -> G | SNP (transversion) | 4.0% | 1 |
| 41478 | K2_060 |  | G -> T | SNP (transversion) | 4.0% | 1 |
| 41484 | K2_060 |  | A -> C | SNP (transversion) | 4.0% | 1 |
| 41615 | K2_061 |  | C -> T | SNP (transition) | 4.0% | 1 |
| 41619 | K2_061 |  | C -> A | SNP (transversion) | 4.0% | 1 |
| 41627 | K2_061 |  | G -> A | SNP (transition) | 4.0% | 1 |
| 41760 | K2_062 |  | G -> A | SNP (transition) | 20.0% | 5 |
| 41872 to 41873 | K2_063 |  | GT -> AG | Substitution | 36.0% | 9 |
| 41886 to 41887 | K2_063 |  | AA -> GG | Substitution | 36.0% | 9 |
| 41890 | K2_063 |  | C -> T | SNP (transition) | 36.0% | 9 |
| 41902 | K2_063 |  | A -> T | SNP (transversion) | 36.0% | 9 |
| 41950 | K2_063 |  | C -> T | SNP (transition) | 36.0% | 9 |
| 41953 to 41954 | K2_063 |  | AC -> GT | Substitution | 36.0% | 9 |
| 41971 | K2_063 |  | T -> C | SNP (transition) | 36.0% | 9 |
| 41977 | K2_063 |  | C -> T | SNP (transition) | 36.0% | 9 |
| 41980 | K2_063 |  | C -> T | SNP (transition) | 36.0% | 9 |
| 41996 to 41998 | K2_063 |  | GGA->ACG | Substitution | 36.0% | 9 |
| 42001 | K2_063 |  | T -> C | SNP (transition) | 36.0% | 9 |
| 4200 to 42010 | K2_063 |  | TC -> AT | Substitution | 36.0% | 9 |
| 42013 | K2_063 |  | T -> C | SNP (transition) | 36.0% | 9 |
| 42016 | K2_063 |  | C -> T | SNP (transition) | 36.0% | 9 |
| 42018 | K2_063 |  | A -> C | SNP (transversion) | 36.0% | 9 |
| 42021 | K2_063 |  | G -> A | SNP (transition) | 36.0% | 9 |
| 42027 to 42033 | K2_063 |  | AATGTTT->TGAGCGG | Substitution | 36.0% | 9 |
| 42035 to 42037 | K2_063 |  | GAT->CAG | Substitution | 36.0% | 9 |
| 42040 | K2_063 |  | C -> T | SNP (transition) | 36.0% | 9 |
| 42046 | K2_063 |  | A -> G | SNP (transition) | 36.0% | 9 |
| 42049 | K2_063 |  | T -> C | SNP (transition) | 36.0% | 9 |
| 42052 | K2_063 |  | GGCGATTGAACG | Insertion | 36.0% | 9 |
| 42055 | K2_063 |  | G -> A | SNP (transition) | 36.0% | 9 |
| 42057 to 42058 | K2_063 |  | GT -> AC | Substitution | 36.0% | 9 |
| 42062 | K2_063 |  | A -> T | SNP (transversion) | 36.0% | 9 |
| 42066 | K2_063 |  | +C | Insertion | 36.0% | 9 |
| 42068 | K2_063 |  | T -> A | SNP (transversion) | 36.0% | 9 |
| 42068 | K2_064 |  | T -> A | SNP (transversion) | 36.0% | 9 |
| 42075 | K2_064 |  | G -> A | SNP (transition) | 36.0% | 9 |
| 42079 to 42081 | K2_064 |  | TGT->CGC | Substitution | 36.0% | 9 |
| 42087 | K2_064 |  | G -> A | SNP (transition) | 36.0% | 9 |
| 42096 | K2_064 |  | T -> C | SNP (transition) | 36.0% | 9 |
| 42099 | K2_064 |  | G -> A | SNP (transition) | 36.0% | 9 |
| 42102 | K2_064 |  | A -> C | SNP (transversion) | 36.0% | 9 |
| 42105 | K2_064 |  | T -> C | SNP (transition) | 36.0% | 9 |
| 42107 | K2_064 |  | T -> C | SNP (transition) | 36.0% | 9 |
| 42112 | K2_064 |  | C -> T | SNP (transition) | 36.0% | 9 |
| 42119 to 42120 | K2_064 |  | CA -> AT | Substitution | 36.0% | 9 |
| 42123 | K2_064 |  | C -> A | SNP (transversion) | 36.0% | 9 |
| 42130 to 42132 | K2_064 |  | TTA->CTG | Substitution | 36.0% | 9 |
| 42135 to 42136 | K2_064 |  | TT -> CC | Substitution | 36.0% | 9 |
| 42146 | K2_064 |  | G -> A | SNP (transition) | 36.0% | 9 |
| 42168 | K2_064 |  | G -> A | SNP (transition) | 36.0% | 9 |
| 42180 | K2_064 |  | A -> G | SNP (transition) | 4.0% | 1 |
| 42182 | K2_064 |  | T -> C | SNP (transition) | 4.0% | 1 |
| 42185 to 42187 | K2_064 |  | ACC->CTA | Substitution | 4.0% | 1 |
| 42207 to 42210 | K2_064 |  | TAAA->ACAG | Substitution | 4.0% | 1 |
| 42230 | K2_065 |  | C -> A | SNP (transversion) | 4.0% | 1 |
| 42233 | K2_065 |  | G -> A | SNP (transition) | 4.0% | 1 |
| 42245 | K2_065 |  | C -> A | SNP (transversion) | 4.0% | 1 |
| 42260 | K2_065 |  | G -> A | SNP (transition) | 4.0% | 1 |

* All the half values (0.5 etc.) are due to imprecise base calling during Illumina sequencing. MCP, Major Capsid Protein; TMP, Tape Measure Protein. TR, Tandem Repeat.

Supplement Table 4: SNP variations and small in-del (insertion-deletion) variations found in the phage K53 reference genome in comparison to other phage genomes included in MT1-ST2.

| **Location of the variant** | **Locus**  **_tag** | **Function of the phage protein** | **Change**  **(K2 genome**  **--> variant)** | **Polymorphism Type** | **Variant Frequency (%)** | **Number of genomes with the variant** |
| --- | --- | --- | --- | --- | --- | --- |
| 6547 |  |  | A -> G | SNP (transition) | 33.3 | 1 |
| 10149 | K53_015 |  | A -> C | SNP (transversion) | 33.3 | 1 |
| 10841 | K53_017 |  | A -> C | SNP (transversion) | 33.3 | 1 |
| 12018 | K53_019 |  | M* -> A | SNP | 100.0 | 3 |
| 13815 | K53_022 | HNH homing endonuclease | A -> C | SNP (transversion) | 100.0 | 3 |
| 14335 | K53_023 |  | M* -> A | SNP | 66.7 | 2 |
| 14335 | K53_023 |  | M* -> C | SNP | 33.3 | 1 |
| 14379 | K53_023 |  | A -> C | SNP (transversion) | 33.3 | 1 |
| 15345 | K53_023 |  | TTCTACCT | Insertion | 33.3 | 1 |
| 19280 | K53_028 |  | M* -> A | SNP | 100.0 | 3 |
| 25475 | K53_035 | DNA helicase | M* -> A | SNP | 100.0 | 3 |
| 37823 | K53_052 |  | A -> G | SNP (transition) | 33.3 | 1 |
| 37825 | K53_052 |  | GG | Insertion | 33.3 | 1 |
| 37903 to 38030 | K53_053 |  | K*TACTCGACCTAAGGAAGAAGACAAGCGCAAGCGTAAACCTAAGCCGCGAGTAAAGCCAGCTAATGAGATGCCGGATGTGTACCCCCGCATACCAGGGGTGCACCAGCCTAAGKATTGTGTAGGCAAA | Deletion | 100.0 | 3 |

*Imprecise base calling by Illumina sequencing. M, Adenine /Cytosine. K, Guanine / Thymine.

Supplement Table 5: SNP variations and small in-del (insertion-deletion) variations found in the phage K6 reference genome in comparison to other phage genomes included in MT1-ST3.

| **Location of the variant** | **Locus**  **_tag** | **Function of the phage protein** | **Change**  **(K6 genome**  **--> variant)** | **Polymorphism Type** | **Variant Frequency (%)** | **Number of genomes with the variant** |
| --- | --- | --- | --- | --- | --- | --- |
| 130 | K6_001 | Terminase, small subunit | C -> T | SNP (transition) | 50.0 | 1 |
| 2265 | K6_003 | Structure | A -> G | SNP (transition) | 50.0 | 1 |
| 5581 | K6_006 | Fibritin (wac) | C -> T | SNP (transition) | 50.0 | 1 |
| 5593 | K6_006 | Fibritin (wac) | C -> T | SNP (transition) | 50.0 | 1 |
| 5641 | K6_006 | Fibritin (wac) | C -> T | SNP (transition) | 50.0 | 1 |
| 5644 | K6_006 | Fibritin (wac) | T -> C | SNP (transition) | 50.0 | 1 |
| 5650 | K6_006 | Fibritin (wac) | A -> G | SNP (transition) | 50.0 | 1 |
| 5656 | K6_006 | Fibritin (wac) | A -> G | SNP (transition) | 50.0 | 1 |
| 5676 |  |  | A -> G | SNP (transition) | 50.0 | 1 |
| 5684 |  |  | T -> C | SNP (transition) | 50.0 | 1 |
| 5686 |  |  | T -> G | SNP (transversion) | 50.0 | 1 |
| 5688 |  |  | G -> C | SNP (transversion) | 50.0 | 1 |
| 5690 |  |  | C -> A | SNP (transversion) | 50.0 | 1 |
| 5709 to 5711 |  |  | GTA-> ACC | Substitution | 50.0 | 1 |
| 5732 |  |  | T -> C | SNP (transition) | 50.0 | 1 |
| 5744 |  |  | A -> C | SNP (transversion) | 50.0 | 1 |
| 6081 | K6_008 |  | T -> G | SNP (transversion) | 50.0 | 1 |
| 7658 | K6_010 | MCP | G -> A | SNP (transition) | 50.0 | 1 |
| 7667 | K6_010 | MCP | G -> A | SNP (transition) | 50.0 | 1 |
| 7673 | K6_010 | MCP | C -> A | SNP (transversion) | 50.0 | 1 |
| 7691 | K6_010 | MCP | T -> C | SNP (transition) | 50.0 | 1 |
| 7697 | K6_010 | MCP | T -> C | SNP (transition) | 50.0 | 1 |
| 7703 | K6_010 | MCP | T -> C | SNP (transition) | 50.0 | 1 |
| 7706 | K6_010 | MCP | C -> T | SNP (transition) | 50.0 | 1 |
| 7715 | K6_010 | MCP | T -> G | SNP (transversion) | 50.0 | 1 |
| 7718 | K6_010 | MCP | T -> C | SNP (transition) | 50.0 | 1 |
| 7736 | K6_010 | MCP | T -> C | SNP (transition) | 50.0 | 1 |
| 7742 | K6_010 | MCP | A -> G | SNP (transition) | 50.0 | 1 |
| 7754 | K6_010 | MCP | G -> C | SNP (transversion) | 50.0 | 1 |
| 7766 | K6_010 | MCP | A -> C | SNP (transversion) | 50.0 | 1 |
| 7769 | K6_010 | MCP | C -> T | SNP (transition) | 50.0 | 1 |
| 10011 | K6_015 |  | A -> T | SNP (transversion) | 50.0 | 1 |
| 10050 | K6_015 |  | C -> T | SNP (transition) | 50.0 | 1 |
| 10053 | K6_015 |  | C -> T | SNP (transition) | 50.0 | 1 |
| 10062 | K6_015 |  | C -> T | SNP (transition) | 50.0 | 1 |
| 10068 | K6_015 |  | C -> T | SNP (transition) | 50.0 | 1 |
| 10071 | K6_015 |  | C -> T | SNP (transition) | 50.0 | 1 |
| 10089 | K6_015 |  | A -> G | SNP (transition) | 50.0 | 1 |
| 10095 | K6_015 |  | A -> G | SNP (transition) | 50.0 | 1 |
| 10098 | K6_015 |  | T -> G | SNP (transversion) | 50.0 | 1 |
| 10113 | K6_015 |  | A -> G | SNP (transition) | 50.0 | 1 |
| 10116 | K6_015 |  | G -> C | SNP (transversion) | 50.0 | 1 |
| 10138 | K6_015 |  | T -> C | SNP (transition) | 50.0 | 1 |
| 10146 | K6_015 |  | T -> C | SNP (transition) | 50.0 | 1 |
| 10161 | K6_015 |  | A -> T | SNP (transversion) | 50.0 | 1 |
| 10167 | K6_015 |  | A -> C | SNP (transversion) | 50.0 | 1 |
| 10172 to 10173 | K6_015 |  | CG -> TC | Substitution | 50.0 | 1 |
| 10179 | K6_015 |  | A -> G | SNP (transition) | 50.0 | 1 |
| 10185 | K6_015 |  | G -> T | SNP (transversion) | 50.0 | 1 |
| 10240 | K6_015 |  | G -> A | SNP (transition) | 50.0 | 1 |
| 10500 | K6_016 |  | C -> T | SNP (transition) | 50.0 | 1 |
| 10514 | K6_016 |  | C -> T | SNP (transition) | 50.0 | 1 |
| 10828 | K6_017 |  | C -> T | SNP (transition) | 50.0 | 1 |
| 11030 | K6_017 |  | C -> T | SNP (transition) | 50.0 | 1 |
| 11170 | K6_018 |  | A -> T | SNP (transversion) | 50.0 | 1 |
| 11232 | K6_018 |  | A -> G | SNP (transition) | 50.0 | 1 |
| 11250 | K6_018 |  | A -> G | SNP (transition) | 50.0 | 1 |
| 11477 | K6_018 |  | C -> T | SNP (transition) | 50.0 | 1 |
| 11523 | K6_018 |  | T -> C | SNP (transition) | 50.0 | 1 |
| 11529 | K6_018 |  | C -> A | SNP (transversion) | 50.0 | 1 |
| 11570 | K6_019 |  | G -> A | SNP (transition) | 50.0 | 1 |
| 11599 | K6_019 |  | T -> G | SNP (transversion) | 50.0 | 1 |
| 11604 to 11606 | K6_019 |  | ACG->GAA | Substitution | 50.0 | 1 |
| 11609 | K6_019 |  | G -> C | SNP (transversion) | 50.0 | 1 |
| 11615 | K6_019 |  | T -> G | SNP (transversion) | 50.0 | 1 |
| 11624 | K6_019 |  | C -> A | SNP (transversion) | 50.0 | 1 |
| 11626 | K6_019 |  | A -> C | SNP (transversion) | 50.0 | 1 |
| 11630 | K6_019 |  | T -> C | SNP (transition) | 50.0 | 1 |
| 11636 | K6_019 |  | G -> A | SNP (transition) | 50.0 | 1 |
| 11645 | K6_019 |  | G -> A | SNP (transition) | 50.0 | 1 |
| 11657 | K6_019 |  | T -> A | SNP (transversion) | 50.0 | 1 |
| 11666 | K6_019 |  | T -> C | SNP (transition) | 50.0 | 1 |
| 11669 | K6_019 |  | G -> C | SNP (transversion) | 50.0 | 1 |
| 11672 | K6_019 |  | C -> G | SNP (transversion) | 50.0 | 1 |
| 11678 | K6_019 |  | G -> C | SNP (transversion) | 50.0 | 1 |
| 11681 | K6_019 |  | T -> A | SNP (transversion) | 50.0 | 1 |
| 11699 | K6_019 |  | C -> T | SNP (transition) | 50.0 | 1 |
| 11716 | K6_019 |  | G -> A | SNP (transition) | 50.0 | 1 |
| 11727 | K6_019 |  | T -> G | SNP (transversion) | 50.0 | 1 |
| 11738 | K6_019 |  | A -> T | SNP (transversion) | 50.0 | 1 |
| 11741 | K6_019 |  | T -> C | SNP (transition) | 50.0 | 1 |
| 11747 | K6_019 |  | A -> T | SNP (transversion) | 50.0 | 1 |
| 11756 | K6_019 |  | T -> A | SNP (transversion) | 50.0 | 1 |
| 11762 | K6_019 |  | A -> G | SNP (transition) | 50.0 | 1 |
| 11769 to 11771 | K6_019 |  | GTC-> ATT | Substitution | 50.0 | 1 |
| 11774 | K6_019 |  | A -> G | SNP (transition) | 50.0 | 1 |
| 11777 | K6_019 |  | G -> A | SNP (transition) | 50.0 | 1 |
| 11780 | K6_019 |  | C -> T | SNP (transition) | 50.0 | 1 |
| 11784 to 11785 | K6_019 |  | AC -> GA | Substitution | 50.0 | 1 |
| 11792 | K6_019 |  | G -> A | SNP (transition) | 50.0 | 1 |
| 11819 | K6_019 |  | C -> T | SNP (transition) | 50.0 | 1 |
| 11834 | K6_019 |  | C -> G | SNP (transversion) | 50.0 | 1 |
| 11852 | K6_019 |  | G -> A | SNP (transition) | 50.0 | 1 |
| 11859 to 11861 | K6_019 |  | ATT-> GTC | Substitution | 50.0 | 1 |
| 11864 | K6_019 |  | G -> A | SNP (transition) | 50.0 | 1 |
| 11867 | K6_019 |  | T -> A | SNP (transversion) | 50.0 | 1 |
| 11873 | K6_019 |  | G -> C | SNP (transversion) | 50.0 | 1 |
| 11898 | K6_019 |  | T -> G | SNP (transversion) | 50.0 | 1 |
| 11912 | K6_019 |  | A -> G | SNP (transition) | 50.0 | 1 |
| 11915 | K6_019 |  | C -> A | SNP (transversion) | 50.0 | 1 |
| 11918 | K6_019 |  | T -> C | SNP (transition) | 50.0 | 1 |
| 11924 | K6_019 |  | T -> C | SNP (transition) | 50.0 | 1 |
| 11933 | K6_019 |  | C -> T | SNP (transition) | 50.0 | 1 |
| 11948 | K6_019 |  | C -> T | SNP (transition) | 50.0 | 1 |
| 11964 | K6_019 |  | A -> C | SNP (transversion) | 50.0 | 1 |
| 11999 | K6_019 |  | G -> C | SNP (transversion) | 50.0 | 1 |
| 12005 | K6_019 |  | G -> A | SNP (transition) | 50.0 | 1 |
| 12008 | K6_019 |  | A -> C | SNP (transversion) | 50.0 | 1 |
| 12023 | K6_019 |  | C -> T | SNP (transition) | 50.0 | 1 |
| 12050 | K6_019 |  | A -> C | SNP (transversion) | 50.0 | 1 |
| 12055 | K6_019 |  | C -> T | SNP (transition) | 50.0 | 1 |
| 12070 | K6_019 |  | A -> G | SNP (transition) | 50.0 | 1 |
| 12110 | K6_019 |  | C -> A | SNP (transversion) | 50.0 | 1 |
| 12116 | K6_019 |  | A -> G | SNP (transition) | 50.0 | 1 |
| 12119 | K6_019 |  | C -> T | SNP (transition) | 50.0 | 1 |
| 12122 | K6_019 |  | C -> T | SNP (transition) | 50.0 | 1 |
| 12125 | K6_019 |  | A -> G | SNP (transition) | 50.0 | 1 |
| 12140 | K6_019 |  | A -> G | SNP (transition) | 50.0 | 1 |
| 14238 | K6_022 | HNH homing endonuclease | G -> A | SNP (transition) | 50.0 | 1 |
| 14347 | K6_023 |  | G -> A | SNP (transition) | 50.0 | 1 |
| 17176 | K6_027 | TMP | G -> A | SNP (transition) | 50.0 | 1 |
| 17296 | K6_027 | TMP | A -> G | SNP (transition) | 50.0 | 1 |
| 18525 | K6_027 | TMP | G -> A | SNP (transition) | 50.0 | 1 |
| 18658 | K6_027 | TMP | A -> G | SNP (transition) | 50.0 | 1 |
| 21219 | K6_031 |  | T -> C | SNP (transition) | 50.0 | 1 |
| 21925 | K6_031 |  | A -> G | SNP (transition) | 50.0 | 1 |
| 22009 | K6_031 |  | G -> C | SNP (transversion) | 50.0 | 1 |
| 22012 | K6_031 |  | G -> A | SNP (transition) | 50.0 | 1 |
| 22147 | K6_031 |  | T -> C | SNP (transition) | 50.0 | 1 |
| 22174 | K6_031 |  | C -> T | SNP (transition) | 50.0 | 1 |
| 22180 | K6_031 |  | T -> C | SNP (transition) | 50.0 | 1 |
| 22239 | K6_031 |  | A -> G | SNP (transition) | 50.0 | 1 |
| 22438 | K6_031 |  | T -> C | SNP (transition) | 50.0 | 1 |
| 22484 | K6_031 |  | G -> T | SNP (transversion) | 50.0 | 1 |
| 22570 | K6_031 |  | T -> C | SNP (transition) | 50.0 | 1 |
| 22594 | K6_031 |  | T -> C | SNP (transition) | 50.0 | 1 |
| 22597 | K6_031 |  | C -> T | SNP (transition) | 50.0 | 1 |
| 22600 | K6_031 |  | A -> C | SNP (transversion) | 50.0 | 1 |
| 22615 | K6_031 |  | T -> C | SNP (transition) | 50.0 | 1 |
| 22630 | K6_031 |  | G -> A | SNP (transition) | 50.0 | 1 |
| 22633 | K6_031 |  | T -> C | SNP (transition) | 50.0 | 1 |
| 22684 | K6_031 |  | C -> T | SNP (transition) | 50.0 | 1 |
| 22759 | K6_031 |  | A -> G | SNP (transition) | 50.0 | 1 |
| 22768 | K6_031 |  | C -> T | SNP (transition) | 50.0 | 1 |
| 22828 | K6_031 |  | C -> T | SNP (transition) | 50.0 | 1 |
| 22837 | K6_031 |  | A -> G | SNP (transition) | 50.0 | 1 |
| 22840 | K6_031 |  | G -> T | SNP (transversion) | 50.0 | 1 |
| 22865 | K6_031 |  | A -> G | SNP (transition) | 50.0 | 1 |
| 22879 | K6_031 |  | C -> T | SNP (transition) | 50.0 | 1 |
| 22904 to 22906 | K6_032 | Tailspike | TCT -> CCC | Substitution | 50.0 | 1 |
| 22925 | K6_032 | Tailspike | T -> A | SNP (transversion) | 50.0 | 1 |
| 22936 | K6_032 | Tailspike | C -> T | SNP (transition) | 50.0 | 1 |
| 22946 | K6_032 | Tailspike | G -> A | SNP (transition) | 50.0 | 1 |
| 22951 | K6_032 | Tailspike | T -> C | SNP (transition) | 50.0 | 1 |
| 22981 | K6_032 | Tailspike | G -> T | SNP (transversion) | 50.0 | 1 |
| 22990 | K6_032 | Tailspike | C -> T | SNP (transition) | 50.0 | 1 |
| 22996 | K6_032 | Tailspike | G -> A | SNP (transition) | 50.0 | 1 |
| 23008 | K6_032 | Tailspike | T -> C | SNP (transition) | 50.0 | 1 |
| 23014 | K6_032 | Tailspike | C -> T | SNP (transition) | 50.0 | 1 |
| 23017 | K6_032 | Tailspike | A -> G | SNP (transition) | 50.0 | 1 |
| 23040 | K6_032 | Tailspike | A -> C | SNP (transversion) | 50.0 | 1 |
| 23062 | K6_032 | Tailspike | G -> A | SNP (transition) | 50.0 | 1 |
| 23065 to 23068 | K6_032 | Tailspike | CTTG->GCTT | Substitution | 50.0 | 1 |
| 23074 | K6_032 | Tailspike | G -> A | SNP (transition) | 50.0 | 1 |
| 23077 | K6_032 | Tailspike | T -> C | SNP (transition) | 50.0 | 1 |
| 23085 | K6_032 | Tailspike | A -> G | SNP (transition) | 50.0 | 1 |
| 23104 | K6_032 | Tailspike | G -> A | SNP (transition) | 50.0 | 1 |
| 23110 | K6_032 | Tailspike | C -> T | SNP (transition) | 50.0 | 1 |
| 23113 | K6_032 | Tailspike | C -> T | SNP (transition) | 50.0 | 1 |
| 23116 | K6_032 | Tailspike | C -> T | SNP (transition) | 50.0 | 1 |
| 23119 | K6_032 | Tailspike | A -> G | SNP (transition) | 50.0 | 1 |
| 23125 | K6_032 | Tailspike | C -> G | SNP (transversion) | 50.0 | 1 |
| 23128 | K6_032 | Tailspike | G -> T | SNP (transversion) | 50.0 | 1 |
| 23131 | K6_032 | Tailspike | C -> T | SNP (transition) | 50.0 | 1 |
| 23134 | K6_032 | Tailspike | G -> C | SNP (transversion) | 50.0 | 1 |
| 23137 | K6_032 | Tailspike | A -> T | SNP (transversion) | 50.0 | 1 |
| 23139 to 23141 | K6_032 | Tailspike | CTG-> TAA | Substitution | 50.0 | 1 |
| 23149 | K6_032 | Tailspike | G -> A | SNP (transition) | 50.0 | 1 |
| 23158 | K6_032 | Tailspike | G -> T | SNP (transversion) | 50.0 | 1 |
| 23170 | K6_032 | Tailspike | T -> G | SNP (transversion) | 50.0 | 1 |
| 23172 to 23173 | K6_032 | Tailspike | TT -> AA | Substitution | 50.0 | 1 |
| 23176 | K6_032 | Tailspike | G -> A | SNP (transition) | 50.0 | 1 |
| 23179 | K6_032 | Tailspike | C -> T | SNP (transition) | 50.0 | 1 |
| 23182 | K6_032 | Tailspike | C -> T | SNP (transition) | 50.0 | 1 |
| 23197 | K6_032 | Tailspike | T -> C | SNP (transition) | 50.0 | 1 |
| 23202 | K6_032 | Tailspike | T -> C | SNP (transition) | 50.0 | 1 |
| 23209 | K6_032 | Tailspike | C -> T | SNP (transition) | 50.0 | 1 |
| 23212 | K6_032 | Tailspike | G -> C | SNP (transversion) | 50.0 | 1 |
| 23215 | K6_032 | Tailspike | A -> C | SNP (transversion) | 50.0 | 1 |
| 23218 | K6_032 | Tailspike | C -> G | SNP (transversion) | 50.0 | 1 |
| 23221 | K6_032 | Tailspike | T -> G | SNP (transversion) | 50.0 | 1 |
| 23257 | K6_032 | Tailspike | G -> C | SNP (transversion) | 50.0 | 1 |
| 23274 | K6_032 | Tailspike | C -> T | SNP (transition) | 50.0 | 1 |
| 23283 | K6_032 | Tailspike | T -> A | SNP (transversion) | 50.0 | 1 |
| 23345 | K6_032 | Tailspike | T -> C | SNP (transition) | 50.0 | 1 |
| 23350 | K6_032 | Tailspike | G -> C | SNP (transversion) | 50.0 | 1 |
| 23353 | K6_032 | Tailspike | T -> C | SNP (transition) | 50.0 | 1 |
| 23359 | K6_032 | Tailspike | C -> T | SNP (transition) | 50.0 | 1 |
| 23362 | K6_032 | Tailspike | T -> G | SNP (transversion) | 50.0 | 1 |
| 23368 | K6_032 | Tailspike | G -> A | SNP (transition) | 50.0 | 1 |
| 23374 | K6_032 | Tailspike | A -> G | SNP (transition) | 50.0 | 1 |
| 23377 | K6_032 | Tailspike | T -> G | SNP (transversion) | 50.0 | 1 |
| 23383 | K6_032 | Tailspike | T -> C | SNP (transition) | 50.0 | 1 |
| 23389 | K6_032 | Tailspike | C -> T | SNP (transition) | 50.0 | 1 |
| 23392 | K6_032 | Tailspike | C -> T | SNP (transition) | 50.0 | 1 |
| 23395 | K6_032 | Tailspike | C -> T | SNP (transition) | 50.0 | 1 |
| 23404 | K6_032 | Tailspike | C -> T | SNP (transition) | 50.0 | 1 |
| 23410 | K6_032 | Tailspike | T -> C | SNP (transition) | 50.0 | 1 |
| 23413 | K6_032 | Tailspike | C -> T | SNP (transition) | 50.0 | 1 |
| 23418 to 23419 | K6_032 | Tailspike | AT -> CC | Substitution | 50.0 | 1 |
| 23422 | K6_032 | Tailspike | C -> T | SNP (transition) | 50.0 | 1 |
| 23424 | K6_032 | Tailspike | G -> C | SNP (transversion) | 50.0 | 1 |
| 23429 to 23431 | K6_032 | Tailspike | AAC->GGT | Substitution | 50.0 | 1 |
| 23437 | K6_032 | Tailspike | C -> T | SNP (transition) | 50.0 | 1 |
| 23449 | K6_032 | Tailspike | C -> A | SNP (transversion) | 50.0 | 1 |
| 23452 | K6_032 | Tailspike | T -> G | SNP (transversion) | 50.0 | 1 |
| 23455 to 23457 | K6_032 | Tailspike | GAC->AGT | Substitution | 50.0 | 1 |
| 23459 to 23461 | K6_032 | Tailspike | CTG-> TTA | Substitution | 50.0 | 1 |
| 23467 | K6_032 | Tailspike | A -> T | SNP (transversion) | 50.0 | 1 |
| 23479 | K6_032 | Tailspike | G -> T | SNP (transversion) | 50.0 | 1 |
| 23499 to 23500 | K6_032 | Tailspike | TG -> GC | Substitution | 50.0 | 1 |
| 23503 | K6_032 | Tailspike | A -> T | SNP (transversion) | 50.0 | 1 |
| 23506 | K6_032 | Tailspike | C -> T | SNP (transition) | 50.0 | 1 |
| 23508 to 23511 | K6_032 | Tailspike | GGGA->TCAC | Substitution | 50.0 | 1 |
| 23518 | K6_032 | Tailspike | C -> T | SNP (transition) | 50.0 | 1 |
| 23521 to 23523 | K6_032 | Tailspike | CGA->TAG | Substitution | 50.0 | 1 |
| 23534 to 23546 | K6_032 | Tailspike | TTGAACCAGCCGC -> GTAGTTGGTGCTT | Substitution | 50.0 | 1 |
| 23552 | K6_032 | Tailspike | C -> G | SNP (transversion) | 50.0 | 1 |
| 23556 to 23560 | K6_032 | Tailspike | CAAAA->GTGTC | Substitution | 50.0 | 1 |
| 23571 to 23584 | K6_032 | Tailspike | ATACTGTATATAGA -> GGGTGATTAAACCG | Substitution | 50.0 | 1 |
| 23586 to 23589 | K6_032 | Tailspike | TCGA->GGAC | Substitution | 50.0 | 1 |
| 23594 to 23598 | K6_032 | Tailspike | AACGG->GATAA | Substitution | 50.0 | 1 |
| 23600 to 23602 | K6_032 | Tailspike | AAC->CAG | Substitution | 50.0 | 1 |
| 23606 to 23608 | K6_032 | Tailspike | GTG->ATA | Substitution | 50.0 | 1 |
| 23612 to 23614 | K6_032 | Tailspike | AAT->GAC | Substitution | 50.0 | 1 |
| 23617 to 23627 | K6_032 | Tailspike | GTCAACGGTGC -> CGCGGCAATCG | Substitution | 50.0 | 1 |
| 23632 to 23638 | K6_032 | Tailspike | GTCGGTA->CACCCTT | Substitution | 50.0 | 1 |
| 23640 to 23641 | K6_032 | Tailspike | CT -> AA | Substitution | 50.0 | 1 |
| 23644 to 23647 | K6_032 | Tailspike | AAGG->GTCT | Substitution | 50.0 | 1 |
| 23649 to 23656 | K6_032 | Tailspike | TGGACAAA -> AAACAGAT | Substitution | 50.0 | 1 |
| 23659 | K6_032 | Tailspike | G -> A | SNP (transition) | 50.0 | 1 |
| 23662 to 23663 | K6_032 | Tailspike | TA -> CC | Substitution | 50.0 | 1 |
| 23668 | K6_032 | Tailspike | T -> G | SNP (transversion) | 50.0 | 1 |
| 23670 to 23674 | K6_032 | Tailspike | ACATT->CGGTA | Substitution | 50.0 | 1 |
| 23677 | K6_032 | Tailspike | T -> C | SNP (transition) | 50.0 | 1 |
| 23682 to 23683 | K6_032 | Tailspike | TG -> AC | Substitution | 50.0 | 1 |
| 23685 to 23686 | K6_032 | Tailspike | AT -> CC | Substitution | 50.0 | 1 |
| 23688 to 23689 | K6_032 | Tailspike | TC -> AG | Substitution | 50.0 | 1 |
| 23691 to 23699 | K6_032 | Tailspike | GGGACAGCT -> TCCCTGGTA | Substitution | 50.0 | 1 |
| 23702 to 23706 | K6_032 | Tailspike | CCCGA -> GAATCCCTTCTCCCTCC | Insertion | 50.0 | 1 |
| 23708 to 23713 | K6_032 | Tailspike | AACATA -> GAAGCT | Substitution | 50.0 | 1 |
| 23717 to 23719 | K6_032 | Tailspike | AAT->GGG | Substitution | 50.0 | 1 |
| 23723 to 23734 | K6_032 | Tailspike | ACAGCCGGTGCA -> GGCATATCCTCT | Substitution | 50.0 | 1 |
| 23737 | K6_032 | Tailspike | A -> C | SNP (transversion) | 50.0 | 1 |
| 23740 to 23748 | K6_032 | Tailspike | CCGCGTTAT -> GGAAATCCG | Substitution | 50.0 | 1 |
| 23750 to 23760 | K6_032 | Tailspike | AGCGGGTCAAA -> GAATGTACGGG | Substitution | 50.0 | 1 |
| 23762 to 23767 | K6_032 | Tailspike | ATAATC -> GTTGAG | Substitution | 50.0 | 1 |
| 23770 to 23782 | K6_032 | Tailspike | AAATTCCCCGGAA -> CCACCGGGCAAGC | Substitution | 50.0 | 1 |
| 23784 to 23787 | K6_032 | Tailspike | CGAC->GTCT | Substitution | 50.0 | 1 |
| 23789 to 23791 | K6_032 | Tailspike | TTC-> ATG | Substitution | 50.0 | 1 |
| 23793 to 23797 | K6_032 | Tailspike | GTGGC -> CGTGT | Substitution | 50.0 | 1 |
| 23799 to 23803 | K6_032 | Tailspike | ATTTA -> TCCTG | Substitution | 50.0 | 1 |
| 23807 to 23811 | K6_032 | Tailspike | ACCCT -> CGTGG | Substitution | 50.0 | 1 |
| 23815 to 23816 | K6_032 | Tailspike | TA -> CC | Substitution | 50.0 | 1 |
| 23819 to 23826 | K6_032 | Tailspike | CGTATATT -> TTCTGTAA | Substitution | 50.0 | 1 |
| 23828 to 23832 | K6_032 | Tailspike | GTTAA -> ATGGT | Substitution | 50.0 | 1 |
| 23834 | K6_032 | Tailspike | A -> G | SNP (transition) | 50.0 | 1 |
| 23837 to 23842 | K6_032 | Tailspike | CCACGA -> GCTGAC | Substitution | 50.0 | 1 |
| 23845 to 23848 | K6_032 | Tailspike | TTTT -> CCCG | Substitution | 50.0 | 1 |
| 23850 to 23851 | K6_032 | Tailspike | TT -> GC | Substitution | 50.0 | 1 |
| 23853 to 23857 | K6_032 | Tailspike | CATGG ->GCGGT | Substitution | 50.0 | 1 |
| 23859 to 23863 | K6_032 | Tailspike | AATCA-> TGCAC | Substitution | 50.0 | 1 |
| 23866 to 23869 | K6_032 | Tailspike | AATT-> CGTA | Substitution | 50.0 | 1 |
| 23871 to 23874 | K6_032 | Tailspike | CTTT-> TCAC | Substitution | 50.0 | 1 |
| 23876 to 23882 | K6_032 | Tailspike | GAAAACC -> TTCGAAA | Substitution | 50.0 | 1 |
| 23885 to 23887 | K6_032 | Tailspike | CAT-> TTA | Substitution | 50.0 | 1 |
| 23889 to 23893 | K6_032 | Tailspike | CATCC-> GCGGA | Substitution | 50.0 | 1 |
| 23895 to 23896 | K6_032 | Tailspike | CA -> AT | Substitution | 50.0 | 1 |
| 23903 to23905 | K6_032 | Tailspike | TAT-> AAA | Substitution | 50.0 | 1 |
| 23913 to 23914 | K6_032 | Tailspike | GG -> AT | Substitution | 50.0 | 1 |
| 23917 to 23920 | K6_032 | Tailspike | TGTA-> CATT | Substitution | 50.0 | 1 |
| 23923 | K6_032 | Tailspike | A -> T | SNP (transversion) | 50.0 | 1 |
| 23926 to 23929 | K6_032 | Tailspike | GGAG->TAGA | Substitution | 50.0 | 1 |
| 23931 to 23932 | K6_032 | Tailspike | TA -> CT | Substitution | 50.0 | 1 |
| 23934 to 23935 | K6_032 | Tailspike | AG -> GC | Substitution | 50.0 | 1 |
| 23941 | K6_032 | Tailspike | T -> G | SNP (transversion) | 50.0 | 1 |
| 23946 to 23949 | K6_032 | Tailspike | GGTC->TAAG | Substitution | 50.0 | 1 |
| 23951 to 23953 | K6_032 | Tailspike | GCC-> AGT | Substitution | 50.0 | 1 |
| 23955 | K6_032 | Tailspike | T -> C | SNP (transition) | 50.0 | 1 |
| 23957 to 23958 | K6_032 | Tailspike | TT -> CA | Substitution | 50.0 | 1 |
| 23963 to 23965 | K6_032 | Tailspike | ATT -> TTA | Substitution | 50.0 | 1 |
| 23968 | K6_032 | Tailspike | C -> T | SNP (transition) | 50.0 | 1 |
| 23971 to 23974 | K6_032 | Tailspike | CGAC->TAAT | Substitution | 50.0 | 1 |
| 23981 to 23983 | K6_032 | Tailspike | GAT-> TTC | Substitution | 50.0 | 1 |
| 23986 | K6_032 | Tailspike | C -> A | SNP (transversion) | 50.0 | 1 |
| 23988 to 23989 | K6_032 | Tailspike | AT -> GC | Substitution | 50.0 | 1 |
| 24001 | K6_032 | Tailspike | G -> T | SNP (transversion) | 50.0 | 1 |
| 24003 to 24004 | K6_032 | Tailspike | GA -> TT | Substitution | 50.0 | 1 |
| 24006 to 24008 | K6_032 | Tailspike | ACC-> GGT | Substitution | 50.0 | 1 |
| 24012 to 24013 | K6_032 | Tailspike | TA -> CC | Substitution | 50.0 | 1 |
| 24016 | K6_032 | Tailspike | T -> A | SNP (transversion) | 50.0 | 1 |
| 24019 | K6_032 | Tailspike | T -> C | SNP (transition) | 50.0 | 1 |
| 24024 | K6_032 | Tailspike | T -> C | SNP (transition) | 50.0 | 1 |
| 24037 | K6_032 | Tailspike | G -> T | SNP (transversion) | 50.0 | 1 |
| 24040 | K6_032 | Tailspike | A -> T | SNP (transversion) | 50.0 | 1 |
| 24043 | K6_032 | Tailspike | A -> G | SNP (transition) | 50.0 | 1 |
| 24046 | K6_032 | Tailspike | T -> G | SNP (transversion) | 50.0 | 1 |
| 24048 to 24049 | K6_032 | Tailspike | AC -> GG | Substitution | 50.0 | 1 |
| 24053 to 24057 | K6_032 | Tailspike | AACGA-> GGTAC | Substitution | 50.0 | 1 |
| 24059 | K6_032 | Tailspike | A -> G | SNP (transition) | 50.0 | 1 |
| 24064 to 24067 | K6_032 | Tailspike | CGGC-> TTCT | Substitution | 50.0 | 1 |
| 24069 to 24074 | K6_032 | Tailspike | GGTCCG -> CAACGT | Substitution | 50.0 | 1 |
| 24079 | K6_032 | Tailspike | G -> T | SNP (transversion) | 50.0 | 1 |
| 24086 to 24087 | K6_032 | Tailspike | CG -> AA | Substitution | 50.0 | 1 |
| 24092 to 24093 | K6_032 | Tailspike | GT -> CA | Substitution | 50.0 | 1 |
| 24098 to 24099 | K6_032 | Tailspike | GA -> CG | Substitution | 50.0 | 1 |
| 24101 | K6_032 | Tailspike | A -> G | SNP (transition) | 50.0 | 1 |
| 24104 to 24107 | K6_032 | Tailspike | ATTA-> TCCG | Substitution | 50.0 | 1 |
| 24110 to 24124 | K6_032 | Tailspike | ACCATACAGTGCTAT -> GTTTTGTACCCAGTA | Substitution | 50.0 | 1 |
| 24126 to 24127 | K6_032 | Tailspike | AC -> GG | Substitution | 50.0 | 1 |
| 24130 | K6_032 | Tailspike | C -> T | SNP (transition) | 50.0 | 1 |
| 24133 to 24136 | K6_032 | Tailspike | GATA-> ATTC | Substitution | 50.0 | 1 |
| 24140 to 24145 | K6_032 | Tailspike | GTCAAC -> TTGGGA | Substitution | 50.0 | 1 |
| 24148 | K6_032 | Tailspike | A -> G | SNP (transition) | 50.0 | 1 |
| 24156 to 24165 | K6_032 | Tailspike | GTTCCCCGGT -> ACATGAATCC | Substitution | 50.0 | 1 |
| 24169 to 24171 | K6_032 | Tailspike | GCG->AGA | Substitution | 50.0 | 1 |
| 24174 | K6_032 | Tailspike | -T | Deletion | 50.0 | 1 |
| 24176 to 24186 | K6_032 | Tailspike | GATGATTACCC -> CCGCCCGGGGG | Substitution | 50.0 | 1 |
| 24188 | K6_032 | Tailspike | C -> T | SNP (transition) | 50.0 | 1 |
| 24194 to 24195 | K6_032 | Tailspike | -CA | Deletion | 50.0 | 1 |
| 24200 to 24205 | K6_032 | Tailspike | CCGTGG -> TCTCAA | Substitution | 50.0 | 1 |
| 24207 to 24208 | K6_032 | Tailspike | TT -> AC | Substitution | 50.0 | 1 |
| 24210 | K6_032 | Tailspike | A -> C | SNP (transversion) | 50.0 | 1 |
| 24212 to 24214 | K6_032 | Tailspike | TTG-> GTA | Substitution | 50.0 | 1 |
| 24216 to 24217 | K6_032 | Tailspike | CG -> AT | Substitution | 50.0 | 1 |
| 24219 to 24223 | K6_032 | Tailspike | CTAAA -> TGCTC | Substitution | 50.0 | 1 |
| 24225 to 24227 | K6_032 | Tailspike | ACA-> CAT | Substitution | 50.0 | 1 |
| 24231 to 24232 | K6_032 | Tailspike | TT -> AC | Substitution | 50.0 | 1 |
| 24234 to 24238 | K6_032 | Tailspike | GCAAC -> ATTTG | Substitution | 50.0 | 1 |
| 24241 to 24243 | K6_032 | Tailspike | AAT-> TGA | Substitution | 50.0 | 1 |
| 24246 to 24252 | K6_032 | Tailspike | CGAAGGA -> ATCTATT | Substitution | 50.0 | 1 |
| 24254 to 24256 | K6_032 | Tailspike | TGC -> GTT | Substitution | 50.0 | 1 |
| 24258 to 24259 | K6_032 | Tailspike | TG -> GA | Substitution | 50.0 | 1 |
| 24263 to 24268 | K6_032 | Tailspike | ATTGGT -> TCGCTG | Substitution | 50.0 | 1 |
| 24270 to 24280 | K6_032 | Tailspike | CCTGGTGGGAT -> GGGTAGGTTTC | Substitution | 50.0 | 1 |
| 24284 to 24285 | K6_032 | Tailspike | CA -> AT | Substitution | 50.0 | 1 |
| 24287 to 24292 | K6_032 | Tailspike | AATAAT -> GACGGG | Substitution | 50.0 | 1 |
| 24294 to 24302 | K6_032 | Tailspike | TCATAGATA -> AAGGTCTGT | Substitution | 50.0 | 1 |
| 24305 | K6_032 | Tailspike | AT -> GTC | Insertion | 50.0 | 1 |
| 24308 to 24310 | K6_032 | Tailspike | GTG-> CTA | Substitution | 50.0 | 1 |
| 24316 to 24318 | K6_032 | Tailspike | TGA-> ACC | Substitution | 50.0 | 1 |
| 24320 to 24325 | K6_032 | Tailspike | GCCCAC -> TCGAGG | Substitution | 50.0 | 1 |
| 24327 to 24328 | K6_032 | Tailspike | AA -> TT | Substitution | 50.0 | 1 |
| 24330 | K6_032 | Tailspike | A -> C | SNP (transversion) | 50.0 | 1 |
| 24332 to 24333 | K6_032 | Tailspike | GG -> CT | Substitution | 50.0 | 1 |
| 24335 | K6_032 | Tailspike | A -> G | SNP (transition) | 50.0 | 1 |
| 24338 to 24340 | K6_032 | Tailspike | TTT -> CTG | Substitution | 50.0 | 1 |
| 24342 to 24345 | K6_032 | Tailspike | ATAG->GGCTT | Insertion | 50.0 | 1 |
| 24347 to 24348 | K6_032 | Tailspike | GG -> TA | Substitution | 50.0 | 1 |
| 24350 | K6_032 | Tailspike | A -> T | SNP (transversion) | 50.0 | 1 |
| 24353 to 24354 | K6_032 | Tailspike | AA -> TG | Substitution | 50.0 | 1 |
| 24356 to 24361 | K6_032 | Tailspike | AATGAT -> CCACGA | Substitution | 50.0 | 1 |
| 24363 | K6_032 | Tailspike | T -> A | SNP (transversion) | 50.0 | 1 |
| 24366 | K6_032 | Tailspike | C -> G | SNP (transversion) | 50.0 | 1 |
| 24369 to 24373 | K6_032 | Tailspike | ACGTA->TTC | Deletion | 50.0 | 1 |
| 24376 to 24378 | K6_032 | Tailspike | GGT-> TAA | Substitution | 50.0 | 1 |
| 24382 | K6_032 | Tailspike | A -> C | SNP (transversion) | 50.0 | 1 |
| 24384 to 24388 | K6_032 | Tailspike | GTGCG->CAATA | Substitution | 50.0 | 1 |
| 24390 | K6_032 | Tailspike | A -> T | SNP (transversion) | 50.0 | 1 |
| 24392 to 24400 | K6_032 | Tailspike | AAGGATTTA -> GACACCAAT | Substitution | 50.0 | 1 |
| 24406 to 24412 | K6_032 | Tailspike | CCTGAAT -> AAACTTC | Substitution | 50.0 | 1 |
| 24414 to 24418 | K6_032 | Tailspike | AGCTT -> CCGCG | Substitution | 50.0 | 1 |
| 24420 | K6_032 | Tailspike | C -> A | SNP (transversion) | 50.0 | 1 |
| 24422 to 24426 | K6_032 | Tailspike | TGCGA-> CAGAT | Substitution | 50.0 | 1 |
| 24428 to 24429 | K6_032 | Tailspike | GG -> TA | Substitution | 50.0 | 1 |
| 24432 to 24448 | K6_032 | Tailspike | GTAGCCGATTGCGGGGC -> TCTCAGGGGCATGCCGT | Substitution | 50.0 | 1 |
| 24452 to 24457 | K6_032 | Tailspike | TTGATT -> AACGGC | Substitution | 50.0 | 1 |
| 24459 | K6_032 | Tailspike | A -> T | SNP (transversion) | 50.0 | 1 |
| 24461 to 24463 | K6_032 | Tailspike | GCG->CGC | Substitution | 50.0 | 1 |
| 24465 to 24468 | K6_032 | Tailspike | ATAC-> TAGT | Substitution | 50.0 | 1 |
| 24470 to 24478 | K6_032 | Tailspike | ACAGAAGGA -> GGCATCCGT | Substitution | 50.0 | 1 |
| 24480 to 24482 | K6_032 | Tailspike | ATG->CAA | Substitution | 50.0 | 1 |
| 24485 to 24500 | K6_032 | Tailspike | GTGTATGCACCACAAT -> ACCGAACAGGGCATGA | Substitution | 50.0 | 1 |
| 24503 to 24504 | K6_032 | Tailspike | GA -> AT | Substitution | 50.0 | 1 |
| 24506 to 24512 | K6_032 | Tailspike | ATATCCG -> GACGCAC | Substitution | 50.0 | 1 |
| 24515 to 24516 | K6_032 | Tailspike | GT -> AA | Substitution | 50.0 | 1 |
| 24518 | K6_032 | Tailspike | G -> T | SNP (transversion) | 50.0 | 1 |
| 24521 to 24523 | K6_032 | Tailspike | TGC-> ACT | Substitution | 50.0 | 1 |
| 24525 to 24531 | K6_032 | Tailspike | CCGGTTC -> TAAGCGG | Substitution | 50.0 | 1 |
| 24533 to 24535 | K6_032 | Tailspike | GGT-> ATA | Substitution | 50.0 | 1 |
| 24539 to 24559 | K6_032 | Tailspike | AAAAAGATACTTTGTACCTAT -> GGCTTCGTGGACCCATCAAGG | Substitution | 50.0 | 1 |
| 24563 to 24565 | K6_032 | Tailspike | GGT->AAC | Substitution | 50.0 | 1 |
| 24567 to 24588 | K6_032 | Tailspike | AT -> TA | Substitution | 50.0 | 1 |
| 24570 to 24588 | K6_032 | Tailspike | TACAGGGTGGCAACATTAA -> CCAATTTGATGGAGGAAGG | Substitution | 50.0 | 1 |
| 24590 to 24604 | K6_032 | Tailspike | GTCCAGCATAACGAA ->CTTGGTAACTCTCGC | Substitution | 50.0 | 1 |
| 24606 to 24610 | K6_032 | Tailspike | ACCAG-> TAAAC | Substitution | 50.0 | 1 |
| 24612 to 24615 | K6_032 | Tailspike | TGAC-> GTTT | Substitution | 50.0 | 1 |
| 24617 to 24618 | K6_032 | Tailspike | CT -> AA | Substitution | 50.0 | 1 |
| 24620 to 24621 | K6_032 | Tailspike | GC -> AA | Substitution | 50.0 | 1 |
| 24624 to 24631 | K6_032 | Tailspike | TGCGTCCT ->ATTCCGCG | Substitution | 50.0 | 1 |
| 24634 to 24645 | K6_032 | Tailspike | GATGGGCGGTAC -> TCTACAGTTGCG | Substitution | 50.0 | 1 |
| 24649 to 24654 | K6_032 | Tailspike | AAATCC -> CCACAA | Substitution | 50.0 | 1 |
| 24656 to 24657 | K6_032 | Tailspike | TC -> CT | Substitution | 50.0 | 1 |
| 24659 to 24660 | K6_032 | Tailspike | CT -> AC | Substitution | 50.0 | 1 |
| 24662 to 24663 | K6_032 | Tailspike | GT -> AA | Substitution | 50.0 | 1 |
| 24666 to 24670 | K6_032 | Tailspike | TGACC -> CCCTT | Substitution | 50.0 | 1 |
| 24672 to 24681 | K6_032 | Tailspike | CAGATTGCCA -> ATAGCGGAGC | Substitution | 50.0 | 1 |
| 24683 to 24687 | K6_032 | Tailspike | GTTGC -> TTGTA | Substitution | 50.0 | 1 |
| 24689 to 24691 | K6_032 | Tailspike | CTA-> TCC | Substitution | 50.0 | 1 |
| 24693 to 24700 | K6_032 | Tailspike | CTGGGGGT->ACATAAAC | Substitution | 50.0 | 1 |
| 24702 to 24703 | K6_032 | Tailspike | AA -> GC | Substitution | 50.0 | 1 |
| 24705 to 24711 | K6_032 | Tailspike | CTAGTAT -> GGCCAGG | Substitution | 50.0 | 1 |
| 24713 to 24714 | K6_032 | Tailspike | GT -> TC | Substitution | 50.0 | 1 |
| 24716 to 24722 | K6_032 | Tailspike | AAGCTTT -> GGTTCGG | Substitution | 50.0 | 1 |
| 24725 to 24726 | K6_032 | Tailspike | GC -> TG | Substitution | 50.0 | 1 |
| 24729 to 24735 | K6_032 | Tailspike | TACAGGA -> CCGAAAT | Substitution | 50.0 | 1 |
| 24737 to 24739 | K6_032 | Tailspike | GGT-> ACC | Substitution | 50.0 | 1 |
| 24741 to 24745 | K6_032 | Tailspike | TACGA -> CTATT | Substitution | 50.0 | 1 |
| 24747 | K6_032 | Tailspike | T -> C | SNP (transition) | 50.0 | 1 |
| 24751 | K6_032 | Tailspike | -T | Deletion | 50.0 | 1 |
| 24757 to 24760 | K6_032 | Tailspike | CCAA->TGCC | Substitution | 50.0 | 1 |
| 24762 to 24765 | K6_032 | Tailspike | TTAA->GATG | Substitution | 50.0 | 1 |
| 24769 to 24776 | K6_032 | Tailspike | CTTAGGTT -> TGTCA | Deletion | 50.0 | 1 |
| 24782 | K6_032 | Tailspike | C -> A | SNP (transversion) | 50.0 | 1 |
| 24786 to 24793 | K6_032 | Tailspike | TGAGCATA -> AATAGGGG | Substitution | 50.0 | 1 |
| 24795 | K6_032 | Tailspike | C -> G | SNP (transversion) | 50.0 | 1 |
| 24797 | K6_032 | Tailspike | G -> T | SNP (transversion) | 50.0 | 1 |
| 24802 to 24805 | K6_032 | Tailspike | CCCA-> GTGCTG | Insertion | 50.0 | 1 |
| 24807 | K6_032 | Tailspike | C -> T | SNP (transition) | 50.0 | 1 |
| 24809 to 24810 | K6_032 | Tailspike | CA -> AG | Substitution | 50.0 | 1 |
| 24813 to 24814 | K6_032 | Tailspike | TA -> AC | Substitution | 50.0 | 1 |
| 24816 | K6_032 | Tailspike | C -> T | SNP (transition) | 50.0 | 1 |
| 24818 | K6_032 | Tailspike | G -> T | SNP (transversion) | 50.0 | 1 |
| 24821 to 24828 | K6_032 | Tailspike | AGTGCATT -> CGGTGTCC | Substitution | 50.0 | 1 |
| 24830 to 24839 | K6_032 | Tailspike | GAACATAACT -> TCCTACCAGA | Substitution | 50.0 | 1 |
| 24842 to 24859 | K6_032 | Tailspike | TCCATAGGCTTTTTCTTT ->ACGCTGTCAGGGATAACG | Substitution | 50.0 | 1 |
| 24862 to 24867 | K6_032 | Tailspike | AAGTGA -> TCTATAT | Insertion | 50.0 | 1 |
| 24869 to 24871 | K6_032 | Tailspike | GGG->ACT | Substitution | 50.0 | 1 |
| 24876 to 24878 | K6_032 | Tailspike | TGC->CTG | Substitution | 50.0 | 1 |
| 24881 to 24886 | K6_032 | Tailspike | ATCCTC -> GGGTGATAGCCTTAAAGCGTTG | Insertion | 50.0 | 1 |
| 24888 to 24889 | K6_032 | Tailspike | CC -> TT | Substitution | 50.0 | 1 |
| 24893 to 24898 | K6_032 | Tailspike | AAACCA -> CGGGCC | Substitution | 50.0 | 1 |
| 24901 | K6_032 | Tailspike | C -> T | SNP (transition) | 50.0 | 1 |
| 24904 to 24907 | K6_032 | Tailspike | GGCT->AAGC | Substitution | 50.0 | 1 |
| 24910 to 24912 | K6_032 | Tailspike | CGT-> TAC | Substitution | 50.0 | 1 |
| 24915 to 24916 | K6_032 | Tailspike | CC -> GA | Substitution | 50.0 | 1 |
| 24918 to 24919 | K6_032 | Tailspike | AT -> TA | Substitution | 50.0 | 1 |
| 24922 to 24928 | K6_032 | Tailspike | CCTATCC -> TTTGGCA | Substitution | 50.0 | 1 |
| 24930 | K6_032 | Tailspike | G -> A | SNP (transition) | 50.0 | 1 |
| 24948 |  |  | T -> A | SNP (transversion) | 50.0 | 1 |
| 24950 |  |  | -T | Deletion | 50.0 | 1 |
| 24981 | K6_033 |  | G -> A | SNP (transition) | 50.0 | 1 |
| 24983 to 24986 | K6_033 |  | AGTG->CGCA | Substitution | 50.0 | 1 |
| 24991 | K6_033 |  | A -> G | SNP (transition) | 50.0 | 1 |
| 25003 | K6_033 |  | C -> T | SNP (transition) | 50.0 | 1 |
| 25028 | K6_033 |  | A -> G | SNP (transition) | 50.0 | 1 |
| 25034 | K6_033 |  | C -> T | SNP (transition) | 50.0 | 1 |
| 25040 | K6_033 |  | A -> C | SNP (transversion) | 50.0 | 1 |
| 25056 to 25059 | K6_034 |  | CAGT-> TTGC | Substitution | 50.0 | 1 |
| 25061 | K6_034 |  | C -> T | SNP (transition) | 50.0 | 1 |
| 25065 | K6_034 |  | G -> C | SNP (transversion) | 50.0 | 1 |
| 25075 | K6_034 |  | T -> C | SNP (transition) | 50.0 | 1 |
| 25086 | K6_034 |  | T -> G | SNP (transversion) | 50.0 | 1 |
| 25100 | K6_034 |  | A -> G | SNP (transition) | 50.0 | 1 |
| 25102 | K6_034 |  | G -> A | SNP (transition) | 50.0 | 1 |
| 25108 | K6_034 |  | C -> G | SNP (transversion) | 50.0 | 1 |
| 25123 | K6_034 |  | T -> C | SNP (transition) | 50.0 | 1 |
| 25132 | K6_034 |  | T -> C | SNP (transition) | 50.0 | 1 |
| 25147 | K6_034 |  | G -> C | SNP (transversion) | 50.0 | 1 |
| 25154 | K6_034 |  | G -> T | SNP (transversion) | 50.0 | 1 |
| 25162 to 25164 | K6_034 |  | CGC -> TTT | Substitution | 50.0 | 1 |
| 25166 to 25167 | K6_034 |  | AA -> TG | Substitution | 50.0 | 1 |
| 25171 | K6_034 |  | CG -> 496 bp* | Insertion | 50.0 | 1 |
| 25177 to 25178 | K6_034 |  | CC -> AT | Substitution | 50.0 | 1 |
| 25180 to 25181 | K6_034 |  | CA -> AG | Substitution | 50.0 | 1 |
| 25185 to 25188 | K6_034 |  | CTTC-> TAAG | Substitution | 50.0 | 1 |
| 25191 | K6_034 |  | T -> G | SNP (transversion) | 50.0 | 1 |
| 25193 to 25197 | K6_034 |  | GCTTT->AACTC | Substitution | 50.0 | 1 |
| 25203 to 25208 | K6_034 |  | TGCTAC -> AATCCA | Substitution | 50.0 | 1 |
| 25221 | K6_035 | Helicase | C -> T | SNP (transition) | 50.0 | 1 |
| 25251 | K6_035 | Helicase | T -> C | SNP (transition) | 50.0 | 1 |
| 25254 | K6_035 | Helicase | G -> T | SNP (transversion) | 50.0 | 1 |
| 25257 | K6_035 | Helicase | A -> C | SNP (transversion) | 50.0 | 1 |
| 25263 | K6_035 | Helicase | T -> C | SNP (transition) | 50.0 | 1 |
| 25278 | K6_035 | Helicase | C -> T | SNP (transition) | 50.0 | 1 |
| 25281 | K6_035 | Helicase | A -> G | SNP (transition) | 50.0 | 1 |
| 25287 | K6_035 | Helicase | G -> A | SNP (transition) | 50.0 | 1 |
| 25314 | K6_035 | Helicase | T -> G | SNP (transversion) | 50.0 | 1 |
| 25323 | K6_035 | Helicase | C -> T | SNP (transition) | 50.0 | 1 |
| 25329 | K6_035 | Helicase | C -> T | SNP (transition) | 50.0 | 1 |
| 25332 | K6_035 | Helicase | T -> C | SNP (transition) | 50.0 | 1 |
| 25335 | K6_035 | Helicase | A -> G | SNP (transition) | 50.0 | 1 |
| 25338 | K6_035 | Helicase | C -> T | SNP (transition) | 50.0 | 1 |
| 25344 | K6_035 | Helicase | G -> T | SNP (transversion) | 50.0 | 1 |
| 25347 | K6_035 | Helicase | A -> C | SNP (transversion) | 50.0 | 1 |
| 25365 | K6_035 | Helicase | A -> G | SNP (transition) | 50.0 | 1 |
| 25368 | K6_035 | Helicase | A -> G | SNP (transition) | 50.0 | 1 |
| 25401 | K6_035 | Helicase | C -> A | SNP (transversion) | 50.0 | 1 |
| 34045 | K6_044 | Helicase, repA | C -> T | SNP (transition) | 50.0 | 1 |
| 38178 | K6_051 |  | A -> G | SNP (transition) | 50.0 | 1 |
| 38507 | K6_053 |  | A -> G | SNP (transition) | 50.0 | 1 |
| 38545 | K6_053 |  | C -> T | SNP (transition) | 50.0 | 1 |
| 38608 | K6_053 |  | G -> A | SNP (transition) | 50.0 | 1 |
| 38847 | K6_053 |  | C -> T | SNP (transition) | 50.0 | 1 |
| 42884 | K6_067 |  | C -> T | SNP (transition) | 50.0 | 1 |

MCP, Major Capsid Protein; TMP, Tape Measure Protein.

*TGCCGGAATCGGGGCTATGATGTTCATCTGGTAAATTTCCTCGCAAGATACGTTATTTCTTCTTCACAAAGCTCATCAAGGTCGTTAGATACAAATCCTCTGGTGAATGTTTTGCTGAACTTTAAGCCCGCTTTATCATTGTCACCGGCGACACCCATTCATACGGCAAAAGTGACATTTGCTGCCGTAAGTCAGCTGGTATGTTCGACCCCAATGCGCTAACTGCATTAAACCCGCAATTCATCAAAGCAACAGCTTTAAATATGCTTTCGGTAACAAATACCACCCCGCTCCTCGGCAAATACTCAAGTCCCCATAAACACGGCCTTGTTGTTCGGGTAAAGTACCGCGCGTCTTTAGGGTTTTTACAGTTCTTTTCAGCATATGCTTGTAATGCTGGTATCCTCGTAGCCTACCGTCAAAGCCCCACAAGTAGAATGTAGCAACGCCAGGGGCAAGACTACCTGGAGACGGTCTGCGTCAAACCCACGCGACA

Supplement Table 6: SNP variations and small in-del (insertion-deletion) variations found in the phage K22 reference genome in comparison to other phage genomes included MT1-ST4.

| **Location of the variant** | **Change**  **(K9 genome --> variant)** | **Polymorphism Type** | **Variant Frequency (%)** | **Number of genomes with the variant** |
| --- | --- | --- | --- | --- |
| 37486 | (G)12 -> (G)13 | Insertion (tandem repeat) | 100.0% | 1 |

Supplement Table 7: SNP variations and small in-del (insertion-deletion) variations found in the phage K9 reference genome in comparison to other phage genomes included in MT1-ST4.

| **Location of the variant** | **Locus**  **_tag** | **Function of the phage protein** | **Change**  **(K9 genome**  **--> variant)** | **Polymorphism Type** | **Variant Frequency (%)** | **Number of genomes with the variant** |
| --- | --- | --- | --- | --- | --- | --- |
| 509 | K9_001 | Terminase, small subunit | T -> C | SNP (transition) | 100.0 | 1 |
| 7367 | K9_009 | Major capsid protein | G -> T | SNP (transversion) | 100.0 | 1 |
| 17376 | K9_026 | Tail tail tape measure | T -> G | SNP (transversion) | 100.0 | 1 |
| 22323 | K9_030 |  | A -> C | SNP (transversion) | 100.0 | 1 |
| 42156 | K9_063 |  | C -> T | SNP (transition) | 100.0 | 1 |

Supplement Table 8: SNP variations and small in-del (insertion-deletion) variations found in K16 reference genome in comparison to other phage genomes included in MT2-ST1.

| **Location of the variant** | **Locus**  **_tag** | **Function of the phage protein** | **Change**  **(K16 genome**  **--> variant)** | **Polymorphism Type** | **Variant Frequency (%)** | **Number of genomes with the variant** |
| --- | --- | --- | --- | --- | --- | --- |
| 4366 |  |  | T -> G | SNP (transversion) | 25.0 | 1 |
| 5310 to 5340 |  |  | _TCATCACCACTATAATAATAACTATAAGAAA | Deletion | 25.0 | 1 |
| 7725 | K16_011 |  | G -> T | SNP (transversion) | 100.0 | 4 |
| 7778 | K16_011 |  | T -> G | SNP (transversion) | 25.0 | 1 |
| 7794 | K16_011 |  | T -> G | SNP (transversion) | 25.0 | 1 |
| 23960 to 23961 | K16_048 |  | GA -> AG | Substitution | 25.0 | 1 |
| 24061 | K16_048 |  | T -> C | SNP (transition) | 25.0 | 1 |
| 24119 | K16_048 |  | A -> G | SNP (transition) | 25.0 | 1 |
| 24165 | K16_048 |  | T -> A | SNP (transversion) | 25.0 | 1 |
| 24167 | K16_048 |  | C -> T | SNP (transition) | 25.0 | 1 |
| 24185 | K16_048 |  | G -> A | SNP (transition) | 25.0 | 1 |
| 24194 | K16_048 |  | A -> T | SNP (transversion) | 25.0 | 1 |
| 24197 to 24199 | K16_048 |  | CAC -> TAT | Substitution | 25.0 | 1 |
| 24211 | K16_048 |  | C -> A | SNP (transversion) | 25.0 | 1 |
| 24227 | K16_048 |  | T -> A | SNP (transversion) | 25.0 | 1 |
| 24244 | K16_049 |  | C -> T | SNP (transition) | 25.0 | 1 |
| 24270 | K16_049 |  | C -> T | SNP (transition) | 25.0 | 1 |
| 24333 to 24334 | K16_049 |  | GC -> AT | Substitution | 25.0 | 1 |
| 24340 | K16_049 |  | A -> G | SNP (transition) | 25.0 | 1 |
| 24379 | K16_049 |  | C -> A | SNP (transversion) | 25.0 | 1 |
| 24400 | K16_049 |  | A -> C | SNP (transversion) | 25.0 | 1 |
| 24408 to 24409 | K16_049 |  | CA -> TG | Substitution | 25.0 | 1 |
| 24456 to 24457 | K16_049 |  | TA -> CG | Substitution | 25.0 | 1 |
| 24460 | K16_049 |  | C -> T | SNP (transition) | 25.0 | 1 |
| 24466 | K16_049 |  | A -> C | SNP (transversion) | 25.0 | 1 |
| 24514 | K16_049 |  | A -> G | SNP (transition) | 25.0 | 1 |
| 24514 | K16_050 |  | A -> G | SNP (transition) | 25.0 | 1 |
| 24677 | K16_050 |  | G -> A | SNP (transition) | 25.0 | 1 |
| 34807 |  |  | G -> T | SNP (transversion) | 25.0 | 1 |
| 35764 | K16_074 |  | C -> T | SNP (transition) | 25.0 | 1 |
| 35779 to 35781 | K16_074 |  | (GAT)3 -> (GAT)2 | Deletion (TR*) | 25.0 | 1 |
| 35791 | K16_074 |  | C -> T | SNP (transition) | 25.0 | 1 |
| 35932 | K16_074 |  | T -> A | SNP (transversion) | 25.0 | 1 |
| 35959 | K16_074 |  | G -> A | SNP (transition) | 25.0 | 1 |
| 35974 | K16_074 |  | A -> T | SNP (transversion) | 25.0 | 1 |
| 36088 | K16_074 |  | A -> G | SNP (transition) | 25.0 | 1 |
| 36094 | K16_074 |  | T -> C | SNP (transition) | 25.0 | 1 |
| 36106 | K16_074 |  | G -> A | SNP (transition) | 25.0 | 1 |
| 36112 | K16_074 |  | G -> T | SNP (transversion) | 25.0 | 1 |
| 36127 | K16_074 |  | C -> T | SNP (transition) | 25.0 | 1 |
| 36190 | K16_074 |  | T -> C | SNP (transition) | 25.0 | 1 |
| 36322 | K16_074 |  | A -> G | SNP (transition) | 25.0 | 1 |
| 36502 | K16_074 |  | T -> C | SNP (transition) | 25.0 | 1 |
| 36505 | K16_074 |  | A -> G | SNP (transition) | 25.0 | 1 |
| 36520 | K16_074 |  | G -> A | SNP (transition) | 25.0 | 1 |
| 36527 | K16_074 |  | T -> G | SNP (transversion) | 25.0 | 1 |
| 36532 | K16_074 |  | A -> G | SNP (transition) | 25.0 | 1 |
| 36537 | K16_074 |  | A -> G | SNP (transition) | 25.0 | 1 |
| 36572 | K16_074 |  | G -> T | SNP (transversion) | 25.0 | 1 |
| 36638 |  |  | G -> A | SNP (transition) | 25.0 | 1 |
| 36642 |  |  | A -> G | SNP (transition) | 25.0 | 1 |
| 36694 | K16_075 |  | A -> T | SNP (transversion) | 25.0 | 1 |
| 36713 | K16_075 |  | A -> G | SNP (transition) | 25.0 | 1 |
| 36721 to 36722 | K16_075 |  | CT -> TC | Substitution | 25.0 | 1 |
| 36745 | K16_075 |  | A -> G | SNP (transition) | 25.0 | 1 |
| 36811 | K16_075 |  | G -> A | SNP (transition) | 25.0 | 1 |
| 36818 | K16_075 |  | G -> A | SNP (transition) | 25.0 | 1 |
| 36857 | K16_075 |  | C -> T | SNP (transition) | 25.0 | 1 |
| 36878 | K16_075 |  | G -> A | SNP (transition) | 25.0 | 1 |
| 36883 | K16_075 |  | T -> G | SNP (transversion) | 25.0 | 1 |
| 36901 | K16_075 |  | G -> A | SNP (transition) | 25.0 | 1 |
| 36985 | K16_075 |  | G -> A | SNP (transition) | 25.0 | 1 |
| 37034 | K16_075 |  | A -> G | SNP (transition) | 25.0 | 1 |
| 37052 to 37054 | K16_075 |  | AGC -> GGT | Substitution | 25.0 | 1 |
| 37074 |  |  | G -> A | SNP (transition) | 25.0 | 1 |
| 37254 to 37257 |  |  | ACAA -> CACC | Substitution | 25.0 | 1 |
| 37261 to 37262 |  |  | TA -> AG | Substitution | 25.0 | 1 |
| 37264 |  |  | A -> G | SNP (transition) | 25.0 | 1 |
| 37275 to 37277 |  |  | -GAA | Deletion | 25.0 | 1 |
| 37283 |  |  | C -> A | SNP (transversion) | 25.0 | 1 |
| 37285 to 37286 |  |  | AT -> CA | Substitution | 25.0 | 1 |
| 37296 |  |  | -A | Deletion | 25.0 | 1 |
| 37298 |  |  | T -> C | SNP (transition) | 25.0 | 1 |
| 37305 to 37306 |  |  | AC -> G | Deletion | 25.0 | 1 |
| 37308 |  |  | T -> A | SNP (transversion) | 25.0 | 1 |
| 37355 |  |  | T -> G | SNP (transversion) | 25.0 | 1 |
| 37490 | K16_076 | SPFH domain | T -> C | SNP (transition) | 25.0 | 1 |
| 37493 | K16_076 | SPFH domain | G -> A | SNP (transition) | 25.0 | 1 |
| 43383 |  |  | A -> G | SNP (transition) | 100.0 | 4 |
| 100214 | K16_144 | Portal connector | T -> C | SNP (transition) | 25.0 | 1 |
| 100232 | K16_144 | Portal connector | G -> A | SNP (transition) | 25.0 | 1 |
| 100298 | K16_144 | Portal connector | T -> C | SNP (transition) | 25.0 | 1 |

*TR, tandem repeat

Supplement Table 9: The SNP variations and small in-del (insertion-deletion) variations found in phage K36 reference genome in comparison to other phage genomes included in MT2-ST2.

| **Location of the variant** | **Locus**  **_tag** | **Function of the phage protein** | **Change**  **(K36 genome**  **--> variant)** | **Polymorphism Type** | **Variant Frequency (%)** | **Number of genomes with the variant** |
| --- | --- | --- | --- | --- | --- | --- |
| 87199 | K36_118 | Tail tape-measure protein | C -> A | SNP (transversion) | 100.0 | 1 |
| 105108 | K36_136 |  | +GAAAGATATGTCAAACAAAAGAAA  GAGCAGACGTGGGGAAGTGTACGA  TAGCTGGATTAATAGTTTTGGATTTG  GGACTATCCTATTCTTTATTTTCGTA  GGTTTTTGGTTAGCAGCTATGGCGGGG | Insertion | 100.0 | 1 |

Supplement Table 10: SNP variations and small in-del (insertion-deletion) variations found in phage K37 reference genome in comparison to other phage genomes included in MT2-ST6.

| **Location of the variant** | **Locus**  **_tag** | **Function of the phage protein** | **Change**  **(K37 genome**  **--> variant)** | **Polymorphism Type** | **Variant Frequency (%)** | **Number of genomes with the variant** |
| --- | --- | --- | --- | --- | --- | --- |
| 1113 | K37_002 |  | +TATATACCTTTGCGCTCGCTAGCTGATCATCGAGGCAAGCGGGGCAAGGCGCGACAACTGCAAAGGCTAGACCGGATTTTACGGCGTCAATAATGATTGTTTCTTGTT  TCACCGTATCAACATAAAC | Insertion | 100.0 | 1 |
| 8973 |  |  | (T)13 -> (T)12 | Deletion (tandem repeat) | 100.0 | 1 |
| 72259 | K37_119 | DNA helicase | A -> G | SNP (transition) | 100.0 | 1 |

Supplement Table 11: SNP variations and small in-del (insertion-deletion) variations found in phage K38 reference genome in comparison to other phage genomes included in MT2-ST8.

| **Location of the variant** | **Locus**  **_tag** | **Function of the phage protein** | **Change**  **(K38 genome**  **--> variant)** | **Polymorphism Type** | **Variant Frequency (%)** | **Number of genomes with the variant** |
| --- | --- | --- | --- | --- | --- | --- |
| 8007 | K38_013 |  | C -> T | SNP (transition) | 100.0 | 1 |
| 16928 | K38_037 | Serine/threonine phosphatase | C -> A | SNP (transversion) | 100.0 | 1 |
| 81922 to 81924 | K38_127 |  | TAG -> ACA | Substitution | 50.0 | 0.5 |
| 81928 to 81930 | K38_127 | Long tail fiber | ATC -> CTA | Substitution | 50.0 | 0.5 |
| 81936 | K38_127 | Long tail fiber | C -> T | SNP (transition) | 100.0 | 1 |

Supplement Table 12: SNP variations and small in-del (insertion-deletion) variations found in phage K30 reference genome in comparison to other phage genomes included in MT3-ST1.

| **Location of the variant** | **Locus**  **_tag** | **Function of the phage protein** | **Change**  **(K30 genome**  **--> variant)** | **Polymorphism Type** | **Variant Frequency (%)** | **Number of genomes with the variant** |
| --- | --- | --- | --- | --- | --- | --- |
| 1106 to 1137 | K30_005 |  | TGTTTTCACTAGAAAGTGGTATTGGCGGGCGT | Deletion | 66.7  -> 100.0 | 2 |
| 12550 |  |  | (A)3 -> (A)2 | Deletion (TR) | 33.3 | 1 |
| 14579 |  |  | (T)6 -> (T)5 | Deletion (TR) | 66.7 | 2 |
| 15914 | K30_027 | Exonuclease | T -> G | SNP (transversion) | 66.7 | 2 |
| 24107 | K30_039 | Tail tubular protein | C -> A | SNP (transversion) | 66.7 | 2 |
| 28960 | K30_042 | Lysozyme domain | C -> A | SNP (transversion) | 66.7 | 2 |
| 38455 | K30_048 |  | A -> C | SNP (transversion) | 66.7 | 2 |
| 38484 | K30_048 |  | C -> T | SNP (transition) | 33.3 | 1 |
| 38706 | K30_049 |  | G -> A | SNP (transition) | 75.0 | 3 |
| 38728 | K30_049 |  | A -> G | SNP (transition) | 75.0 | 3 |
| 41445 | K30_055 | Tailspike | A -> C | SNP (transversion) | 100.0 | 3 |
| 41460 | K30_055 | Tailspike | +GAG | Insertion | 33.3 | 1 |
| 41464 to 41466 | K30_055 | Tailspike | -AGA | Deletion | 66.7 | 2 |
| 41570 | K30_055 | Tailspike | G -> T | SNP (transversion) | 33.3 | 1 |
| 42248 to 42251 |  |  | GTTA -> TAAC | Substitution | 33.3 | 1 |

*TR, tandem repeat.

Supplement Table 13: tRNA genes in MT2 genomes.

| **Phage isolate** | **tRNA type** | **Minimum (bp)** | **Maximum (bp)** | **Length (bp)** |
| --- | --- | --- | --- | --- |
| K15 | tRNA-Ser-GCT | 34778 | 34863 | 86 |
|  | tRNA-Tyr-GTA | 33147 | 33231 | 85 |
|  | tRNA-Asn-GTT | 32228 | 32307 | 80 |
|  | tRNA-Lys-TTT | 30149 | 30223 | 75 |
|  | tRNA-Met-CAT | 30868 | 30942 | 75 |
|  | tRNA-Met-CAT | 34694 | 34768 | 75 |
|  | tRNA-Leu-TAG | 29620 | 29693 | 74 |
|  | tRNA-Glu-TTC | 33064 | 33137 | 74 |
|  | tRNA-Leu-TAA | 34113 | 34186 | 74 |
|  | tRNA-Ile-GAT | 27002 | 27075 | 74 |
|  | tRNA-Gln-TTG | 28248 | 28320 | 73 |
|  | tRNA-Gln-CTG | 28330 | 28402 | 73 |
|  | tRNA-Pro-TGG | 30952 | 31024 | 73 |
|  | tRNA-Cys-GCA | 32463 | 32535 | 73 |
|  | tRNA-Met-CAT | 26831 | 26903 | 73 |
|  | tRNA-Arg-TCT | 38406 | 38477 | 72 |
|  | tRNA-Val-TAC | 30066 | 30136 | 71 |
| K16 | tRNA-Ser-GCT | 34751 | 34836 | 86 |
|  | tRNA-Thr-TGT | 27782 | 27865 | 84 |
|  | tRNA-Leu-TAG | 29655 | 29736 | 82 |
|  | tRNA-Asn-GTT | 32196 | 32275 | 80 |
|  | tRNA-Lys-TTT | 30568 | 30643 | 76 |
|  | tRNA-Met-CAT | 34667 | 34741 | 75 |
|  | tRNA-Met-CAT | 30836 | 30910 | 75 |
|  | tRNA-Leu-TAA | 34086 | 34159 | 74 |
|  | tRNA-Ile-GAT | 26955 | 27028 | 74 |
|  | tRNA-Glu-TTC | 33032 | 33105 | 74 |
|  | tRNA-Met-CAT | 26783 | 26855 | 73 |
|  | tRNA-Gln-TTG | 28386 | 28458 | 73 |
|  | tRNA-Gln-CTG | 28468 | 28540 | 73 |
|  | tRNA-Pro-TGG | 30920 | 30992 | 73 |
|  | tRNA-Cys-GCA | 32431 | 32503 | 73 |
|  | tRNA-Arg-TCT | 38379 | 38450 | 72 |
|  | tRNA-Ala-TGC | 30208 | 30279 | 72 |
| K18 | tRNA-Ser-GCT | 37420 | 37505 | 86 |
|  | tRNA-Leu-TAG | 32693 | 32772 | 80 |
|  | tRNA-Lys-TTT | 33640 | 33715 | 76 |
|  | tRNA-Met-CAT | 33908 | 33982 | 75 |
|  | tRNA-Glu-TTC | 36461 | 36534 | 74 |
|  | tRNA-Ile-GAT | 30075 | 30148 | 74 |
|  | tRNA-Met-CAT | 29903 | 29975 | 73 |
|  | tRNA-Gln-TTG | 31321 | 31393 | 73 |
|  | tRNA-Gln-CTG | 31403 | 31475 | 73 |
|  | tRNA-Pro-TGG | 33992 | 34064 | 73 |
|  | tRNA-Cys-GCA | 35860 | 35932 | 73 |
|  | tRNA-Met-CAT | 37339 | 37410 | 72 |
|  | tRNA-Arg-TCT | 41047 | 41118 | 72 |
|  | tRNA-Ala-TGC | 33280 | 33351 | 72 |
|  | tRNA-Asp-GTC | 34894 | 34965 | 72 |
| K20 | tRNA-Leu-TAG | 28865 | 28946 | 82 |
|  | tRNA-Tyr-GTA | 31340 | 31420 | 81 |
|  | tRNA-Arg-TCT | 36470 | 36543 | 74 |
|  | tRNA-Ile-GAT | 26443 | 26516 | 74 |
|  | tRNA-Met-CAT | 26271 | 26343 | 73 |
|  | tRNA-Gln-TTG | 27493 | 27565 | 73 |
|  | tRNA-Gln-CTG | 27575 | 27647 | 73 |
|  | tRNA-Cys-GCA | 30905 | 30977 | 73 |
|  | tRNA-Met-CAT | 32754 | 32825 | 72 |
| K36 | tRNA-Ser-GCT | 30715 | 30800 | 86 |
|  | tRNA-Leu-TAG | 27824 | 27905 | 82 |
|  | tRNA-Tyr-GTA | 29913 | 29993 | 81 |
|  | tRNA-Cys-GCA | 29476 | 29549 | 74 |
|  | tRNA-Arg-TCT | 34333 | 34406 | 74 |
|  | tRNA-Ile-GAT | 26139 | 26211 | 73 |
|  | tRNA-Gln-TTG | 26681 | 26753 | 73 |
|  | tRNA-Gln-CTG | 26763 | 26835 | 73 |
|  | tRNA-Met-CAT | 26018 | 26090 | 73 |
|  | tRNA-Gly-TCC | 26599 | 26670 | 72 |
|  | tRNA-Asp-GTC | 28898 | 28969 | 72 |
|  | tRNA-Met-CAT | 30634 | 30705 | 72 |
| K37 | tRNA-Ser-GCT | 35220 | 35305 | 86 |
|  | tRNA-Asn-GTT | 32960 | 33039 | 80 |
|  | tRNA-Lys-TTT | 31333 | 31408 | 76 |
|  | tRNA-Met-CAT | 31601 | 31675 | 75 |
|  | tRNA-Met-CAT | 35136 | 35210 | 75 |
|  | tRNA-Ile-GAT | 27786 | 27859 | 74 |
|  | tRNA-Glu-TTC | 33796 | 33869 | 74 |
|  | tRNA-Leu-TAA | 34845 | 34918 | 74 |
|  | tRNA-Met-CAT | 27614 | 27686 | 73 |
|  | tRNA-Gln-TTG | 28837 | 28909 | 73 |
|  | tRNA-Gln-CTG | 28919 | 28991 | 73 |
|  | tRNA-Pro-TGG | 31685 | 31757 | 73 |
|  | tRNA-Cys-GCA | 33195 | 33267 | 73 |
|  | tRNA-Arg-TCT | 38845 | 38916 | 72 |
|  | tRNA-Ala-TGC | 30070 | 30140 | 71 |
|  | tRNA-Val-TAC | 30791 | 30861 | 71 |
| K38 | tRNA-Ser-GCT | 33334 | 33419 | 86 |
|  | tRNA-Thr-TGT | 27728 | 27811 | 84 |
|  | tRNA-Leu-TAG | 29700 | 29781 | 82 |
|  | tRNA-Tyr-GTA | 32293 | 32373 | 81 |
|  | tRNA-Arg-TCT | 36969 | 37042 | 74 |
|  | tRNA-Ile-GAT | 27096 | 27169 | 74 |
|  | tRNA-Met-CAT | 26924 | 26996 | 73 |
|  | tRNA-Gln-TTG | 28332 | 28404 | 73 |
|  | tRNA-Cys-GCA | 31478 | 31550 | 73 |
|  | tRNA-Met-CAT | 33253 | 33324 | 72 |
| K44 | tRNA-Pro-CGG | 37232 | 37318 | 87 |
|  | tRNA-Ser-GCT | 32507 | 32592 | 86 |
|  | tRNA-Thr-TGT | 26315 | 26398 | 84 |
|  | tRNA-Leu-TAG | 28291 | 28372 | 82 |
|  | tRNA-Tyr-GTA | 31647 | 31724 | 78 |
|  | tRNA-Pro-TGG | 28744 | 28818 | 75 |
|  | tRNA-Met-CAT | 25511 | 25583 | 73 |
|  | tRNA-Ile-GAT | 25684 | 25756 | 73 |
|  | tRNA-Gln-TTG | 26919 | 26991 | 73 |
|  | tRNA-Gln-CTG | 27001 | 27073 | 73 |
|  | tRNA-Cys-GCA | 30422 | 30494 | 73 |
|  | tRNA-Arg-TCT | 36134 | 36205 | 72 |
|  | tRNA-Glu-TTC | 31023 | 31094 | 72 |
|  | tRNA-Met-CAT | 32426 | 32497 | 72 |

Supplement Table 14: Genome comparison with NCBI BLASTn top hit genomes

| **Major type** | **Reference genome from the Kenya phage isolates** | | **Top hit genome from NCBI after BLASTn** | | | **NCBI blastn (local alignment) of 2 sequences** | | **Progressive Mauve (global alignment) of 2 sequences** | | **Phage taxonomy of the NCBI BLASTn top hit genome** | |
| --- | --- | --- | --- | --- | --- | --- | --- | --- | --- | --- | --- |
|  | Accession no. | Genome length (bp) | Phage name | Accession no. | Genome length (bp) | Query cover % | Nucleotide identity % | Mean un-gapped length of 2 sequences (bp) | Pairwise identity % | Genus | Species |
| MT1 | K47 (OQ291026) | 42,735 | Salmonella phage wast | NC_73174 | 40748 | 92 | 93 | 41728 | 76 | Jerseyvirus | - |
| MT2 | K37 (OQ291034) | 119,011 | Bacteriophage T5-like poul124 | MF431735 | 120629 | 95 | 94 | 110193 | 73 | Tequintavirus | Tequintavirus chee24 |
| MT3 | K30 (OQ291025) | 44,304 | Salmonella phage SP6 | LC730323 | 43595 | 92 | 90 | 43928 | 81 | Zindervirus | Zindervirus SP6 |
| MT4 | K19 (OQ291023) | 44,834 | Escherichia phage SECphi18 | NC_073071 | 44798 | 94 | 92 | 44766 | 84 | Dhillonvirus | - |

Supplement Table 15: Host range of all 59 phage isolates on Salmonella enterica strains from Kenya

| Phage isolate | **Enteritidis** | | | | | | | **Heidelberg** | | | | | | **Kentucky** | | | Phage sub type | Major type |
| --- | --- | --- | --- | --- | --- | --- | --- | --- | --- | --- | --- | --- | --- | --- | --- | --- | --- | --- |
|  | **Sal 16** | **Sal 177** | **Sal 568** | **Sal 569** | **Sal 572** | **Sal 73** | **Sal 312** | **Sal 157** | **Sal 192** | **Sal 194** | **Sal 187** | **Sal188** | **Sal 571** | **Sal 172** | **Sal 181** | **Sal 182** |  |  |
| K2 | 1.E+10 | 1.E+10 | 1.E+10 | 1.E+10 | 1.E+10 | 1.E+10 | 1.E+07 | 0.E+00 | 0.E+00 | 0.E+00 | 1.E+05 | 1.E+05 | 0.E+00 | 0.E+00 | 0.E+00 | 0.E+00 | 1-1 | MT1 |
| K3 | 1.E+10 | 1.E+10 | 1.E+10 | 1.E+10 | 1.E+10 | 1.E+10 | 1.E+07 | 0.E+00 | 0.E+00 | 0.E+00 | 1.E+05 | 1.E+05 | 0.E+00 | 0.E+00 | 0.E+00 | 0.E+00 |  |  |
| K4 | 1.E+10 | 1.E+10 | 1.E+10 | 1.E+10 | 1.E+10 | 1.E+10 | 1.E+07 | 0.E+00 | 0.E+00 | 0.E+00 | 1.E+05 | 1.E+05 | 0.E+00 | 0.E+00 | 0.E+00 | 0.E+00 |  |  |
| K5 | 1.E+10 | 1.E+10 | 1.E+10 | 1.E+10 | 1.E+10 | 1.E+10 | 1.E+07 | 0.E+00 | 0.E+00 | 0.E+00 | 1.E+05 | 1.E+05 | 0.E+00 | 0.E+00 | 0.E+00 | 0.E+00 |  |  |
| K23 | 1.E+10 | 1.E+10 | 1.E+10 | 1.E+10 | 1.E+10 | 1.E+10 | 1.E+06 | 0.E+00 | 0.E+00 | 0.E+00 | 1.E+04 | 1.E+05 | 0.E+00 | 1.E+00 | 1.E+00 | 1.E+00 |  |  |
| K24 | 1.E+10 | 1.E+10 | 1.E+10 | 1.E+10 | 1.E+10 | 1.E+10 | 1.E+07 | 0.E+00 | 0.E+00 | 0.E+00 | 1.E+05 | 1.E+06 | 0.E+00 | 0.E+00 | 0.E+00 | 0.E+00 |  |  |
| K25 | 1.E+10 | 1.E+10 | 1.E+10 | 1.E+10 | 1.E+10 | 1.E+10 | 1.E+07 | 0.E+00 | 0.E+00 | 0.E+00 | 1.E+05 | 1.E+05 | 0.E+00 | 0.E+00 | 0.E+00 | 0.E+00 |  |  |
| K27 | 1.E+10 | 1.E+10 | 1.E+10 | 1.E+10 | 1.E+10 | 1.E+10 | 1.E+07 | 0.E+00 | 0.E+00 | 0.E+00 | 1.E+05 | 1.E+05 | 0.E+00 | 0.E+00 | 0.E+00 | 0.E+00 |  |  |
| K28 | 1.E+10 | 1.E+10 | 1.E+10 | 1.E+10 | 1.E+10 | 1.E+10 | 1.E+06 | 0.E+00 | 0.E+00 | 0.E+00 | 1.E+05 | 1.E+05 | 0.E+00 | 0.E+00 | 0.E+00 | 0.E+00 |  |  |
| K48 | 1.E+10 | 1.E+10 | 1.E+10 | 1.E+10 | 1.E+10 | 1.E+10 | 1.E+07 | 0.E+00 | 0.E+00 | 0.E+00 | 1.E+05 | 1.E+05 | 0.E+00 | 0.E+00 | 0.E+00 | 0.E+00 |  |  |
| K49 | 1.E+10 | 1.E+10 | 1.E+10 | 1.E+10 | 1.E+10 | 1.E+10 | 1.E+07 | 0.E+00 | 0.E+00 | 0.E+00 | 1.E+05 | 1.E+05 | 0.E+00 | 0.E+00 | 0.E+00 | 0.E+00 |  |  |
| K50 | 1.E+10 | 1.E+10 | 1.E+10 | 1.E+10 | 1.E+10 | 1.E+10 | 1.E+07 | 0.E+00 | 0.E+00 | 0.E+00 | 1.E+05 | 1.E+05 | 0.E+00 | 0.E+00 | 0.E+00 | 0.E+00 |  |  |
| K51 | 1.E+10 | 1.E+10 | 1.E+10 | 1.E+10 | 1.E+10 | 1.E+10 | 1.E+07 | 0.E+00 | 0.E+00 | 0.E+00 | 1.E+05 | 1.E+05 | 0.E+00 | 0.E+00 | 0.E+00 | 0.E+00 |  |  |
| K55 | 1.E+10 | 1.E+10 | 1.E+10 | 1.E+10 | 1.E+10 | 1.E+10 | 1.E+07 | 0.E+00 | 0.E+00 | 0.E+00 | 1.E+05 | 1.E+05 | 0.E+00 | 0.E+00 | 0.E+00 | 0.E+00 |  |  |
| K60 | 1.E+10 | 1.E+10 | 1.E+10 | 1.E+10 | 1.E+10 | 1.E+10 | 1.E+07 | 0.E+00 | 0.E+00 | 0.E+00 | 1.E+05 | 1.E+05 | 0.E+00 | 0.E+00 | 0.E+00 | 0.E+00 |  |  |
| K57 | 1.E+10 | 1.E+10 | 1.E+10 | 1.E+10 | 1.E+10 | 1.E+10 | 1.E+07 | 0.E+00 | 0.E+00 | 0.E+00 | 1.E+05 | 1.E+05 | 0.E+00 | 0.E+00 | 0.E+00 | 0.E+00 |  |  |
| K59 | 1.E+10 | 1.E+10 | 1.E+10 | 1.E+10 | 1.E+10 | 1.E+10 | 1.E+07 | 0.E+00 | 0.E+00 | 0.E+00 | 1.E+05 | 1.E+05 | 0.E+00 | 0.E+00 | 0.E+00 | 0.E+00 |  |  |
| K62 | 1.E+10 | 1.E+10 | 1.E+10 | 1.E+10 | 1.E+10 | 1.E+10 | 1.E+07 | 0.E+00 | 0.E+00 | 0.E+00 | 1.E+05 | 1.E+05 | 0.E+00 | 0.E+00 | 0.E+00 | 0.E+00 |  |  |
| K63 | 1.E+10 | 1.E+10 | 1.E+10 | 1.E+10 | 1.E+10 | 1.E+10 | 1.E+06 | 0.E+00 | 0.E+00 | 0.E+00 | 1.E+05 | 1.E+05 | 0.E+00 | 0.E+00 | 0.E+00 | 0.E+00 |  |  |
| K13 | 1.E+10 | 1.E+10 | 1.E+10 | 1.E+10 | 1.E+10 | 1.E+10 | 1.E+07 | 0.E+00 | 0.E+00 | 0.E+00 | 1.E+05 | 1.E+05 | 0.E+00 | 0.E+00 | 0.E+00 | 0.E+00 |  |  |
| K52 | 1.E+10 | 1.E+10 | 1.E+10 | 1.E+10 | 1.E+10 | 1.E+10 | 1.E+07 | 0.E+00 | 0.E+00 | 0.E+00 | 1.E+05 | 1.E+05 | 0.E+00 | 0.E+00 | 0.E+00 | 0.E+00 |  |  |
| K64 | 1.E+10 | 1.E+10 | 1.E+10 | 1.E+10 | 1.E+10 | 1.E+10 | 1.E+07 | 0.E+00 | 0.E+00 | 0.E+00 | 1.E+05 | 1.E+05 | 0.E+00 | 0.E+00 | 0.E+00 | 0.E+00 |  |  |
| K66 | 1.E+10 | 1.E+10 | 1.E+09 | 1.E+09 | 1.E+10 | 1.E+10 | 1.E+06 | 0.E+00 | 0.E+00 | 0.E+00 | 1.E+05 | 1.E+00 | 0.E+00 | 0.E+00 | 0.E+00 | 0.E+00 |  |  |
| K67 | 1.E+10 | 1.E+10 | 1.E+10 | 1.E+10 | 1.E+10 | 1.E+10 | 1.E+06 | 0.E+00 | 0.E+00 | 0.E+00 | 1.E+05 | 1.E+05 | 0.E+00 | 0.E+00 | 0.E+00 | 0.E+00 |  |  |
| K68 | 1.E+10 | 1.E+10 | 1.E+10 | 1.E+10 | 1.E+10 | 1.E+10 | 1.E+06 | 0.E+00 | 0.E+00 | 0.E+00 | 1.E+05 | 1.E+05 | 0.E+00 | 0.E+00 | 0.E+00 | 0.E+00 |  |  |
| K69 | 1.E+10 | 1.E+10 | 1.E+10 | 1.E+10 | 1.E+10 | 1.E+10 | 1.E+06 | 0.E+00 | 0.E+00 | 0.E+00 | 1.E+05 | 1.E+05 | 0.E+00 | 0.E+00 | 0.E+00 | 0.E+00 |  |  |
| K14 | 1.E+10 | 1.E+10 | 1.E+10 | 1.E+10 | 1.E+10 | 1.E+10 | 1.E+06 | 0.E+00 | 0.E+00 | 0.E+00 | 1.E+05 | 1.E+05 | 0.E+00 | 0.E+00 | 0.E+00 | 0.E+00 | 1-2 |  |
| K29 | 1.E+10 | 1.E+10 | 1.E+10 | 1.E+10 | 1.E+10 | 1.E+10 | 1.E+06 | 0.E+00 | 0.E+00 | 0.E+00 | 1.E+05 | 1.E+05 | 0.E+00 | 0.E+00 | 0.E+00 | 0.E+00 |  |  |
| K53 | 1.E+10 | 1.E+10 | 1.E+10 | 1.E+10 | 1.E+10 | 1.E+10 | 1.E+07 | 0.E+00 | 0.E+00 | 0.E+00 | 1.E+05 | 1.E+05 | 0.E+00 | 0.E+00 | 0.E+00 | 0.E+00 |  |  |
| K65 | 1.E+10 | 1.E+10 | 1.E+10 | 1.E+10 | 1.E+10 | 1.E+10 | 1.E+06 | 0.E+00 | 0.E+00 | 0.E+00 | 1.E+05 | 1.E+05 | 0.E+00 | 0.E+00 | 0.E+00 | 0.E+00 |  |  |
| K6 | 1.E+10 | 1.E+10 | 1.E+10 | 1.E+10 | 1.E+10 | 1.E+10 | 1.E+07 | 0.E+00 | 0.E+00 | 0.E+00 | 1.E+05 | 1.E+05 | 0.E+00 | 0.E+00 | 0.E+00 | 0.E+00 | 1-3 |  |
| K8 | 1.E+10 | 1.E+10 | 1.E+10 | 1.E+10 | 1.E+10 | 1.E+10 | 1.E+07 | 0.E+00 | 0.E+00 | 0.E+00 | 1.E+05 | 1.E+05 | 0.E+00 | 0.E+00 | 0.E+00 | 0.E+00 |  |  |
| K7 | 1.E+10 | 1.E+10 | 1.E+10 | 1.E+10 | 1.E+10 | 1.E+10 | 1.E+07 | 0.E+00 | 0.E+00 | 0.E+00 | 1.E+05 | 1.E+05 | 0.E+00 | 0.E+00 | 0.E+00 | 0.E+00 |  |  |
| K61 | 1.E+10 | 1.E+10 | 1.E+10 | 1.E+10 | 1.E+10 | 1.E+10 | 1.E+07 | 0.E+00 | 0.E+00 | 0.E+00 | 1.E+05 | 1.E+05 | 0.E+00 | 0.E+00 | 0.E+00 | 0.E+00 |  |  |
| K22 | 1.E+10 | 1.E+10 | 1.E+10 | 1.E+10 | 1.E+10 | 1.E+10 | 1.E+10 | 0.E+00 | 0.E+00 | 0.E+00 | 1.E+05 | 1.E+05 | 0.E+00 | 0.E+00 | 0.E+00 | 0.E+00 | 1-4 |  |
| K58 | 1.E+10 | 1.E+10 | 1.E+10 | 1.E+10 | 1.E+10 | 1.E+10 | 1.E+10 | 0.E+00 | 0.E+00 | 0.E+00 | 1.E+05 | 1.E+05 | 0.E+00 | 0.E+00 | 0.E+00 | 0.E+00 |  |  |
| K1 | 1.E+10 | 1.E+10 | 1.E+10 | 1.E+10 | 1.E+10 | 1.E+10 | 1.E+10 | 0.E+00 | 0.E+00 | 0.E+00 | 1.E+05 | 1.E+05 | 0.E+00 | 0.E+00 | 0.E+00 | 0.E+00 |  |  |
| K10 | 1.E+10 | 1.E+10 | 1.E+10 | 1.E+10 | 1.E+10 | 1.E+10 | 1.E+07 | 0.E+00 | 0.E+00 | 0.E+00 | 1.E+05 | 1.E+05 | 0.E+00 | 0.E+00 | 0.E+00 | 0.E+00 |  |  |
| K46 | 1.E+10 | 1.E+10 | 1.E+10 | 1.E+10 | 1.E+10 | 1.E+10 | 1.E+10 | 0.E+00 | 0.E+00 | 0.E+00 | 1.E+05 | 1.E+05 | 0.E+00 | 0.E+00 | 0.E+00 | 0.E+00 |  |  |
| K26 | 1.E+10 | 1.E+10 | 1.E+10 | 1.E+10 | 1.E+10 | 1.E+10 | 1.E+07 | 0.E+00 | 0.E+00 | 0.E+00 | 1.E+05 | 1.E+05 | 0.E+00 | 0.E+00 | 0.E+00 | 0.E+00 | 1-5 |  |
| K9 | 1.E+10 | 1.E+10 | 1.E+10 | 1.E+10 | 1.E+10 | 1.E+10 | 1.E+07 | 0.E+00 | 0.E+00 | 0.E+00 | 1.E+05 | 1.E+05 | 0.E+00 | 0.E+00 | 0.E+00 | 0.E+00 |  |  |
| K54 | 1.E+10 | 1.E+10 | 1.E+10 | 1.E+10 | 1.E+10 | 1.E+10 | 1.E+10 | 0.E+00 | 0.E+00 | 0.E+00 | 1.E+05 | 1.E+05 | 0.E+00 | 0.E+00 | 0.E+00 | 0.E+00 | 1-6 |  |
| K56 | 1.E+10 | 1.E+10 | 1.E+10 | 1.E+10 | 1.E+10 | 1.E+10 | 1.E+07 | 0.E+00 | 0.E+00 | 0.E+00 | 1.E+05 | 1.E+05 | 0.E+00 | 0.E+00 | 0.E+00 | 0.E+00 | 1-7 |  |
| K47 | 1.E+10 | 1.E+10 | 1.E+10 | 1.E+10 | 1.E+10 | 1.E+10 | 1.E+10 | 0.E+00 | 0.E+00 | 0.E+00 | 1.E+05 | 1.E+05 | 0.E+00 | 0.E+00 | 0.E+00 | 0.E+00 | 1-8 |  |
| K16 | 0.E+00 | 0.E+00 | 0.E+00 | 0.E+00 | 0.E+00 | 0.E+00 | 0.E+00 | 1.E+10 | 1.E+10 | 1.E+10 | 0.E+00 | 0.E+00 | 0.E+00 | 0.E+00 | 0.E+00 | 0.E+00 | 2-1 | MT2 |
| K17 | 0.E+00 | 0.E+00 | 0.E+00 | 0.E+00 | 0.E+00 | 0.E+00 | 0.E+00 | 1.E+10 | 1.E+10 | 1.E+10 | 0.E+00 | 0.E+00 | 0.E+00 | 0.E+00 | 0.E+00 | 0.E+00 |  |  |
| K33 | 0.E+00 | 0.E+00 | 0.E+00 | 0.E+00 | 0.E+00 | 0.E+00 | 0.E+00 | 1.E+10 | 1.E+10 | 1.E+10 | 0.E+00 | 0.E+00 | 0.E+00 | 0.E+00 | 0.E+00 | 0.E+00 |  |  |
| K34 | 0.E+00 | 0.E+00 | 0.E+00 | 0.E+00 | 0.E+00 | 0.E+00 | 0.E+00 | 1.E+10 | 1.E+10 | 1.E+10 | 0.E+00 | 0.E+00 | 0.E+00 | 0.E+00 | 0.E+00 | 0.E+00 |  |  |
| K35 | 0.E+00 | 0.E+00 | 0.E+00 | 0.E+00 | 0.E+00 | 0.E+00 | 0.E+00 | 1.E+10 | 1.E+10 | 1.E+10 | 0.E+00 | 0.E+00 | 0.E+00 | 0.E+00 | 0.E+00 | 0.E+00 |  |  |
| K39 | 0.E+00 | 0.E+00 | 0.E+00 | 0.E+00 | 0.E+00 | 0.E+00 | 0.E+00 | 1.E+10 | 1.E+10 | 1.E+10 | 0.E+00 | 0.E+00 | 0.E+00 | 0.E+00 | 0.E+00 | 0.E+00 |  |  |
| K40 | 0.E+00 | 0.E+00 | 0.E+00 | 0.E+00 | 0.E+00 | 0.E+00 | 0.E+00 | 1.E+10 | 1.E+10 | 1.E+10 | 0.E+00 | 0.E+00 | 0.E+00 | 0.E+00 | 0.E+00 | 0.E+00 |  |  |
| K41 | 0.E+00 | 0.E+00 | 0.E+00 | 0.E+00 | 0.E+00 | 0.E+00 | 0.E+00 | 1.E+10 | 1.E+10 | 1.E+10 | 0.E+00 | 0.E+00 | 0.E+00 | 0.E+00 | 0.E+00 | 0.E+00 |  |  |
| K36 | 0.E+00 | 0.E+00 | 0.E+00 | 0.E+00 | 0.E+00 | 0.E+00 | 0.E+00 | 1.E+10 | 1.E+10 | 1.E+10 | 0.E+00 | 0.E+00 | 0.E+00 | 0.E+00 | 0.E+00 | 0.E+00 | 2-2 |  |
| K42 | 0.E+00 | 0.E+00 | 0.E+00 | 0.E+00 | 0.E+00 | 0.E+00 | 0.E+00 | 1.E+09 | 1.E+09 | 1.E+09 | 0.E+00 | 0.E+00 | 0.E+00 | 0.E+00 | 0.E+00 | 0.E+00 |  |  |
| K15 | 0.E+00 | 0.E+00 | 0.E+00 | 0.E+00 | 0.E+00 | 0.E+00 | 0.E+00 | 1.E+10 | 1.E+09 | 1.E+09 | 0.E+00 | 0.E+00 | 0.E+00 | 0.E+00 | 0.E+00 | 0.E+00 | 2-3 |  |
| K18 | 0.E+00 | 0.E+00 | 0.E+00 | 0.E+00 | 0.E+00 | 0.E+00 | 0.E+00 | 1.E+10 | 1.E+10 | 1.E+10 | 0.E+00 | 0.E+00 | 0.E+00 | 0.E+00 | 0.E+00 | 0.E+00 | 2-4 |  |
| K20 | 0.E+00 | 0.E+00 | 0.E+00 | 0.E+00 | 0.E+00 | 0.E+00 | 0.E+00 | 1.E+10 | 1.E+10 | 1.E+10 | 0.E+00 | 0.E+00 | 0.E+00 | 0.E+00 | 0.E+00 | 0.E+00 | 2-5 |  |
| K37 | 0.E+00 | 0.E+00 | 1.E+08 | 1.E+08 | 1.E+09 | 0.E+00 | 0.E+00 | 1.E+10 | 1.E+10 | 1.E+10 | 0.E+00 | 0.E+00 | 0.E+00 | 0.E+00 | 0.E+00 | 0.E+00 | 2-6 |  |
| K43 | 0.E+00 | 0.E+00 | 1.E+09 | 1.E+09 | 1.E+09 | 0.E+00 | 0.E+00 | 1.E+10 | 1.E+10 | 1.E+10 | 0.E+00 | 0.E+00 | 0.E+00 | 0.E+00 | 0.E+00 | 0.E+00 |  |  |
| K44 | 0.E+00 | 0.E+00 | 0.E+00 | 0.E+00 | 0.E+00 | 0.E+00 | 0.E+00 | 1.E+09 | 1.E+09 | 1.E+09 | 0.E+00 | 0.E+00 | 0.E+00 | 0.E+00 | 0.E+00 | 0.E+00 | 2-7 |  |
| K38 | 0.E+00 | 0.E+00 | 0.E+00 | 0.E+00 | 0.E+00 | 0.E+00 | 0.E+00 | 1.E+10 | 1.E+10 | 1.E+10 | 0.E+00 | 0.E+00 | 0.E+00 | 0.E+00 | 0.E+00 | 0.E+00 | 2-8 |  |
| K45 | 0.E+00 | 0.E+00 | 0.E+00 | 0.E+00 | 0.E+00 | 0.E+00 | 0.E+00 | 1.E+10 | 1.E+10 | 1.E+10 | 0.E+00 | 0.E+00 | 0.E+00 | 0.E+00 | 0.E+00 | 0.E+00 |  |  |
| K11 | 1.E+09 | 1.E+09 | 0.E+00 | 0.E+00 | 0.E+00 | 1.E+09 | 1.E+09 | 0.E+00 | 0.E+00 | 0.E+00 | 1.E+09 | 1.E+09 | 1.E+05 | 1.E+09 | 1.E+08 | 1.E+08 |  | MT3 |
| K30 | 1.E+09 | 1.E+09 | 0.E+00 | 0.E+00 | 0.E+00 | 1.E+09 | 1.E+09 | 0.E+00 | 0.E+00 | 0.E+00 | 1.E+10 | 1.E+10 | 1.E+06 | 1.E+08 | 1.E+08 | 1.E+08 |  |  |
| K31 | 1.E+09 | 1.E+09 | 0.E+00 | 0.E+00 | 0.E+00 | 1.E+09 | 1.E+09 | 0.E+00 | 0.E+00 | 0.E+00 | 1.E+10 | 1.E+10 | 1.E+06 | 1.E+08 | 1.E+08 | 1.E+08 |  |  |
| K32 | 1.E+10 | 1.E+10 | 0.E+00 | 0.E+00 | 0.E+00 | 1.E+10 | 1.E+10 | 0.E+00 | 0.E+00 | 0.E+00 | 1.E+10 | 1.E+10 | 1.E+06 | 1.E+08 | 1.E+08 | 1.E+08 |  |  |
| K19 | 0.E+00 | 0.E+00 | 0.E+00 | 0.E+00 | 0.E+00 | 0.E+00 | 0.E+00 | 1.E+09 | 1.E+09 | 1.E+09 | 0.E+00 | 0.E+00 | 0.E+00 | 0.E+00 | 0.E+00 | 0.E+00 |  | MT4 |

**Supplement Figure Legends**

Supplement Fig. 1: Distance matrix of the 59 phage isolates showing 4 different major types and possible subtypes based on their genomic similarities after pairwise analyses.

Supplement Fig. 2: Mauve alignments of phage genomes. A. Reference genomes of MT1. B. Reference genomes of MT2. C. Four reference genomes representing MT1 (K47), MT2 (K37), MT3 (K30) and MT4 (K19).

Supplement Fig. 3: Proteome comparison of 4 reference phages from 4 MTs: K47 – MT1, K32 – MT2, K30 – MT3 and K19 from MT4 with their top BLASTn hits from NCBI.

Supplement Fig. 4: Simulated EcoRV restriction profiles of phage K47 linear and circular genome by Geneious software. L: 1-kb ladder.
